# Pretargeted ^177^Lu/^225^Ac combination therapy of colorectal cancer

**DOI:** 10.7150/thno.126399

**Published:** 2026-04-08

**Authors:** Sara S. Rinne, Niloufar Salehi, Brett A. Vaughn, Daniela Burnes Vargas, Hongfen Guo, Sang Gyu Lee, Claire Vanpouille-Box, Ileana C. Miranda, Brian W. Miller, Edward K. Fung, Steven M. Larson, Darren R. Veach, Nai-Kong V. Cheung, Sarah M. Cheal

**Affiliations:** 1 Department of Radiology, Weill Cornell Medicine, New York, NY, 10065, USA.; 2 Department of Pediatrics, Memorial Sloan Kettering Cancer Center, New York, NY, 10065, USA.; 3 Uppsala University, Department of Immunology, Genetics and Pathology, Uppsala University, Uppsala, Sweden.; 4 Molecular Pharmacology Program, Memorial Sloan Kettering Cancer Center, New York, NY, 10065, USA.; 5 Department of Radiology, Memorial Sloan Kettering Cancer Center, New York, NY, 10065, USA.; 6 Department of Radiation Oncology, Weill Cornell Medicine, New York, NY, 10065, USA.; 7 Laboratory of Comparative Pathology, Weill Cornell Medicine, Memorial Sloan Kettering Cancer Center, and The Rockefeller University, New York, NY, 10065, USA.; 8 Department of Radiation Oncology and Medical Imaging, University of Arizona, Tucson, AZ, 85724, USA.

**Keywords:** ^177^Lu, ^225^Ac, combination therapy, pretargeting, DOTA-PRIT

## Abstract

**Rationale:**

Combining targeted alpha and beta therapy may address challenges such as toxicity, treatment resistance, and tumor heterogeneity. We evaluated the feasibility and therapeutic effectiveness of a DOTA-PRIT approach using a ^177^Lu/^225^Ac radioisotope cocktail, directly compared with monotherapies targeting GPA33 in human colorectal cancer (CRC) xenografts in mice.

**Methods:**

A three-step pretargeting regimen was employed: an anti-GPA33/anti-DOTA bispecific antibody (BsAb), a dendrimeric clearing agent, and radioligands labeled with ^177^Lu, ^225^Ac, alone or in combination. Serial biodistribution studies in GPA33(+) SW1222 xenografts evaluated how co-injection of ^177^Lu and ^225^Ac radioligands affected tumor uptake and biodistribution. iQID digital autoradiography was used to visualize isotope distribution in tumor and kidney samples. Mice bearing SW1222 and LS174T xenografts received mono- or combination-therapy regimens delivering 37-38 Gy to tumors. Dose-escalation studies, histopathology, and qPCR analysis of DNA-damage and apoptosis-related genes were also performed.

**Results:**

Biodistribution and autoradiography confirmed that the ^177^Lu- and ^225^Ac-labeled ligand effectively bound to pretargeted GPA33(+) xenografts when co-administered. High therapeutic indices were maintained across treatment groups, with autoradiography showing general overlap of co-injected probes. Combination therapy demonstrated comparable efficacy to monotherapies. At 150 d post-treatment, no treatment group had reached median survival; 5/9 mice receiving the cocktail (62.9 MBq ^177^Lu + 18.5 kBq ^225^Ac) were alive, including two tumor-free. In comparison, 4/8 mice in the ^177^Lu group and 8/10 in the ^225^Ac group survived, with 3 and 5 tumor-free animals, respectively. Combination therapy was well tolerated, showing no significant adverse effects on body weight or blood cell counts compared to healthy controls. Combined administration was safe up to 62.9 MBq ^177^Lu + 37 kBq ^225^Ac, resulting in 10/10 histological cures.

**Conclusions:**

Our findings confirm the feasibility of a combined ^177^Lu and ^225^Ac DOTA-PRIT in mice with established SW1222 xenografts, demonstrating tolerability and effectiveness comparable to monotherapy at equivalent average absorbed tumor doses.

## Introduction

Radiotheranostics have been transformative in the treatment of advanced cancers, particularly gastroenteropancreatic neuroendocrine tumors and metastatic castration-resistant prostate cancer. However, challenges remain, with objective responses in only 30-60% of patients 1. To enhance effectiveness, significant efforts are underway to combine radiotheranostics with other synergistic therapeutic modalities 2.

One promising strategy involves expanding the therapeutic window of radiotheranostics by better tailoring the radiophysical and radiobiological characteristics to the patient's disease phenotype 3. This is especially relevant in the treatment of advanced (micro)metastatic disease, where beta (β)-particles may be mismatched with small metastases (millimeter-sized tumor lesions) that lead to inefficient delivery of radiation 4. It has been hypothesized that to address both interlesional and intralesional heterogeneity, a combination of an alpha (⍺)-emitter (short-range particle) and a β-emitter (long-range particle) may be beneficial when administered together. This approach may enhance efficacy due to the complementary physical and radiobiological properties of β- and ⍺-emitters 3.

^225^Ac is an ⍺-emitter (emitting four ⍺-particles per decay down to stable ^209^Bi, with energies ranging from 5.8 to 8.4 MeV and a tissue range of 47 to 85 µm) with a half-life of 9.92 days 5. ^177^Lu is a β-emitter (energies ranging from 176 to 497 keV, an average tissue range of 0.23 mm, maximum 1.7 mm) with a half-life of 6.65 days 6. While enhancing tumor cure probability is a theoretical benefit of combined ⍺/β-therapy, clinical toxicities can pose major limitations for monotherapies. For example, ^225^Ac-PSMA-617/^177^Lu-PSMA-617 is being explored following failure of ^177^Lu-PSMA-617 7, and as a strategy to enhance efficacy while minimizing dose-limiting xerostomia during ^225^Ac-PSMA-617 therapy. A recent update from Novruzov *et al.* on preliminary findings from a phase 3, single-center, prospective, randomized, two-arm controlled study comparing ^225^Ac/^177^Lu-labeled PSMA (PSMA tandem treatment) with the current standard of care (docetaxel) revealed that PSMA tandem treatment minimized adverse effects while preserving treatment efficacy 8. Alternative forms of tandem ⍺/β-therapy include ^225^Ac-J591 and ^177^Lu-PSMA-I&T (NCT04886986) and ^177^Lu-PSMA-I&T in combination with Xofigo® (AlphaBet, NCT05383079).

**Figure 1:**
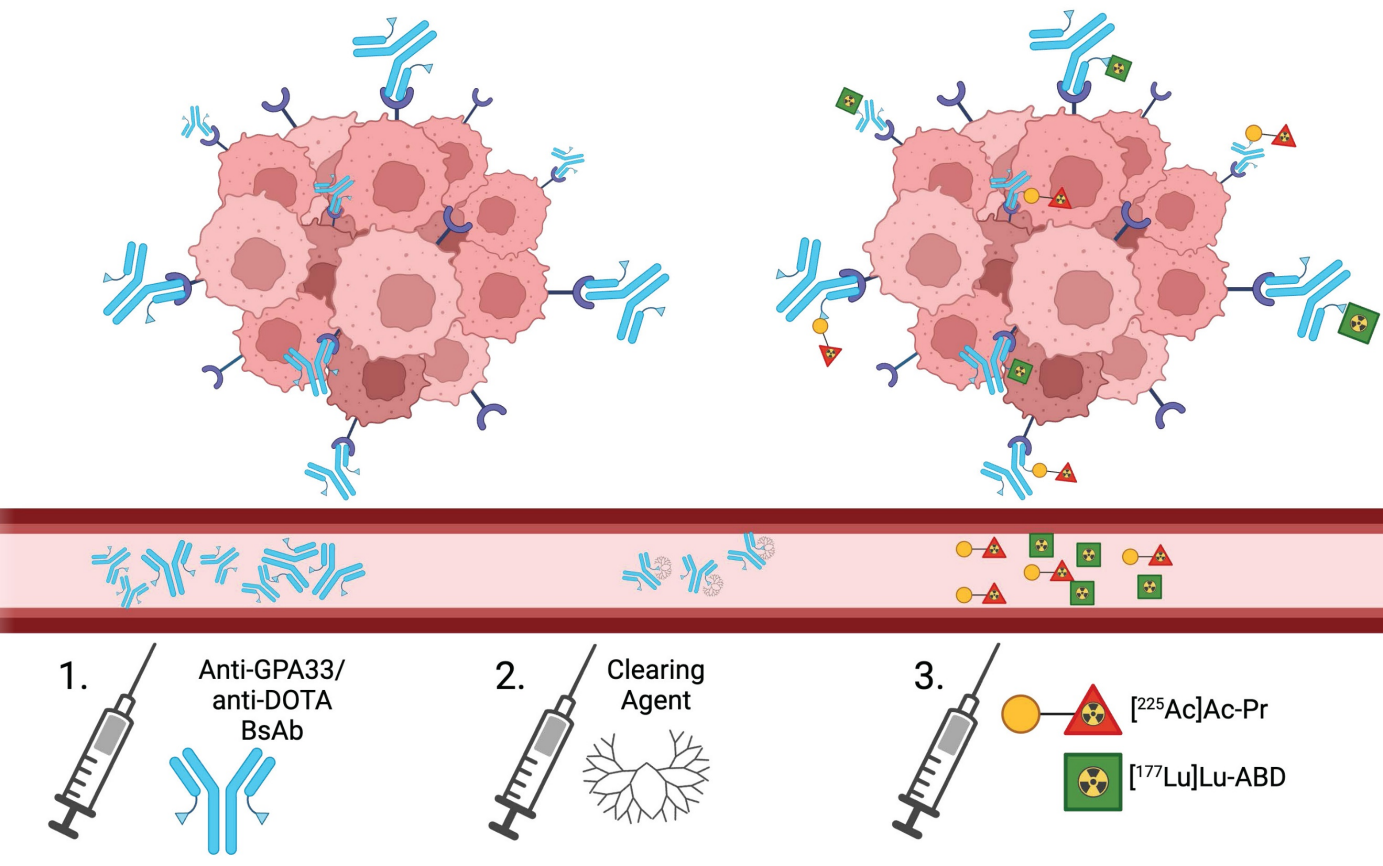
Schematic overview of combined ^177^Lu/^225^Ac-DOTA-PRIT. The clearing agent is administered 20 h after BsAb injection and 4 h prior to radioligand administration.

We have developed a highly modular anti-tumor antigen/anti-2,2′,2”,2”'-(1,4,7,10-tetraazacyclododecane-1,4,7,10-tetrayl)tetraacetic acid (DOTA)-hapten bispecific antibody (BsAb) platform for tumor-specific pretargeted radioimmunotherapy (PRIT), capable of delivering high doses of ⍺ or β-radiation 9-11. In the current study, we evaluated a combination PRIT approach using ^225^Ac/^177^Lu in a GPA33-expressing human colorectal cancer (CRC) model (Figure 1). Anti-GPA33 DOTA-PRIT is a well-established system, offering a strong foundation for exploring new dosing regimens. Similar to a recently reported preclinical tandem ^225^Ac/^177^Lu study 12, we hypothesized that the distinct therapeutic decay properties of radionuclides would enhance radiation dose delivery to tumors without compromising the therapeutic indices (TIs) to blood (red marrow) and kidney.

To test this, we conducted serial biodistribution, dosimetry, and dual-isotope autoradiography studies to confirm that co-injection of GPA33-pretargeted ^225^Ac- and ^177^Lu-DOTA radioligands is feasible and does not result in competitive inhibition. We then compared the efficacy of ^225^Ac/^177^Lu combination therapy with single-isotope treatments and investigated dose-toxicity relationships of various ^225^Ac/^177^Lu cocktails. Finally, we used real-time quantitative PCR assay (qRT-PCR) and immunofluorescence staining of phosphorylated histone γH2AX to gain insight into mechanisms of DNA damage and cell death.

## Material and Methods

### General

GPA33-expressing SW1222 and LS174T human CRC cell lines were obtained from ATCC (Manassas, VA, USA). GPA33 antigen density was quantified by flow cytometry using Quantum Simply Cellular calibration beads (Bangs Laboratories, Inc., Fishers, IN, USA), resulting in 108,531 sites per cell for SW1222 and 189,119 sites per cell for LS174T. SW1222 were cultured in IMDM media (Gibco, Waltham, MA, USA) supplemented with 10% FBS, 200 mM Glutamate and 1% penicillin/streptomycin. LS174T cells were maintained in RPMI1640 media (Gibco, Waltham, MA, USA) supplemented with 10% FBS, 200 mM glutamate and 1% penicillin/streptomycin.

The IgG-scFv bispecific anti-GPA33/anti-DOTA antibody was produced according to previously described methods 13. Dendrimeric clearing agent was synthesized according to Cheal *et al.* 14.

### Radiolabeling

[^177^Lu]Lu-aminobenzyl-DOTA ([^177^Lu]Lu-ABD) and [^225^Ac]Ac-Proteus (LuDOTA-Bn-PEG_4_-[^225^Ac]Ac-DO3A, hereafter [^225^Ac]Ac-Pr) were labeled according to previously published protocols 11, 15. ^177^LuCl_3_ was purchased from ITM Radiopharma (Munich, Germany). ^225^Ac was supplied by the U.S. Department of Energy Isotope Program managed by the Office of Isotope R&D and Production.

### Animal experiments

All animal experiments were approved by the Institutional Animal Care and Use Committee at Weill Cornell Medicine and conducted in female athymic nude mice (Taconic or Jackson Laboratories) with SW1222 or LS174T CRC xenografts. To establish the xenograft models, mice were inoculated subcutaneously with 5 million SW1222 or LS174T cells 10 days before the start of the experiments.

### DOTA-PRIT regimen

All three DOTA-PRIT reagents were administered intravenously. Tumor-bearing mice received 250 µg (1.19 nmol) of anti-GPA33/anti-DOTA BsAb via IV injection 24 h prior to radioligand administration. Four hours before the radioligand injection, mice were administered 25 µg (2.76 nmol) of dendrimeric clearing agent. The total administered mass of radioligand, independent of the radiolabel, was 700 pmol. This pretargeting regimen consistently achieved efficient tumor targeting (approximately 10-20 %IA/g) while maintaining high tumor-to-blood and tumor-to-kidney ratios, establishing the basis for high TIs. For mice receiving the ^177^Lu/^225^Ac cocktail, a mixture containing 350 pmol of [^177^Lu]Lu-ABD and 350 pmol [^225^Ac]Ac-Pr was administered.

### Biodistribution and tumor dosimetry

Biodistribution studies were performed to determine how co-injection of the different radioligands affects *in vivo* PRIT performance. Mice bearing SW1222 xenografts (*n* = 5/group) were treated with DOTA-PRIT according to the protocol described above. Mice received one of four different radioligand or radioligand combination treatments: (1) [^177^Lu]Lu-ABD (700 pmol, 1.85 MBq), (2) [^177^Lu]Lu-ABD (350 pmol, 1.85 MBq) + ^139^La-Pr (350 pmol), (3) [^225^Ac]Ac-Pr (700 pmol, 37 kBq), or (4) [^225^Ac]Ac-Pr (350 pmol, 37 kBq) + ^175^Lu-ABD (350 pmol). In the combination groups, one ligand was labeled with non-radioactive surrogate (^175^Lu instead of ^177^Lu or ^139^La instead of ^225^Ac) in place of the corresponding radionuclide isotope. Mice were euthanized at 2 h, 24 h, 72 h and 120 h post radioligand injection via CO_2_ asphyxiation. Blood was collected by heart puncture, and organs were harvested, weighed, and assayed for radioactivity content using an automated gamma counter.

To calculate dosimetry, time-integrated activities were calculated by trapezoidal integration over the measured time points from the average time-activity curves for each cohort. Physical decay only was assumed after the last time point at 120 h postinjection and the integral calculated to infinity. β and ⍺ emissions were all assumed to be absorbed locally in each organ and dose in Gy per administered activity estimated. For ^225^Ac, it was assumed that there was no drift and all progeny nuclides decayed locally.

### iQID autoradiography

Mice bearing SW1222 or LS174T xenografts were injected with ^177^Lu-, ^225^Ac-, or ^177^Lu/^225^Ac-DOTA-PRIT according to the regimen described above. Injected activities were 1.85 MBq of [^177^Lu]Lu-ABD and 37 kBq [^225^Ac]Ac-Pr for single-isotope and combination groups. Mice were euthanized 24 h post-injection. Tumors and kidneys were harvested and immediately embedded in OCT compound and frozen. Tissue sections (7 µm thick) were mounted onto Gadolinium-Oxysulfide (Gadox) phosphor scintillation paper, which is sensitive to both ⍺ and β particles (QScint-02-0001). Adjacent sections were placed on glass slides for corresponding H&E staining. Autoradiography was performed using the iQID camera (ionizing-radiation quantum imaging detector; QScint Imaging Solutions) 16. Tissue sections were imaged for 24 h (SW1222) and 216 h (LS174T). The extended acquisition time for LS174T was solely due to imaging device availability and to obtain higher counts per pixel. Higher-resolution images are being analyzed separately as part of an independent study. List mode data for each event were used to generate digital autoradiographs, where pixel values represent the number of detected events in that region. ⍺ and β particle events were distinguished based on a combination of event size and the total signal within each scintillation event. Threshold cuts were applied to the list mode data to generate three image types: total counts, ⍺-only, and β-only digital autoradiographs. Tissue sections labeled with ^177^Lu only and ^225^Ac only were used as controls to validate the threshold settings.

### *In vivo* therapy

Therapy studies were conducted in mice bearing SW1222 (starting volume 140 ± 60 mm^3^) or LS174T (starting volume 176 ± 56 mm^3^) colorectal cancer xenografts. Two independent therapy studies were performed, in which mice received treatment according to the DOTA-PRIT regimen, along with appropriate controls (*n* = 5-10 mice/group).

In Study 1, mice were treated with an estimated absorbed tumor dose of 37-38 Gy. Injected activities were based on dosimetry calculations from biodistribution data, and the relative biological effectiveness (RBE) was not considered. In the combination treatment group, mice received one cycle of 62.9 MBq [^177^Lu]Lu-ABD + 18.5 kBq of [^225^Ac]Ac-Pr, each delivering 19 Gy to the tumor. Monotherapy groups received either 66.6 MBq of [^177^Lu]Lu-ABD or 74 kBq of [^225^Ac]Ac-Pr.

In the follow-up Study 2, mice were administered a fixed dose of [^177^Lu]Lu-ABD (62.9 MBq) in combination with increasing amounts of [^225^Ac]Ac-Pr (18.5 kBq, 37 kBq or 74 kBq). For the LS174T model, mice received one cycle of either the combination therapy (62.9 MBq [^177^Lu]Lu-ABD + 37 kBq [^225^Ac]Ac-Pr), 66.6 MBq [^177^Lu]Lu-ABD alone, or 74 kBq [^225^Ac]Ac-Pr alone.

Animals were monitored for 150 days, with tumor measurements twice per week, weekly bodyweight measurements, and periodic blood draws for CBC. Predetermined study endpoints included tumor volume >1500 mm^3^, ulcerated tumor, or >20% loss of bodyweight within a week. After 150 days, all survivors were submitted to the Laboratory of Comparative Pathology of Weill Cornell Medicine, MSKCC, and The Rockefeller University, for complete necropsy and pathological evaluation by a board-certified veterinary pathologist (see Supplemental Information for details).

### Toxicity studies

Toxicity studies were conducted in tumor-free athymic nude mice, which were littermates of those used in Studies 1 and 2. These mice received the same treatment regimens as described for Studies 1 and 2 and were monitored up to 150 days. At the end of the observation period, all animals were submitted to the Laboratory of Comparative Pathology of Weill Cornell Medicine, MSKCC, and The Rockefeller University for complete necropsy and pathological evaluation by a board-certified veterinary pathologist (see Supplemental Information for details).

### DNA damage and apoptosis PCR array

RT^2^ profiler PCR Array (Qiagen, Hilden, Germany) was used to study the expression of DNA damage and apoptosis related genes in SW1222 xenograft samples following DOTA-PRIT treatment. cDNA for the PCR arrays was prepared from RNA extracted from flash frozen SW1222 xenografts from mice. Mice were treated with DOTA-PRIT 24 h prior to tumor harvest (2 samples per treatment group). RNA extraction was performed using the RNeasy Mini kit (Qiagen, Hilden, Germany). Conversion to cDNA was done using the cDNA SuperScript™ VILO™ cDNA Synthesis Kit (Invitrogen) using 1 µg of RNA. Relative expression was characterized (RT² SYBR Green Fluor qPCR Mastermix, Qiagen) in 96-well microtiter plates (plate details in Fig S1,S2) on 7500 RT-PCR system (Applied Biosystems) operated by embedded software v.2.3. Due to insufficient RNA yield from one of the samples in the ^225^Ac-treated group, only a single data point was obtained for certain genes in that group. The fold change in gene expression was calculated using the equation 2^(-ΔΔCT)^.

Genes for which one of the C_T_-values was undetermined were excluded from analysis. If the fold change was greater than 1, the result was considered an up-regulation. If the fold change was less than 1 the result was considered a down regulation. Genes were also excluded from further analysis if there was a mismatch in fold change between housekeeping genes. Data was displayed as a heat map using GraphPad Prism normalized to the housekeeping gene with the most consistent C_T_ values across all samples (*RPLP0*).

### Immunofluorescence staining

Automated multiplex IF staining was performed using Leica Bond BX staining system. Mice bearing SW1222 xenografts were treated with therapeutic doses of DOTA-PRIT 24 h prior to tumor harvest (2 samples per treatment group). Tumors were harvested, paraffin-embedded, and stained for Ki67 (Cell signaling technology, 9027), cleaved caspase-3 (Cell Signaling Technologies, 9667) and p-H2AX (Abcam, ab11174). A detailed description of the staining protocol is provided in the Supplementary Materials. Slides were scanned using a Pannoramic Scanner (3DHistech, Budapest, Hungary) using a 20x/0.8NA objective. Images were analyzed using QuPath v0.4.4 for MacOS.

### Statistics

Graphpad Prism 10 for macOS was used for statistical analysis. Statistical significance (p < 0.05) for biodistribution experiments was determined using one-way ANOVA with post-hoc t-test adjusted for multiple comparison with Tukey. For therapy studies, survival curves were compared with Log-rank (Mantel-Cox) test.

## Results

### Biodistribution and dosimetry

**Figure 2:**
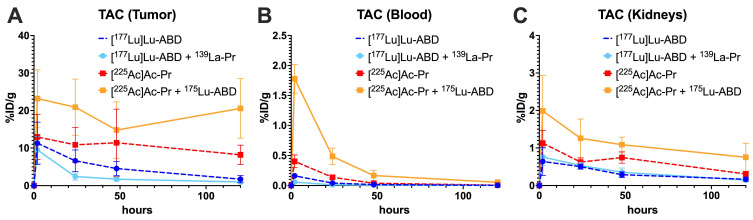
Uptake of [^177^Lu]Lu-ABD and [^225^Ac]Ac-Pr in SW1222 tumors, blood, and kidneys were assessed after pretargeting with anti-GPA33 BsAb. Mice (*n* = 4-5/group) were administered either 700 pmol [^177^Lu]Lu-ABD (dashed dark blue) or [^225^Ac]Ac-Pr (dashed red) alone, or a cocktail containing 350 pmol of one radioligand and 350 pmol of non-radiolabeled ligand of the other type (light blue or orange). *n* = 4-5 animals/datapoint. Average ± 1SD. Full numerical data are provided in Tables S1 and S2.

**Figure 3:**
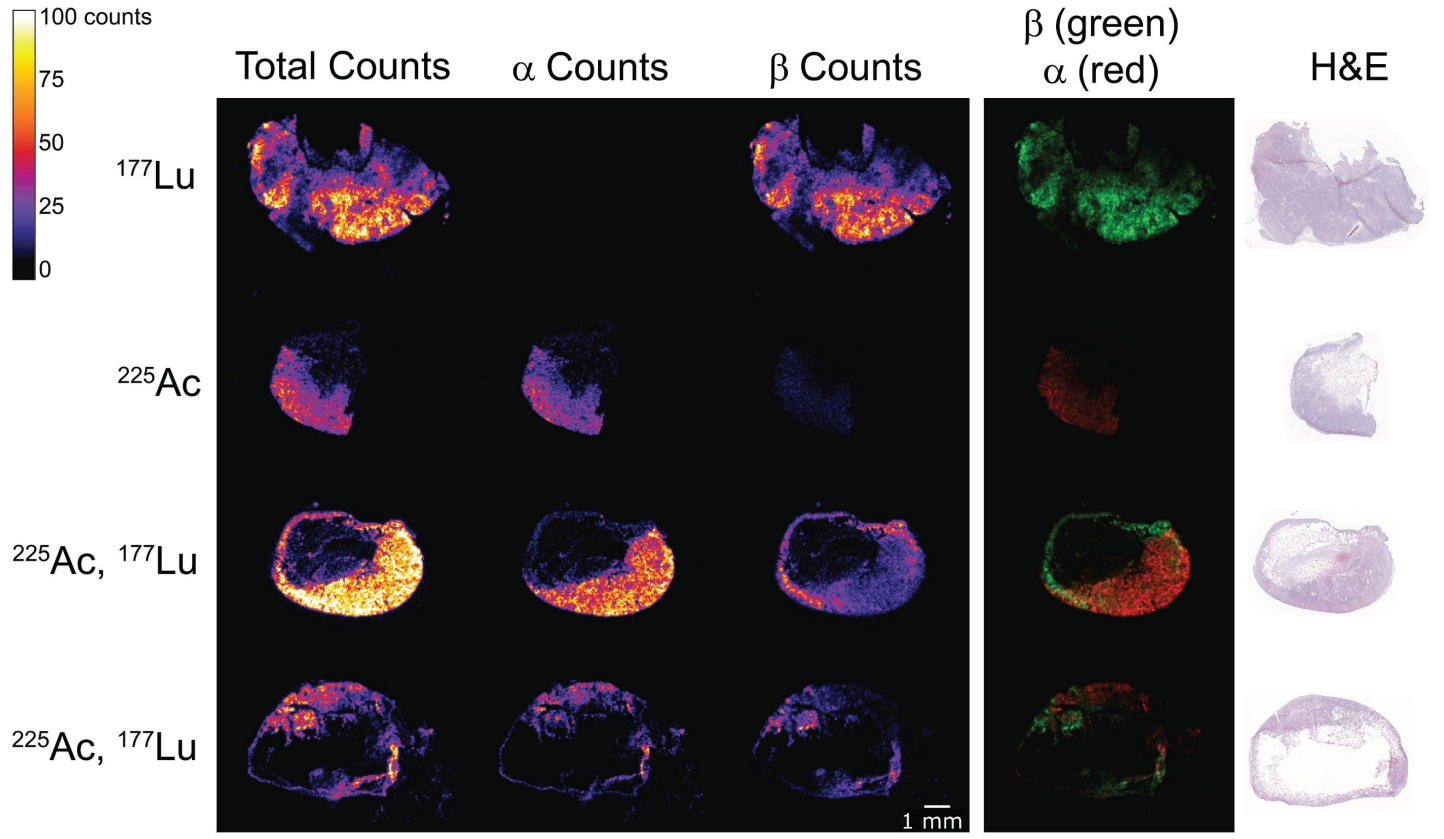
Digital autoradiography of SW1222 xenografts pretargeted with anti-GPA33 BsAb. Samples were collected 24 h after administration of [^225^Ac]Ac-Pr and [^177^Lu]Lu-ABD. Mice received either 700 pmol of [^177^Lu]Lu-ABD (1.85 MBq, first row), 700 pmol of [^225^Ac]Ac-Pr (37 kBq, second row), or a combination (cocktail) of 350 pmol of each radioligand at the same respective activity levels (third and fourth row, tumors collected from two different mice). The first column displays the combined signal from both ^177^Lu and ^225^Ac. The second column shows the ^225^Ac signal only (alphas), while the third column shows the ^177^Lu signal only (betas). Note that ^225^Ac daughter isotopes (^213^Bi and ^209^Pb) emit betas, which can be faintly seen in the third column of the ^225^Ac-only tumor sample. The fourth column shows an overlay of ^225^Ac alphas (red) and ^177^Lu betas (green). Hematoxylin and eosin (H&E) staining of consecutive tissue sections is shown in the last column.

Biodistribution analysis showed that co-administration of the radioligands did not prevent tumor binding (Figure 2). There was a statistically significant decrease in in area under the curve (AUC) of [^177^Lu]Lu-ABD tumor uptake alone (dashed dark blue) versus when ^139^La-Pr was added (light blue) (Table S3), but no significant difference was observed between the [^225^Ac]Ac-Pr group (alone versus in combination, orange vs. red line). Co-administration resulted in faster clearance of [^177^Lu]Lu-ABD from blood (Table S1), whereas for [^225^Ac]Ac-Pr it led to significantly elevated uptake in most normal organs (Table S2). However, uptake remained < 1%ID/g in all normal organs, except kidneys. Kidney and blood AUCs increased when ^225^Ac was co-administered with ^175^Lu-ABD. Digital autoradiography showed the colocalization of [^225^Ac]Ac-Pr and [^177^Lu]Lu-ABD in SW1222 and LS174T xenografts after pretargeting with anti-GPA33 BsAb (Figure 3 and Figure S3). In the kidneys, accumulation of [^225^Ac]Ac-Pr and [^177^Lu]Lu-ABD was primarily observed in the renal cortex (Figure S4). Absorbed tumor doses were estimated based on the biodistribution results and were 0.56 Gy/MBq and 0.505 Gy/kBq for [^177^Lu]Lu-ABD and [^225^Ac]Ac-Pr, respectively. In the co-administration (cocktail) scenario, the tumor-absorbed doses were 0.30 Gy/MBq for [^177^Lu]Lu-ABD and 1.06 Gy/kBq for [^225^Ac]Ac-Pr. Complete dosimetry data are provided in Table S4.

### *In vivo* therapy

#### Study 1

**Figure 4:**
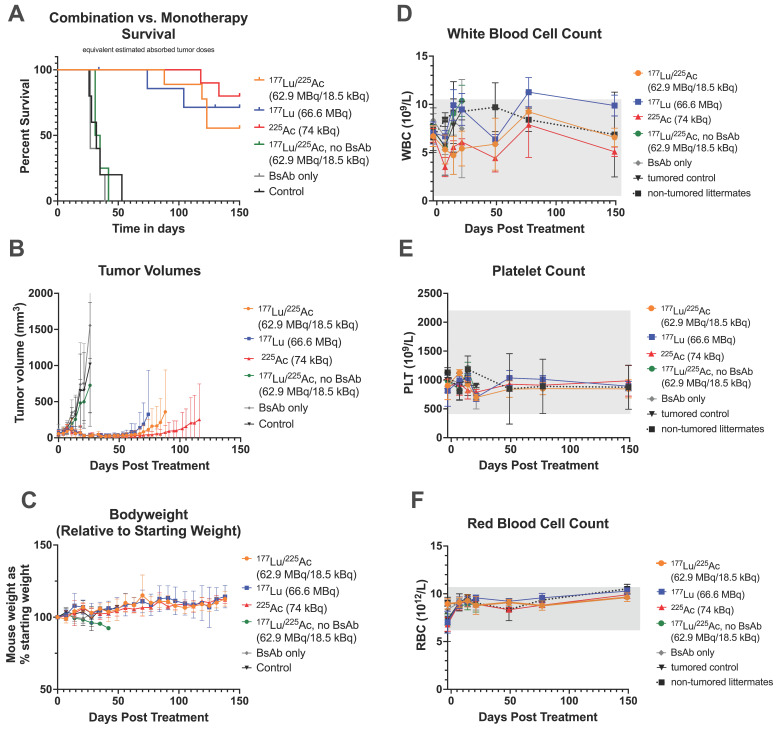
Mono-and combination therapy of GPA33(+) SW1222 xenografts at an equal tumor dose of 37-38 Gy (Study 1). Administered activities were determined based on biodistribution and dosimetry estimations. *n* = 4-10 mice/group. ^177^Lu/^225^Ac refers to the radiolabeled ligands. **A:** Survival. Censored animals in ^177^Lu-monotherapy group: one mouse was euthanized on day 35 due to weight loss (tumor volume: 63.8 mm^3^), which was considered non-treament-realted by a veterniary pathologist. A second mouse was found dead in the cage on day 130 (no tumor present). Histopathological analysis was hindered by postmortem autolysis. **B** Tumor volumes. Curves were discontinued after euthanasia of the first mouse due to tumor burden. *n* = 4-10 animals/data point. Average ± SD. **C:** Mouse weight expressed as % of pre-therapy starting weight. *n* = 4-10 animals/data point. Average ± 1SD. **D:** White blood cell count (WBC), **E:** Platelet count (PLT), **F:** Red blood cell count (RBC). **D-F:** *n* = 3-6 animals/data point. Average ± 1SD.

All treatment groups showed significantly prolonged median survival compared to controls. Median survival was not reached in any of the treatment groups (Figure 4A, Table S6). The median survival of controls ranged from 27-32 days (Table S6). Survival rates were 8/10 (5/8 histologically tumor-free) in the ^225^Ac-monotherapy group, 5/9 (4/5 histologically tumor-free) in the ^177^Lu/^225^Ac combination therapy group, and 4/8 (3/4 histologically tumor-free) in the ^177^Lu-monotherapy group. All mice were removed from the study due to tumor burden, with three exceptions: In the ^177^Lu-monotherapy group, one mouse was euthanized on day 35 due to weight loss (tumor volume: 63.8 mm^3^). Histopathological evaluation revealed minimal inflammation in both the central and peripheral nervous systems. This lesion was deemed most likely spontaneous, unrelated to experimental treatment, and of unknown cause. Another mouse in the same group was found dead in the cage on day 130 (no palpable tumor at time of death), but the severe degree of postmortem autolysis hindered histopathological interpretation of the tissues. In the combination therapy group, one mouse had severe giant cell-rich tubulointerstitial nephritis of unknown cause, which may or may not have been treatment related (Table S7).

Overall, treatment was well tolerated. Throughout the observation period, WBC, PLT, and RBC counts remained within normal limits, with no significant differences between treatment groups (Figure 4C-F). Kidney function parameters (BUN, CREA) were also not significantly different between surviving treated animals and healthy littermate controls at the study endpoint (Figure S6).

#### Study 2

**Figure 5:**
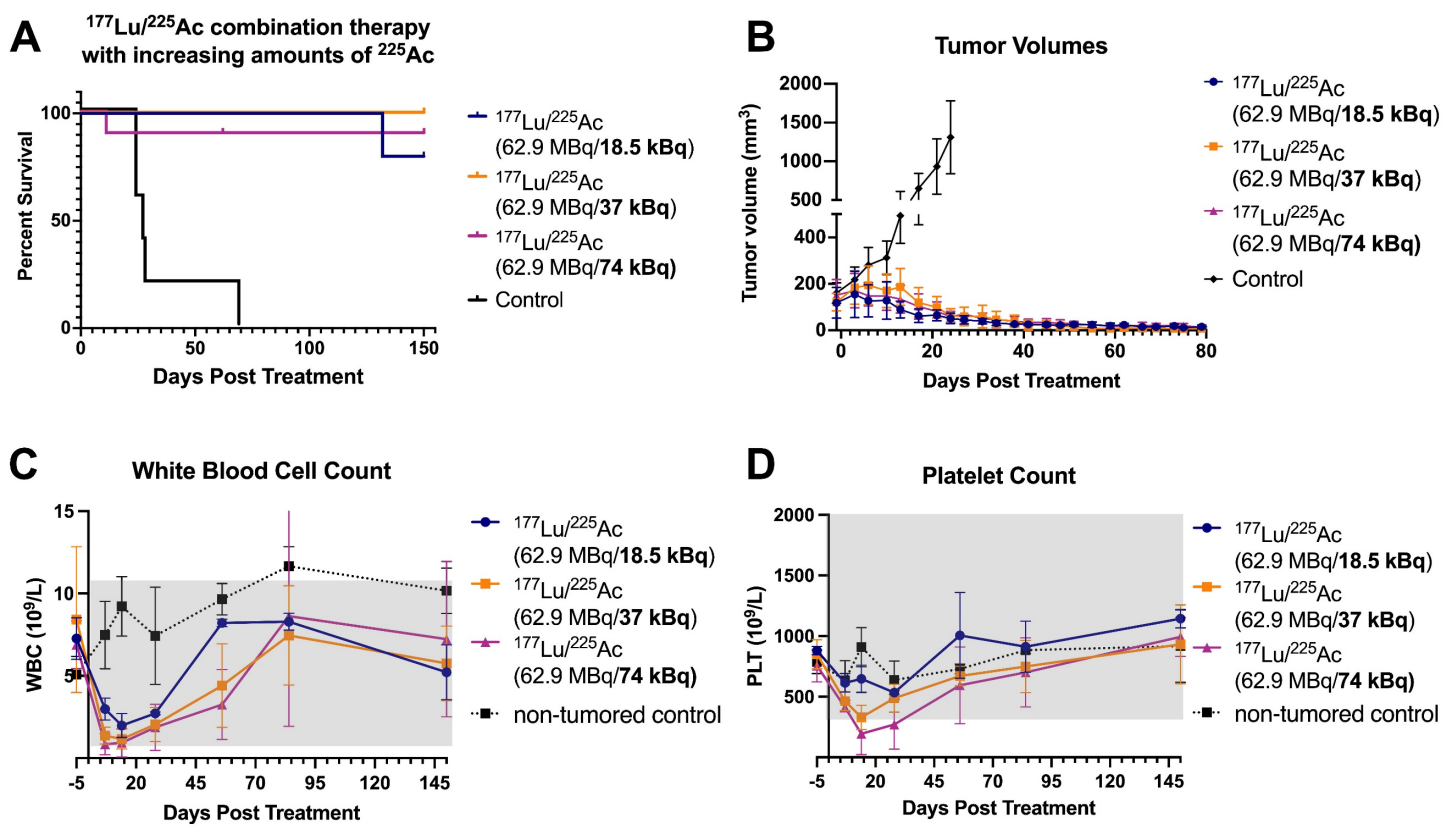
anti-GPA33 ^177^Lu/^225^Ac combination therapy in mice with established GPA33(+) SW1222 colorectal cancer xenografts (Study 2). Mice (*n* = 5-10/group) received a fixed dose of [^177^Lu]Lu-ABD (62.9 MBq) in combination with increasing doses of [^225^Ac]Ac-Pr (18.5-74 kBq) and were monitored over 150 d. **(A)** Survival. The censored animal in the ^177^Lu + 74 kBq ^225^Ac group was removed from the study due to weight loss. The animal was subsequently examined by a pathologist; however, the cause of decline could not be determined.** (B)** Tumor volume. One mouse in the ^177^Lu + 18.5 kBq ^225^Ac was euthanized because of tumor burden around in the neck area. *n* = 5-10 animals/data point. Average ± 1SD.** (C-D)** Platelet and white blood cell count. *n* = 3-4 animals/data point. Average ± 1SD.

In Study 2, mice were treated with three different combination regimens: the administered activity of [^177^Lu]Lu-ABD was kept constant at 62.9 MBq and combined with increasing amounts of [^225^Ac]Ac-Pr (18.5-74 kBq), corresponding to absorbed tumor doses of 38-97 Gy and absorbed kidney doses of 3.65-6.39 Gy. None of the treatment groups reached median survival by the study endpoint (150 d) (Figure 5). The group receiving 62.9 MBq [^177^Lu]Lu-ABD + 18.5 kBq [^225^Ac]Ac-Pr showed outcomes similar to those observed in Study 1: 4/5 mice were alive at the endpoint of 150 d post-treatment, with histological cures in 3/4 mice and one mouse with a minimal number of viable neoplastic cells surrounded by fibrosis in the tumor site (Table S6). In the group treated with 62.9 MBq [^177^Lu]Lu-ABD + 37 kBq [^225^Ac]Ac-Pr, all mice (10/10) survived to the study endpoint without any tumor remnants, as confirmed by histopathology. Histopathological evaluation also confirmed the absence of any adverse effects in this group. In the highest dose group (62.9 MBq [^177^Lu]Lu-ABD + 74 kBq [^225^Ac]Ac-Pr), 8/10 mice survived to 150 d. Early euthanasia of the two animals was required due to >20% body weight loss. Among the survivors, 6/8 showed histological cures, while 2/8 mice had a minimal number of viable neoplastic cells. Body weight and individual tumor volumes are shown in Figures S7 and S8. Histopathological analysis revealed that seven of the survivors in this highest-activity group exhibited moderate renal tubular injury, which was more pronounced than in the other treatment groups (Table S8). In most cases, this finding did not correlate with serum chemistry alterations, except in one mouse that also presented with giant cell-rich tubulointerstitial nephritis.

Median survival for mice bearing LS174T xenografts was 18 d, 33.5 d, 26.5 d and 32 d for the no treatment control, [^177^Lu]Lu-ABD monotherapy, [^225^Ac]Ac-Pr monotherapy, and [^177^Lu]Lu-ABD/[^225^Ac]Ac-Pr (62.9 MBq/37 kBq) groups, respectively. All treatment groups demonstrated significantly prolonged survival compared to controls. No significant differences in median survival were observed among the treatment groups (Figure S9).

### Toxicity studies in non-tumor-bearing mice

**Figure 6:**
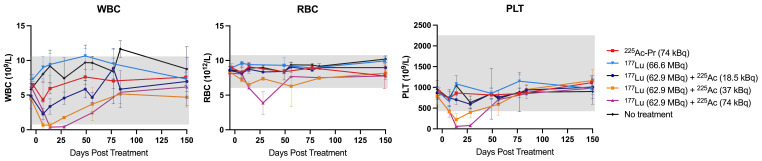
Hematology of non-tumor-bearing nude mice treated with monotherapy or combination therapy regimens. Mice received a single cycle of treatment on day 0 and were monitored for hematologic parameters through day 150 post-treatment, after which they were submitted for histopathological evaluation by a board-certified veterinary pathologist. *n* = 3-4 per data point. Average ± 1SD. Grey box indicates normal range for nude mice.

Non-tumor-bearing nude mice received the same treatment regimens as those used in Studies 1 and 2. Overall, no significant histopathological alterations were observed in mice treated with [^177^Lu]Lu-ABD (66.6 MBq) or [^225^Ac]Ac-Pr (74 kBq) monotherapy, or in the 62.9 MBq + 18.5 kBq and 62.9 MBq + 37 kBq combination groups (Table S15). A transient decrease in white blood cell and platelet counts, bordering normal limits, was observed between 7-21 d post-treatment in the two highest-activity groups (Figure 6). Two mice in the ^225^Ac-monotherapy group (74 kBq) were euthanized before the study endpoint for reasons unrelated to treatment (necrotizing vasculitis, day 70; necrotizing dermatitis, day 87).

**Figure 7:**
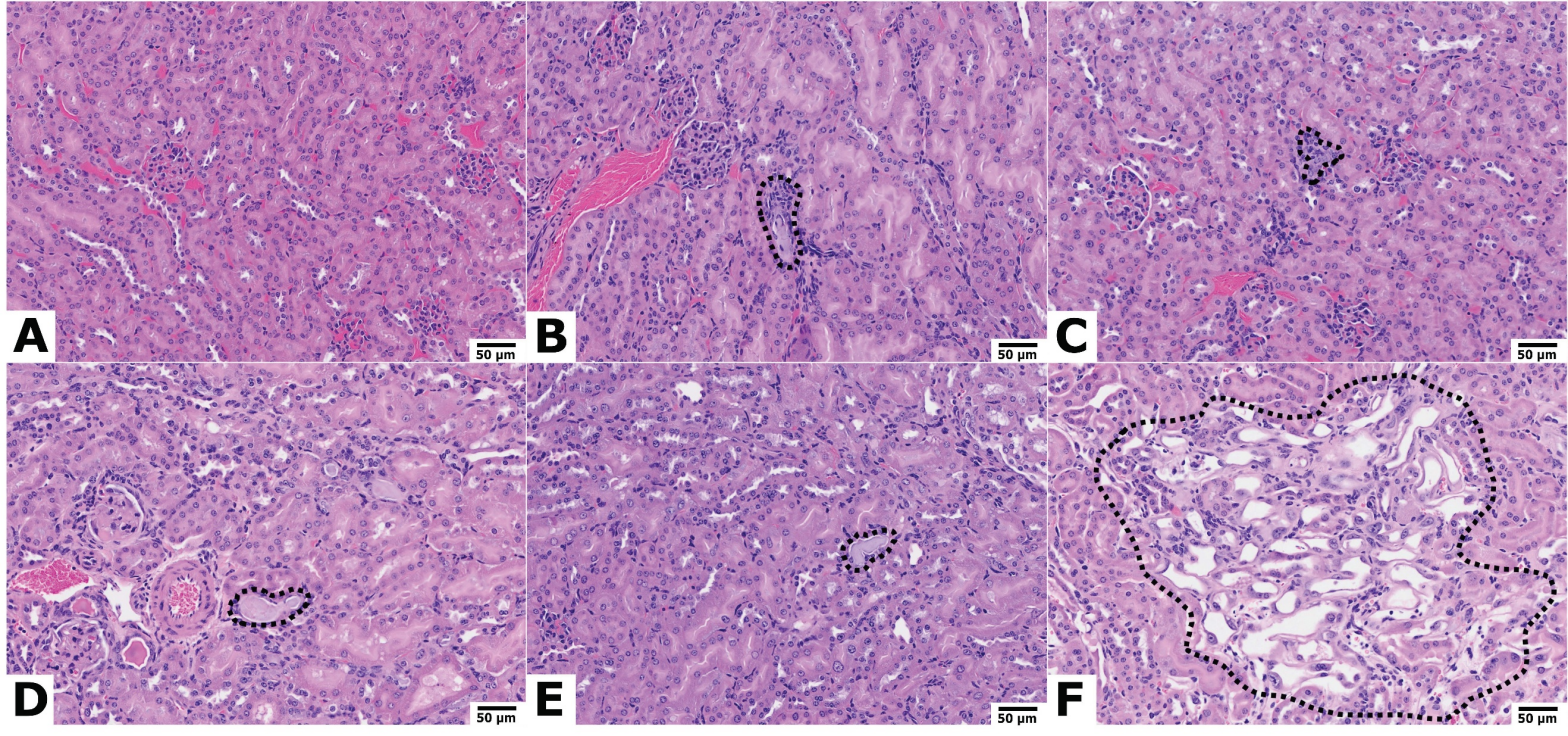
Kidney histology of mice 150 d post-treatment, hematoxylin & eosin staining, 20 x objective. Representative Images. Scale bar = 50 µm. (A) Untreated mouse: normal kidney. Treatment with (B) 66.6 MBq [^177^Lu]Lu-ABD, (C) 74 kBq [^225^Ac]Ac-Pr, (D) 62.9 MBq [^177^Lu]Lu-ABD + 18.5 kBq [^225^Ac]Ac-Pr, and (E) 62.9 MBq [^177^Lu]Lu-ABD + 37 kBq [^225^Ac]Ac-Pr: Minimal tubular degeneration (dashed areas). (F) Treatment with 62.9 MBq [^177^Lu]Lu-ABD + 74 kBq [^225^Ac]Ac-Pr: tubular degeneration (dashed area) with epithelial cell attenuation, loss of brush borders, single-cell necrosis, and karyomegaly.

At study endpoint, no renal toxicity was observed in the combination groups receiving 62.9 MBq [^177^Lu]Lu-ABD + 18.5 kBq or + 37 kBq [^225^Ac]Ac-Pr. Average renal function parameters in these groups were comparable to those of untreated control mice (Figure S10), with one exception: a single mouse in the 62.9 MBq + 37 kBq combination group exhibited severe giant cell-rich tubulointerstitial nephritis. Similar findings were observed in one treated tumor bearing mouse from each of the 62.9 MBq + 18.5 kBq and 62.9 MBq +74 kBq groups. While these findings were unusual, no definitive conclusions could be drawn at this stage. Retrospective analyses across larger study groups will be required to further evaluate incidence. Most notably, 3/5 mice in the highest-activity group (62.9 MBq [^177^Lu]Lu-ABD + 74 kBq [^225^Ac]Ac-Pr) were euthanized before study endpoint due to significant weight loss. Histopathological analysis revealed severe bone marrow depletion in all three cases, which was deemed treatment-related. The remaining mice in this group, euthanized at the study endpoint, exhibited a moderate degree of renal tubular injury, also considered treatment-related. Representative kidney images are provided in Figure 7. Scoring of kidney damage in surviving tumor-bearing and non-tumor bearing mice is detailed in the Supplement (Tables S9-S14).

### Immunofluorescence staining, DNA damage and apoptosis qPCR array

Analysis of SW1222 xenograft sections 24 h post-treatment showed increased levels of p-H2Ax positive cells compared to non-treated control samples (Figure S11 and S12), with no significant differences observed between monotherapy and combination therapy groups.

For the qPCR, 23/86 genes from the DNA damage panel and 30/86 genes from the apoptosis panel were included in the final analysis (Figure S13). Remaining genes were excluded due to undetermined C_T_ values, or inconsistencies between housekeeping genes. Most genes analyzed from the apoptosis panel showed upregulation. *TNFRF10B, XIAP*, and *BNIP2* were downregulated in the combination but not in the monotherapy groups. *CD70* was downregulated following ^225^Ac treatment but upregulated in the other groups. In the DNA damage panel, gene expression changes showed a mixed pattern of up- and downregulation, with some treatment-specific differences. *BLM* and *GADD45G* were upregulated only following treatment with ^177^Lu. *LIG1 and XPC* were upregulated in both monotherapy groups, but not the combination group. Conversely, *CHEK2, MLH3* and* PPP1R15A* were upregulated in the combination therapy group and downregulated in the monotherapy groups. *PARP1*, *PRKDC, DDB1* and* CRY1* were exclusively upregulated in the ^225^Ac group.

## Discussion

To the best of our knowledge, this is the first study to report on dual-isotope cocktail radiotherapy using PRIT. We demonstrate the feasibility and therapeutic potential of a ^177^Lu/^225^Ac combination PRIT approach in CRC models. Furthermore, we investigated the dose-toxicity relationship by integrating detailed histopathological assessments, quantitative dosimetry, and pilot studies of radiobiological responses to combined ^177^Lu/^225^Ac-PRIT.

We first verified the feasibility of a combination DOTA-PRIT approach through biodistribution and autoradiography studies. To simulate the conditions of radioligand cocktailing, we co-administered mixtures of radiolabeled and non-radiolabeled ligands. Importantly, a constant total ligand dose of 700 pmol was maintained across all groups to ensure comparable *in vivo* PRIT performance. Cocktailing did not adversely affect the overall ability to target the tumor with both radioligands and just slightly affected the biodistribution of either radioligand. We observed a trend toward increased tumor uptake and retention of [^225^Ac]Ac-Pr when co-injected with ^175^Lu-ABD, which translated into a considerable difference in dosimetry estimations. The mechanism underlying this phenomenon remains subject to speculation. While structurally slightly different, both ligands use the same “affinity handle” (Lu-DOTA) to bind to the C825. The [^225^Ac]Ac-DOTA complex has not shown affinity for the C825 scFv 11 and is thus unlikely to contribute to the interaction with the BsAb. Our experiments indicate slower blood kinetics of [^225^Ac]Ac-Pr compared to [^177^Lu]Lu-ABD when administered alone, which may be attributed to their slight structural differences. The higher concentration of [^225^Ac]Ac-Pr in blood may results in a stochiometric advantage for [^225^Ac]Ac-Pr uptake in the tumor. Further investigations into these matters are needed but are outside the scope of this study. iQID digital autoradiography confirmed successful co-targeting of both GPA33-pretargeted radioligands in GPA33(+) SW1222 and LS174T CRC xenografts. Nevertheless, the assumption of unaltered biodistribution during co-injection may not always hold true and should be carefully validated—especially when cocktailing strategies are used for dosimetry purposes.

We used biodistribution data to estimate the absorbed tumor doses in both the monotherapy and combination therapy settings and to determine the injected activities for our first therapy study (Study 1). At an estimated absorbed dose to the tumor of 37-38 Gy, combination therapy demonstrated efficacy comparable to monotherapy. A similar observation was reported by Meyer *et al.*, who studied ^177^Lu/^225^Ac tandem therapy using PSMA-617 in a disseminated prostate cancer model, with injected activities of 35 MBq ^177^Lu and 40 kBq ^225^Ac 12. Another preclinical study exploring ^177^Lu/^225^Ac-PSMA-617 tandem therapy (36 kBq ^225^Ac + 37 MBq ^177^Lu) indicated superior effects of combination treatment compared to monotherapy after 8 weeks of observation and imaging with [^18^F]F-AlF-PSMA11 17. However, the short-term follow-up and lack of dosimetry make more in-depth comparisons difficult.

In the SW1222 model, histological cures were achieved across all treatment groups. A follow-up study (Study 2) demonstrated that treatment with up to 62.9 MBq of [^177^Lu]Lu-ABD combined with 37 kBq of [^225^Ac]Ac-Pr was well tolerated and resulted in 100% histological cures in the SW1222 model. Due to the extremely limited availability of data on ^177^Lu/^225^Ac combination therapy, dose setting is challenging. With study 2 we sought to further investigate both the dose-effect and dose-toxicity relationships. Although future studies should explore additional treatment regimens, our current data still provided meaningful insights into these relationships and will serve as a valuable reference point for future investigations by our group and others. This also highlights that the tumor model plays an important role in the dose-effect relationship and must be carefully considered when designing future studies. We applied the most successful treatment regimen from our SW1222 studies to a second tumor model, LS174T, which is known to be a more heterogeneous and aggressive 18, a characteristic also reflected in our autoradiography images (Figure S3). Although all treatments produced significant therapeutic effects, efficacy in the LS174T model was reduced compared to SW1222. Nevertheless, the median survival achieved with mono-and combination treatments was comparable to previously reported studies with ^225^Ac and ^213^Bi, or ^177^Lu 19, 20.

The absence of improved efficacy from the ⍺/β dual isotope combination compared to mono-isotope regimens at equivalent tumor radiation doses was somewhat surprising. One potential explanation may lie in the choice of tumor model. While cell line-derived subcutaneous xenografts provide a logical starting point for evaluating the feasibility, efficacy, and toxicity of dual-isotope DOTA-PRIT, they do not necessarily mimic the disease burden of patients with multiple tumor sites of varying sizes, who would likely be good candidates for combination therapy in a clinical setting. Follow-up studies in more representative models, such as freshly passaged patient-derived xenografts and disseminated intraperitoneal tumor models with varying tumor burdens, will aim to further explore the relationship between ⍺/β dose ratios, efficacy, and disease volume and distribution.

This study represents one of the first preclinical investigations into the dose-toxicity dynamics of ^177^Lu/^225^Ac co-treatment. Dual-isotope DOTA-PRIT was well tolerated as monotherapy and remained safe at combined regimens up to 62.9 MBq of [^177^Lu]Lu-ABD + 37 kBq of [^225^Ac]Ac-Pr, corresponding to absorbed doses of 58.1 Gy to the tumor, 4.62 Gy to the kidneys, and 2.31 Gy to blood. However, at the highest administered activities—62.9 MBq of [^177^Lu]Lu-ABD combined with 74 kBq of [^225^Ac]Ac-Pr (6.47 Gy to the kidneys, 3.05 Gy to blood)—we observed increased kidney damage among survivors and severe bone marrow depletion, indicating that this regimen approaches the maximum tolerated dose in mice. Notably, these administered activities fall within or below previously established safe individual limits for ^177^Lu and ^225^Ac-DOTA-PRIT 10, 11, but they establish preliminary thresholds approaching the maximum tolerated dose for their combined use.

Comparable findings have been observed clinically. During tandem radioligand therapy with ^177^Lu/^225^Ac-PSMA-617, pilot studies have reported occasional cases of hematological toxicity during treatment with 44-60 kBq/kg of ^225^Ac and an average of 6 GBq ^177^Lu 7, 21. In a recent case study, a patient with pancreatic neuroendocrine neoplasm received a cumulative dose of 11.3 GBq [^177^Lu]LuDOTA-LM3 plus 26.4 MBq [^225^Ac]AcDOTA-LM3 22. Although not directly comparable, the administered activities of ^225^Ac and ^177^Lu per bodyweight in our preclinical study were considerably higher. This, in part, can be attributed to the excellent TIs provided by the pretargeting approach. Ongoing clinical efforts, such as the AlphaBet trial (NCT05383079), are systematically investigating the dose-limiting toxicities and therapeutic efficacy of combined ⍺/β therapies in metastatic prostate cancer. In this study, patients are treated with a fixed dose of [^177^Lu]Lu-PSMA-I&T (7.4 MBq) in combination with escalating doses of ^223^Ra 23. Our findings emphasize the need for similarly rigorous preclinical and clinical evaluations, particularly those that consider not only the total absorbed doses to critical organs, but also the biological interplay and cumulative effects of combined α and β emissions. These insights will be essential for the successful clinical translation of combination radionuclide therapies.

The ratio between alphas and betas is a fascinating topic and may prove to be a critical determinant of therapeutic success. Given their distinct radiobiological properties, a dual-isotope combination approach is theoretically best suited for patients with advanced disease involving multiple tumors of varying sizes. In the few clinical studies conducted to date, the administered activities and ^225^Ac/^177^Lu ratios have been variable and determined on a patient-to-patient basis, influenced by patient condition, prior therapies, and total tumor burden 21. Our preclinical data support the need for a more individualized approach going forward. Notably, autoradiography in our study revealed a heterogenous intratumoral activity distribution pattern in one of the models, leading us to speculate that a higher dose of ^177^Lu—owing to its longer tissue penetration—might have improved efficacy. However, future studies must systematically investigate the relationship between injected activities, tumor size distribution, antigen density, heterogeneity, and therapeutic benefit. Such investigations will help define the foundational principles to guide clinical decision-making and optimize treatment strategies in multi-isotope radionuclide therapy.

We performed an exploratory qPCR gene expression analysis to investigate potential differences in DNA damage response and apoptosis in tumors following treatment with ^225^Ac, ^177^Lu, or their combination. Most analyzed apoptosis markers were upregulated across all treatment groups, consistent with the excellent therapeutic responses observed *in vivo*. In contrast, the DNA damage panel revealed differences in DNA damage response between monotherapy and combination therapy, as well as between the two radionuclides, suggesting distinct underlying mechanisms of action. This analysis was performed at a single time point (24 h post-treatment), which limits insight into the temporal dynamics of gene expression changes. Evaluating multiple time points may reveal important temporal patterns in treatment-induced responses and potentially guide synergistic combination treatment regimens. While the current findings are intriguing, the limited sample size and single time point restrict our ability to draw definitive conclusions, and a detailed analyses of these differences falls outside the scope of this study. Nevertheless, these preliminary results may serve as a starting point for more in-depth studies we plan to conduct in the future.

Using a pretargeting approach for isotope cocktailing, as demonstrated in this study, may offer significant advantages. These include the general advantages of pretargeting, namely reduced overall toxicity due to rapid clearance on non-tumor-bound radioligand and the potential for high TIs 24. In addition, the approach is highly versatile and not confounded by significant differences in pharmacokinetics or radioligand affinity. The DOTA-PRIT platform permits easy exchange and combination of a wide range of radionuclides. Because the same affinity handle is used across radioisotopes, the Proteus ligand can be radiolabeled with various radionuclides without affecting antigen engagement or altering the interaction between antibody and radioligand 11. Prior studies, as well recent clinical efforts, have demonstrated the therapeutic benefit of combining long- and short-range β-emitters (^90^Y/^177^Lu) for treatment of neuroendocrine tumors 25-27. Depending on the internalizing properties of the targeting vector, Auger emitters may also provide an excellent radiobiological complement to β- and ⍺-emitters in dual or even triple therapy combinations. Another key application is theranostic cocktailing, for example the ^134^Ce/^225^Ac pair 28 for PET diagnostics and targeted ⍺-therapy. Exploring other isotope cocktails using our DOTA-PRIT approach is therefore an appealing avenue for future research. Furthermore, our group recently reported on a novel self-assembling and disassembling antibody (SADA) platform, which could simplify dosing regimens and potentially further improve TIs and maximum deliverable tumor dose 29.

## Conclusion

We demonstrated the feasibility and therapeutic efficacy of PRIT using a ^177^Lu/^225^Ac cocktail. Moreover, this study represents one of the first systematic preclinical evaluations of dose-toxicity relationships for dual-isotope therapies. Collectively, these findings should inform future exploration of isotope cocktailing therapies.

## Supplementary Material

Supplementary figures and tables.

## Data Availability

The datasets generated and/or analyzed during the current study are available from the corresponding author upon reasonable request.

## Abbreviations

ABD: Aminobenzyl-DOTA
AUC: Area Under the Curve
BsAb: Bispecific Antibody
BUN: Blood Urea Nitrogen
CBC: Complete Blood Count
CREA: Creatinine
CRC: Colorectal Cancer
FBS: Fetal Bovine Serum
iQID: ionizing-radiation quantum imaging detector
PBS: Phosphate buffered Saline
PLT: Platelets
PRIT: Pretargeted Radioimmunotherapy
RBE: Relative Biological Effectiveness
RBC: Red Blood Cell Count
SADA: Self Assembling and DisAssembling Antibody
Tis: Therapeutic Indices
WBC: White Blood Cell Count
