# Supplementary material for: Pretargeted 177Lu/225Ac combination therapy of colorectal cancer
Source: Theranostics. 2026 Apr 8;16(11):5898–910. doi: 10.7150/thno.126399 (PMC13141694; doi:10.7150/thno.126399)
Supplement: Supplementary file 1 — Supplementary figures and tables. [file thnov16p5898s1.pdf]

Supplementary Material

Pretargeted ^{177}Lu/^{225}Ac combination therapy of colorectal cancer

Material and Methods

	1	2	3	4	5	6	7	8	9	10	11	12
A	ABL1	APEX1	ATM	ATR	ATRIP	ATRX	BARD1	BAX	BBC3	BLM	BRCA1	BRIP1
B	CDC25A	CDC25C	CDK7	CDKN1A	CHEK1	CHEK2	CIB1	CRY1	CSNK2A2	DDB1	DDB2	DDIT3
C	ERCC1	ERCC2	EXO1	FANCA	FANCD2	FANCG	FEN1	GADD45A	GADD45G	H2AFX	HUS1	LIG1
D	MAPK12	MBD4	MCPH1	MDC1	MLH1	MLH3	MPG	MRE11A	MSH2	MSH3	NBN	NTHL1
E	OGG1	PARP1	PCNA	PMS1	PMS2	PNKP	PPM1D	PPP1R15A	PRKDC	RAD1	RAD17	RAD18
F	RAD21	RAD50	RAD51	RAD51B	RAD9A	RBBP8	REV1	RNF168	RNF8	RPA1	SIRT1	SMC1A
G	SUMO1	TOPBP1	TP53	TP53BP1	TP73	UNG	XPA	XPC	XRCC1	XRCC2	XRCC3	XRCC6
H	ACTB	B2M	GAPDH	HPRT1	RPLP0	HGDC	RTC	RTC	RTC	PPC	PPC	PPC

Figure S1: Genes included in the Human DNA Damage Signaling Pathway RT^2 Profiler PCR Array (PAHS-029ZA12, Qiagen) PPC: Positive PCR control.

	1	2	3	4	5	6	7	8	9	10	11	12
A	ABL1	AIFM1	AKT1	APAF1	BAD	BAG1	BAG3	BAK1	BAX	BCL10	BCL2	BCL2A1
B	BCL2L1	BCL2L10	BCL2L11	BCL2L2	BFAR	BID	BIK	BIRC2	BIRC3	BIRC5	BIRC6	BNIP2
C	BNIP3	BNIP3L	BRAF	CASP1	CASP10	CASP14	CASP2	CASP3	CASP4	CASP5	CASP6	CASP7
D	CASP8	CASP9	CD27	CD40	CD40LG	CD70	CFLAR	CIDEA	CIDEB	CRADD	CYCS	DAPK1
E	DFFA	DIABLO	FADD	FAS	FASLG	GADD45A	HRK	IGF1R	IL10	LTA	LTBR	MCL1
F	NAIP	NFKB1	NOD1	NOL3	PYCARD	RIPK2	TNF	TNFRSF10A	TNFRSF10B	TNFRSF11B	TNFRSF1A	TNFRSF1B
G	TNFRSF21	TNFRSF25	TNFRSF9	TNFSF10	TNFSF8	TP53	TP53BP2	TP73	TRADD	TRAF2	TRAF3	XIAP
H	ACTB	B2M	GAPDH	HPRT1	RPLP0	HGDC	RTC	RTC	RTC	PPC	PPC	PPC

Figure S2: Genes included in the Human Apoptosis RT^2 profiler PCR Array (PAHS-012ZA-12, Qiagen) PPC: Positive PCR control.

Pathology

Mice were euthanized with carbon dioxide overdose. Blood for complete blood count and serum chemistry analysis was collected via cardiac puncture. Selected organs were examined macroscopically and thereafter fixed in 10% neutral buffered formalin for 72 hours, followed by decalcification of bones in a formic acid solution (Surgipath Decalcifier I, Leica Biosystems, Nussloch, Germany). Formalin-fixed tissues were processed in ethanol and xylene and embedded in paraffin using the Leica ASP6025 tissue processor). Tissues were sectioned at 5 microns and stained with hematoxylin and eosin (H&E). The following tissues were examined: heart, thymus, lungs, liver, gallbladder, kidneys, pancreas, stomach,

duodenum, jejunum, ileum, cecum, submandibular and mesenteric lymph nodes, salivary glands, skin, urinary bladder, uterus, cervix, vagina, ovaries, oviducts, adrenal glands, spleen, thyroid gland, esophagus, trachea, spinal cord, vertebrae, sternum, femur, tibia, stifle joint, skeletal muscle, nerves, skull, nasal cavity, oral cavity, teeth ears, eyes, pituitary gland, brain, and additional lesions if present, e.g. xenografts.

Multiplex IF staining

Automated multiplex IF staining was conducted using Leica Bond BX staining system. Paraffin-embedded tissues were sectioned at 5 µm and baked at 58 °C for 1 h. Slides were loaded in Leica Bond and IF staining was performed as follows. Samples were dewaxed and pretreated with EDTA-based epitope retrieval ER2 solution (Leica, AR9640) for 20 min at 100 °C. The 3-plex antibody staining and detection were conducted sequentially. The primary antibodies against Ki67 (0.04 ug/ml, rb, Cell signaling technology, 9027), Cl-caspase3/A488 (0.025 ug/ml, rb, Cell Signaling Technologies, 9667) and p-H2AX /C430 (0.025 µg/ml, rb, Abcam, ab11174) were incubated for 1h at RT followed by incubation with Leica Bond Polymer anti-rabbit HRP secondary antibody for 8 minutes. After that, CF® dye tyramide conjugate (Biotium, 92174) or Alexa Fluor tyramide signal amplification reagents (Life Technologies, B40953, B40958) were used for signal detection. After each round of IF staining, Epitope retrieval was performed for denaturization of primary and secondary antibodies before another primary antibody was applied. After the run was finished, slides were washed in PBS and incubated in 5 µg/ml 4',6-diamidino-2-phenylindole (DAPI) (Sigma Aldrich) in PBS for 5 min, rinsed in PBS, and mounted in Mowiol 4–88 (Calbiochem). Slides were kept overnight at -20 °C before imaging.
Slides were scanned on a Pannoramic Scanner (3DHistech, Budapest, Hungary) using a 20x/0.8NA objective. Images were analyzed using QuPath v0.4.4 for MacOS using the DAPI channel from cell detection and creating a classifier to determine the p-H2AX positive cells. For each tumor sample five areas were selected at random and analyzed.

Results

Biodistribution

Table S1: Biodistribution of [^{177}Lu]Lu-ABD alone or in combination with ^{139}La-Pr after pretargeting with anti-GPA33 BsAb. Mice (*n* = 4-5/group) were administered with either 700 pmol [^{177}Lu]Lu-ABD alone or a cocktail of 350 pmol [^{177}Lu]Lu-ABD and 350 pmol of ^{139}La-Pr. Data presented as average %ID/g ± SD.

**%ID/g**	**2 hours**		**24 hours**		**48 hours**		**120 hours**	
	**[^{177}Lu]Lu-ABD**	**[^{177}Lu]Lu-ABD + ^{139}La-Pr**	**[^{177}Lu]Lu-ABD**	**[^{177}Lu]Lu-ABD + ^{139}La-Pr**	**[^{177}Lu]Lu-ABD**	**[^{177}Lu]Lu-ABD + ^{139}La-Pr**	**[^{177}Lu]Lu-ABD**	**[^{177}Lu]Lu-ABD + ^{139}La-Pr**
**Blood**	0.162 ± 0.01*	0.056 ± 0.006	0.041 ± 0.009*	0.013 ± 0.002	0.016 ± 0.004*	0.006 ± 0.001	0.002 ± 0.001	0.0020 ± 0.0005
**Tumor**	11± 6	9 ± 3	7 ± 3*	2.4 ± 0.9	5 ± 2*	1.7 ± 0.5	1.7 ± 1.0	1.0 ± 0.3
**Heart**	0.13 ± 0.04*	0.045 ± 0.006	0.049 ± 0.005*	0.019 ± 0.003	0.04 ± 0.04	0.013 ± 0.002	0.019 ± 0.007*	0.008 ± 0.003
**Lungs**	0.2 ± 0.1	0.09 ± 0.02	0.04 ± 0.01	0.02 ±0.01	0.04 ± 0.02*	0.016 ± 0.005	0.016 ± 0.009*	0.007 ± 0.002
**Liver**	0.4 ± 0.2	0.4 ± 0.1	0.07 ± 0.02	0.05 ± 0.02	0.05 ± 0.02	0.03 ± 0.02	0.04 ± 0.02*	0.019 ± 0.009
**Spleen**	0.12 ± 0.04	0.07 ± 0.02	0.07 ± 0.02	0.050 ± 0.009	0.06 ± 0.01*	0.030 ± 0.007	0.05 ± 0.01*	0.028 ± 0.006
**Stomach**	0.07 ± 0.04	0.04 ± 0.03	0.03 ± 0.01	0.02 ± 0.01	0.013 ± 0.004	0.014 ± 0.006	0.009 ± 0.004	0.007 ± 0.006
**Small Intestine**	0.6 ± 0.4	0.8 ± 1.0	0.03 ± 0.01*	0.016 ± 0.003	0.027 ± 0.009*	0.011 ± 0.004	0.014 ± 0.003*	0.004 ± 0.001
**Large Intestine**	0.6 ± 0.4	0.7 ± 0.07	0.05 ± 0.04	0.04 ± 0.03	0.03 ± 0.01	0.04 ± 0.02	0.016 ± 0.009	0.02 ± 0.02
**Kidney**	0.6 ± 0.4	0.8 ± 0.2	0.50 ± 0.07	0.54 ± 0.09	0.29 ± 0.08	0.4 ± 0.1	0.17 ± 0.05	0.14 ± 0.03
**Muscle**	0.2 ± 0.3	0.03 ± 0.01	0.010 ± 0.005	0.011 ± 0.006	0.008 ± 0.005	0.008 ± 0.003	0.005	0.003 ± 0.001
**Bone**	0.08 ± 0.06	0.10 ± 0.10	0.016 ± 0.006	0.04 ± 0.03	0.021 ± 0.004	0.06 ± 0.04	0.014 ± 0.007	0.04 ± 0.03

* Statistically significant difference ($p < 0.05$) between [^{177}Lu]Lu-ABD and [^{177}Lu]Lu-ABD + ^{nat}La-Pr using students t-test.

Table S2: Biodistribution of [^{225}Ac]Ac-Pr alone or in combination with ^{175}Lu-ABD after pretargeting with anti-GPA33 BsAb. Mice (n=4-5/group) were administered with either 700 pmol [^{225}Ac]Ac-Pr alone or a cocktail of 350 pmol [^{225}Ac]Ac-Pr and 350 pmol of ^{175}Lu-ABD. Data presented as average %ID/g ± SD.

%ID/g	2 hours		24 hours		48 hours		120 hours	
	[^{225}Ac]Ac-Pr	[^{225}Ac]Ac-Pr + ^{175}Lu-ABD	[^{225}Ac]Ac-Pr	[^{225}Ac]Ac-Pr + ^{175}Lu-ABD	[^{225}Ac]Ac-Pr	[^{225}Ac]Ac-Pr + ^{175}Lu-ABD	[^{225}Ac]Ac-Pr	[^{225}Ac]Ac-Pr + ^{175}Lu-ABD
**Blood**	0.4 ± 0.1*	1.8 ± 0.2	0.14 ± 0.03*	0.5 ± 0.1	0.04 ± 0.02*	0.17 ± 0.09	0.006 ± 0.003*	0.05 ± 0.02
**Tumor**	13 ± 6*	23 ± 8	11 ± 5	21 ± 7	11 ± 9	15 ± 8	8 ± 3*	19 ± 8
**Heart**	0.33 ± 0.10*	1.2 ± 0.2	0.14 ± 0.04*	0.7 ± 0.2	0.08 ± 0.02*	0.5 ± 0.3	0.06 ± 0.02*	0.5 ± 0.2
**Lungs**	0.4 ± 0.1*	1.2 ± 0.4	0.19 ± 0.10	0.6 ± 0.4	0.13 ± 0.08*	0.4 ± 0.1	0.06 ± 0.03*	0.4 ± 0.2
**Liver**	0.3 ± 0.1*	0.8 ± 0.3	0.4 ± 0.1*	0.9 ± 0.2	0.50 ± 0.08	0.8 ± 0.3	0.32 ± 0.06*	1.5 ± 0.7
**Spleen**	0.22 ± 0.09*	1.0 ± 0.2	0.12 ± 0.06*	1.0 ± 0.3	0.2 ± 0.1*	0.8 ± 0.3	0.18 ± 0.05*	1.7 ± 0.9
**Stomach**	0.21 ± 0.06*	0.6 ± 0.2	0.06 ± 0.03*	0.28 ± 0.08	0.07 ± 0.03	0.2 ± 0.2	0.04 ± 0.02*	0.17 ± 0.09
**Small Intestine**	0.15 ± 0.04*	0.7 ± 0.1	0.09 ± 0.03*	0.5 ± 0.2	0.06 ± 0.02	0.5 ± 0.5	0.05 ± 0.01*	0.28 ± 0.07
**Large Intestine**	0.13 ± 0.04*	0.4 ± 0.2	0.08 ± 0.03*	0.24 ± 0.07	0.2 ± 0.2	0.2 ± 0.1	0.07 ± 0.07	0.3 ± 0.2
**Kidney**	1.1 ± 0.3	2.0 ± 0.9	0.6 ± 0.1	1.3 ± 0.5	0.7 ± 0.2*	1.1 ± 0.2	0.31 ± 0.06	0.8 ± 0.4
**Muscle**	0.06 ± 0.03*	0.30 ± 0.09	0.028 ± 0.008	0.09 ± 0.05	0.04 ± 0.02*	0.11 ± 0.04	0.05 ± 0.06*	0.15 ± 0.03
**Bone**	0.3 ± 0.2*	0.9 ± 0.2	0.13 ± 0.05	0.2 ± 0.1	0.2 ± 0.1	0.14 ± 0.09	0.4 ± 0.2	1.1 ± 0.5

*Statistically significant difference between [^{225}Ac]Ac-Pr and [^{225}Ac]Ac-Pr + ^{nat}Lu-ABD using students t-test.

Table S3: Absolute AUC values for tumor, blood and kidney from the biodistribution of [^{225}Ac]Ac-Pr and [^{177}Lu]Lu-ABD alone or in combination. Biodistribution experiments were performed in nude mice bearing GPA33(+) colorectal cancer xenografts according to the DOTA-PRIT regimen. AUC analysis was performed in GraphPad Prism. Average ± 1SEM.

AUC	Tumor	Blood	Kidney
**[^{177}Lu]Lu-ABD**	569 ± 112^{a}	3.7 ± 0.3^{a}	39 ± 6^{a}
**[^{177}Lu]Lu-ABD + ^{139}La-Pr**	289 ± 47^{b}	1.32 ±0.09^{b}	44 ± 5^{b}
**[^{225}Ac]Ac-Pr**	1248 ± 367	10 ± 1^{c}	75 ± 7^{c}
**[^{225}Ac]Ac-Pr + ^{175}Lu-ABD**	2210 ± 430^{a,b}	42 ± 5^{a,b,c}	132 ± 20^{a,b,c}

significant difference($p < 0.05$) between ^{a}[^{177}Lu]Lu-ABD vs [^{225}Ac]Ac-Pr + ^{175}Lu-ABD, ^{b} [^{177}Lu]Lu-ABD + ^{139}La-Pr vs [^{225}Ac]Ac-Pr + ^{175}Lu-ABD, ^{c} [^{225}Ac]Ac-Pr and [^{225}Ac]Ac-Pr + ^{175}Lu-ABD, tested with one-way ANOVA with post-hoc t-test adjusted for multiple comparisons. No significant differences between [^{177}Lu]Lu-ABD and [^{177}Lu]Lu-ABD + ^{139}La-Pr.

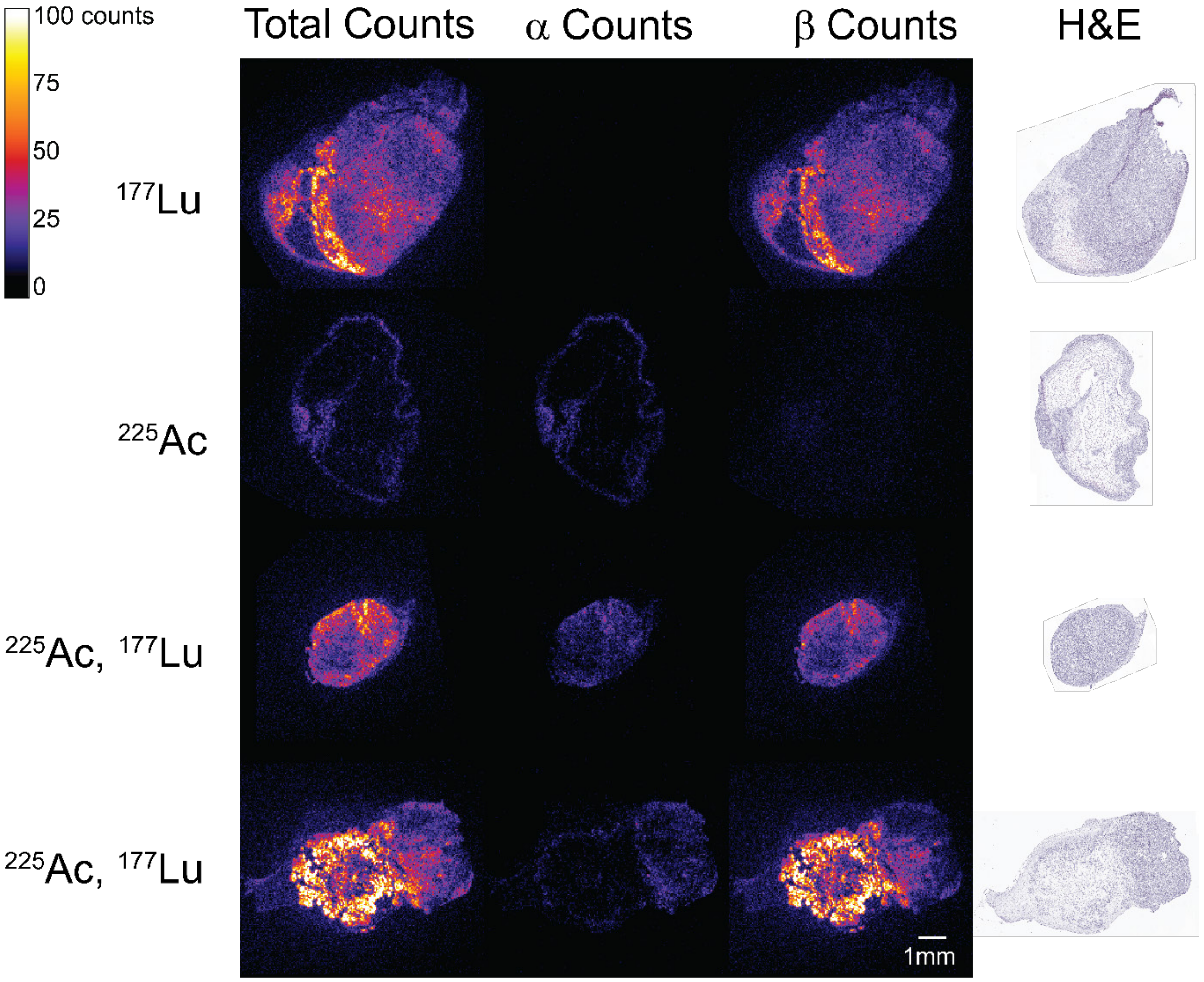

Figure S3: Digital autoradiography of LS174T xenografts pretargeted with anti-GPA33 BsAb 24 h after injection of [^{225}Ac]Ac-Pr and [^{nat}Lu]Lu-ABD using iQID. Mice were administered with either 700 pmol [^{177}Lu]Lu-ABD (1.85 MBq) and [^{225}Ac]Ac-Pr (37 kBq) alone or a cocktail of 350 pmol (37

kBq) [^{225}Ac]Ac-Pr and 350 pmol (1.85 MBq) [^{177}Lu]Lu-ABD. First column shows combined signal from ^{177}Lu and ^{225}Ac, second column ^{225}Ac signal and third column ^{177}Lu signal only. H&E staining of consecutive tissue sections are shown in the last column.

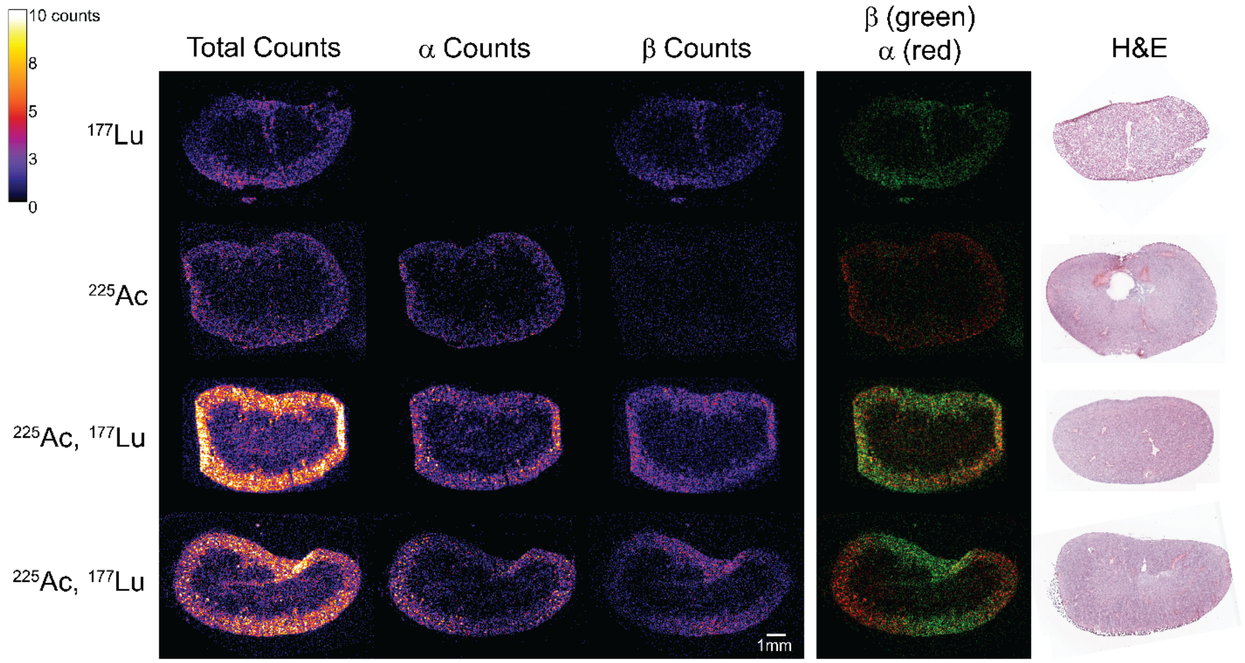

Figure S4: Digital autoradiography of kidneys harvested from mice with SW1222 xenografts 24 hours after injection of antiGPA33 BsAb + of [^{225}Ac]Ac-Pr and [^{177}Lu]Lu-ABD using iQID. Mice were administered with either 700 pmol [^{177}Lu]Lu-ABD (1.85 MBq) and [^{225}Ac]Ac-Pr (37 kBq) alone or a cocktail of 350 pmol (37 kBq) [^{225}Ac]Ac-Pr and 350 pmol (1.85 MBq) [^{177}Lu]Lu-ABD. First column shows combined signal from ^{177}Lu and ^{225}Ac, second column ^{225}Ac signal and third column ^{177}Lu signal only. Fourth column shows an overlay of ^{225}Ac (red) and ^{177}Lu (green). H&E staining of consecutive tissue sections are shown in the last column.

Table S4: Tissue dosimetry estimations based on biodistribution in SW1222 xenograft bearing mice. For all emissions, RBE =1.

Organ	Absorbed dose (Gy/kBq)			
	[^{177}Lu]Lu-ABD	[^{177}Lu]Lu-ABD + ^{139}La-Pr	[^{225}Ac]Ac-Pr	[^{225}Ac]Ac-Pr + ^{175}Lu-ABD
**Blood**	0.000057	0.000025	0.012	0.020
**Tumor**	0.00056	0.00030	0.51	1.06
**Heart**	0.000006	0.000002	0.0044	0.032
**Lungs**	0.0000053	0.000005	0.0051	0.022
**Liver**	0.000050	0.000008	0.020	0.076
**Spleen**	0.000009	0.000006	0.010	0.085
**Stomach**	0.000003	0.000002	0.003	0.011
**Sm. Intestine**	0.000009	0.000009	0.003	0.020
**L. Intestine**	0.000009	0.000010	0.005	0.015
**Kidney**	0.000042	0.000044	0.023	0.050
**Muscle**	0.000008	0.000001	0.004	0.008
**Bone**	0.000003	0.000008	0.020	0.050

Table S5: Administered tumor and kidney doses. For all emissions, RBE =1.

Group	Injected activity	Tumor dose (Gy)	Kidney dose (Gy)
^{177}Lu monotherapy	66.6 MBq	37.3	2.80
^{225}Ac monotherapy	74 kBq	37.7	1.7
^{177}Lu + ^{225}Ac	62.9 MBq + 18.5 kBq	38.5	3.7
^{177}Lu + ^{225}Ac	62.9 MBq + 37 kBq	58.1	4.62
^{177}Lu + ^{225}Ac	62.9 MBq + 74 kBq	97.31	6.47

SW1222 therapy: Study 1

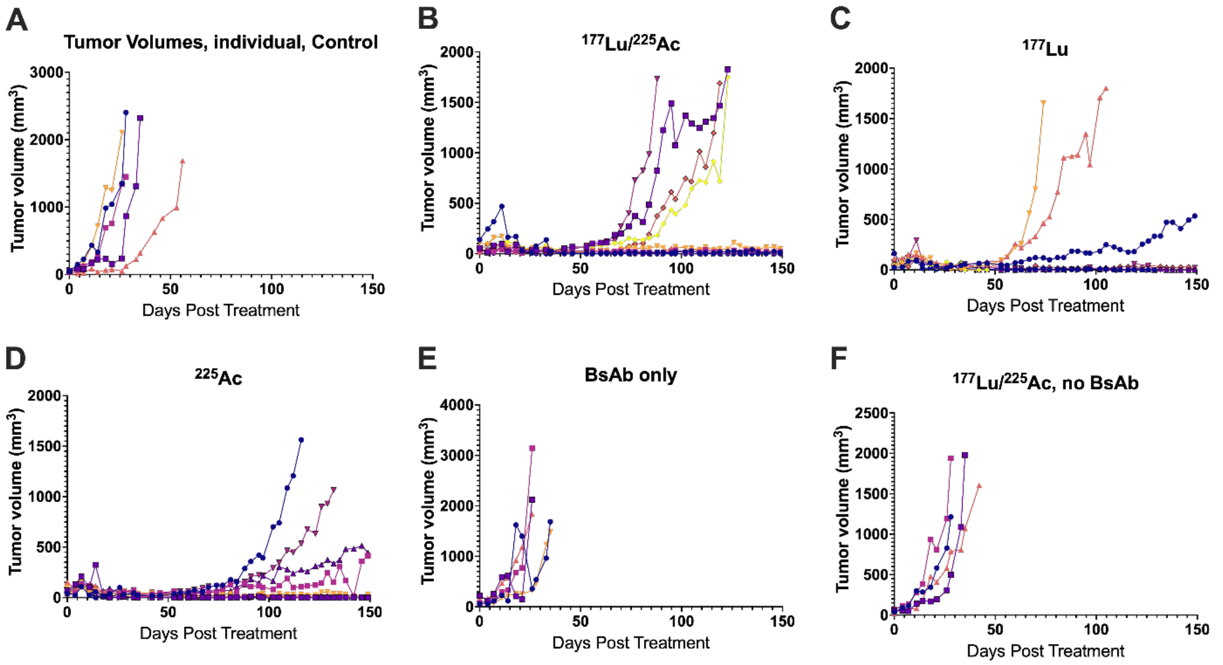

Figure S5: Individual tumor volumes in Study 1. Mice with established SW1222 xenografts (n=5-10/group) were treated with anti-GPA33 BsAb 24 h prior to administration a ^{177}Lu/^{225}Ac cocktail (62.9 MBq/18.5 kBq), 66.6 MBq ^{177}Lu or 74 kBq of ^{225}Ac.

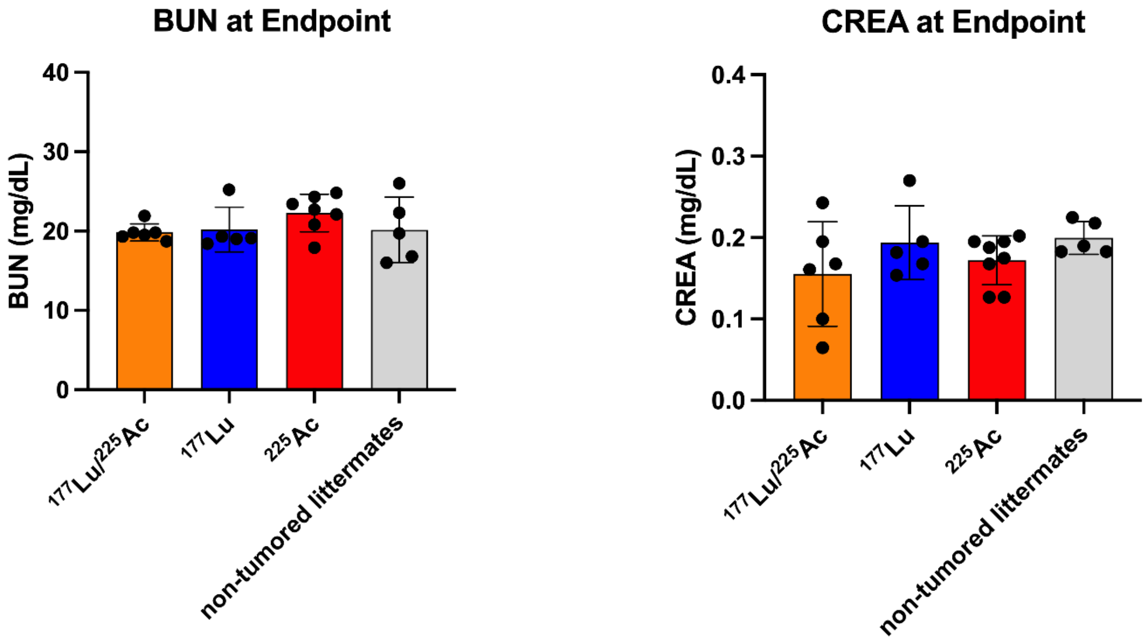

Figure S6: BUN and CREA of groups treated with ^{177}Lu- or ^{225}Ac monotherapy or ^{177}Lu/^{225}Ac (Study 1) at study endpoint 150 d compared with BUN and CREA of non-tumored littermates. Average ± 1SD.

SW1222 therapy: Study 2

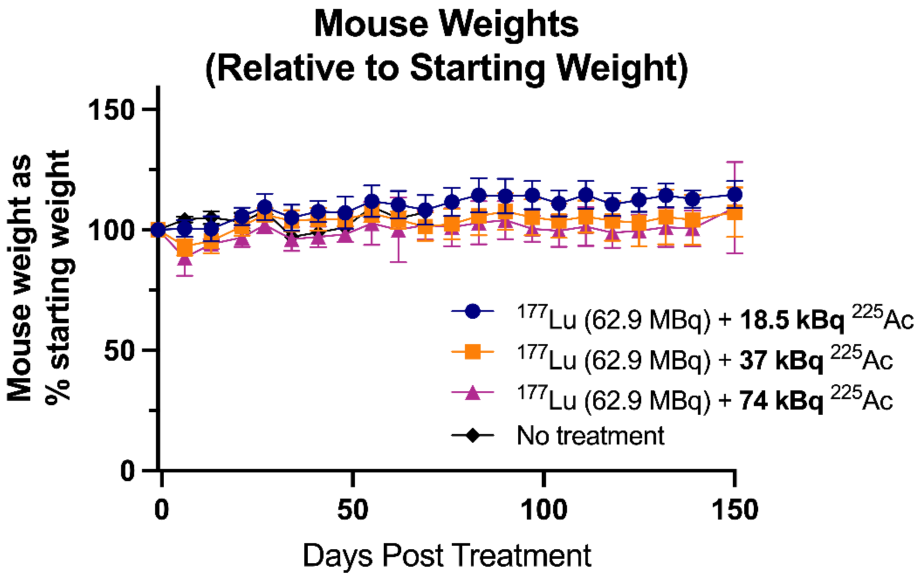

Figure S7: Mouse weight relative to starting weight. Mice with established SW1222 xenografts were treated with one cycle of anti-GPA33 BsAb 24 hours prior to administration a $^{177}Lu/^{225}Ac$ cocktail of a fixed dose of ^{177}Lu (62.9 MBq) with increasing doses of ^{225}Ac (18.5 kBq to 74 kBq). *n* = 4-10 animals/data point. Average ± 1SD.

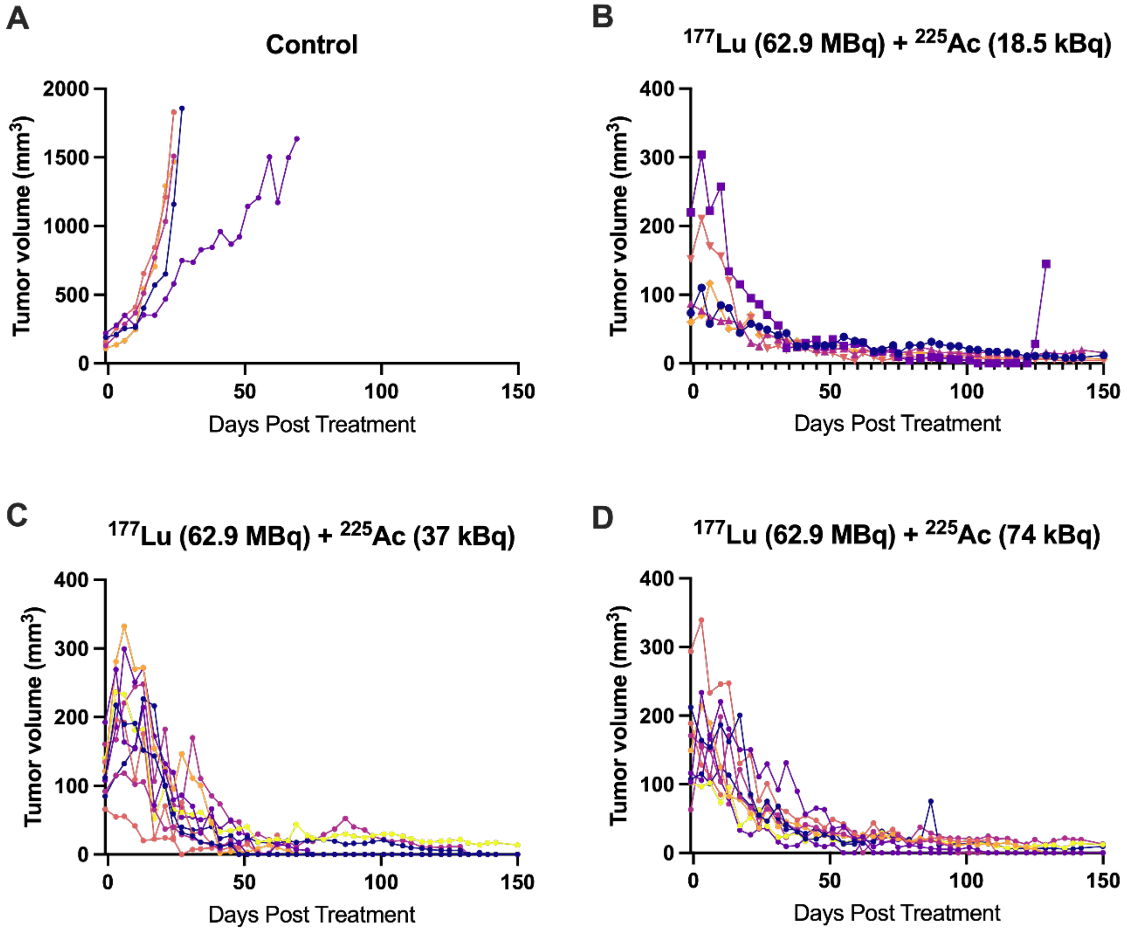

Figure S8: Individual tumor volumes in Study 2. Mice with established SW1222 xenografts (n=5-10/group) were injected with anti-GPA33 BsAb 24 hours prior to administration a ^{177}Lu/^{225}Ac cocktail of a fixed dose of ^{177}Lu (62.9 MBq) with increasing doses of ^{225}Ac (18.5 kBq to 74 kBq). One mouse in the ^{177}Lu+18.5 kBq ^{225}Ac was euthanized because of tumor burden around in the neck area.

Table S6: Survival and Tumor status of survivors 150 d after DOTA-PRIT therapy in mice bearing SW1222 xenografts.

Group	Median Survival (days)	Alive at 150 d (residual mass)	Tumor Status at 150d
		**Study 1**	
No treatment	32	0/5	n/a
BsAb only	27	0/5	n/a
^{177}Lu/^{225}Ac only	31	0/4	n/a
BsAb + **^{177}Lu** (66.6 MBq)	n/a	4/8; (534.2 mm^3, 0 mm^3, 20.2 mm^3, 0 mm^3)	M1: Viable adenocarcinoma with marked central necrosis.<br>M6-8: histological cures
BsAb + **^{225}Ac** (74 kBq)	n/a	8/10:<br>(415.3 mm^3, 0 mm^3, 28.4 mm^3, 0 mm^3, 0 mm^3, 445.1 mm^3, 0 mm^3, 0 mm$^{3)}$	M2: Viable adenocarcinoma with marked central necrosis and mineralization.<br>M3: Minimal numbers of viable neoplastic cells scattered within an eosinophilic matrix, and surrounded by fibrosis, and mononuclear infiltrates.<br>M4-6: histological cures

			M7: Viable adenocarcinoma with marked central necrosis.<br>M9, 10: histological cures; small remnants of an eosinophilic matrix.
BsAb + **^{177}Lu/^{225}Ac**<br>(62.9 MBq/18.5 kBq)	n/a	5/9<br>(12.7 mm^3, 18.2 mm^3, 26.0 mm^3, 46.9 mm^3, 0 mm^3,)	M1: histological cure; osinophilic matrix with mineralization and pigment-laden macrophage infiltrates.<br>M2, 3: histological cures<br>M4: Small cluster of viable neoplastic cells (~0.5 mm in diameter) surrounded by mucin, fibrosis, and mononuclear infiltrates.<br>M6: histological cures
**Study 2**			
No treatment	27	0/5	n/a
BsAb + ^{177}Lu/^{225}Ac<br>(62.9 MBq/**18.5 kBq)**	n/a	4/5<br>(11.8 mm^3, 15.4 mm^3, 4.5 mm^3, 6.3 mm^3)	1: histological cure<br>3: Minimal numbers of viable neoplastic cells<br>4,5: histological cure
BsAb + ^{177}Lu/^{225}Ac<br>(62.9 MBq/**37 kBq**)	n/a	10/10<br>(2: 14.0 mm^3, all others 0 mm^3)	1-10: histological cure
BsAb + ^{177}Lu/^{225}Ac<br>(62.9 MBq/**74 kBq**)	n/a	8/10<br>(12.1 mm^3, 9.6 mm^3, 0 mm^3, 12.8 mm^3, all remaining 0 mm^3)	1,2: Minimal numbers of viable neoplastic cells<br>3-4,7-10: histological cure

Table S7: Full pathology report of mice included in Study 1. Mice were euthanized at study endpoint unless otherwise specified. Mice that are not listed were euthanized due tumor burden and not submitted for histopathological evaluation. Evaluation included the following tissues: Tumor/injection site, Kidneys, Liver, Gallbladder, Spleen, Skin, Mammary glands, Bones, Bone marrow, Stifle Joint, Skeletal muscles, Nerves unless mice were submitted before study endpoint. In those cases, mice underwent complete necropsy to determine the cause of clinical decline.

**Animal ID**	**Anatomic pathology – Study 1**	
	**Gross Finding(s)**	**Microscopic Finding(s)**<br>**(All tissues are normal unless otherwise described.)**
M1/<br>BsAb +<br>$^{177}Lu/^{225}Ac$<br>(62.9 MBq/18.5 kBq)	• Bodyweight: 22.637 g<br>• No tumor remnants identified.<br>• The spleen is mildly enlarged. The liver has an enhanced reticular pattern.	• Tumor site: No tumor cells; eosinophilic matrix with mineralization and pigment-laden macrophage infiltrates, 2, F.<br>• Lungs: Mononuclear perivascular, peribronchiolar, or subpleural infiltrates, 1, MF.<br>• Kidneys: Tubular degeneration, 1, MF, cortical. Mononuclear interstitial infiltrates, 1, MF, cortical and pelvic.<br>• Liver: Mononuclear and neutrophilic perivascular/peribiliary aggregates, 1, MF. Extramedullary myelopoiesis, 1, MF, random. Increased glycogen accumulation, 3, D.<br>• Stomach: Mononuclear and neutrophilic gastritis, 3, MF.<br>• Salivary glands: Mononuclear perivascular to interstitial aggregates, 2, MF.<br>• Submandibular lymph nodes: Medullary plasmacytosis, 2, MF.<br>• Vagina: Mononuclear and neutrophilic, 4, MF, submucosal/muscularis.<br>• Ovaries: Pigment-laden macrophage infiltrates, 2, MF.<br>• Spleen: Lymphoid follicular hyperplasia, 3, MF.<br>• Adrenal Gland: Accessory adrenocortical nodule, F, UL. Subcapsular spindle cell hyperplasia, 2, MF, UL.<br>• Stifle Joint: Mononuclear and neutrophilic infiltrates, 2, F.<br>• Skeletal Muscle: Mononuclear interstitial infiltrates, 1, MF.<br>• Eyes: Mononuclear and neutrophilic endophthalmitis with lens degeneration and retinal atrophy, 3, D, UL.<br>• Harderian gland: Acinar degeneration and porphyrin deposits, 1, MF, BL. Mononuclear interstitial aggregates, 2, MF, BL.<br>• Brain: Mononuclear meningeal perivascular aggregates, 1, MF.<br>• Ears: Histiocytic otitis media, 2, F.<br>• Other: Mesentery and neck/limb soft tissues: neutrophilic to pyogranulomatous peritonitis/steatitis, 2, MF.
M2/<br>BsAb +<br>$^{177}Lu/^{225}Ac$<br>(62.9 MBq/18.5 kBq)	• Bodyweight: 26.463 g<br>• No tumor remnants identified.<br>• Unremarkable	• Lungs: Mononuclear perivascular, peribronchiolar, or subpleural infiltrates, 1, MF.<br>• Kidneys: Tubular degeneration, 1, MF, cortical. Mononuclear and neutrophilic interstitial infiltrates, 1, MF, pelvic.<br>• Liver: Mononuclear perivascular/peribiliary infiltrates, 1, MF. Extramedullary myelopoiesis, 1, MF, random.<br>• Mesenteric Lymphnode: Medullary plasmacytosis, 2, MF.

		• Salivary Glands: Mononuclear perivascular to interstitial aggregates, 1, MF.<br>• Submandibular lymph node: Medullary plasmacytosis, 3, MF.<br>• Urinary Bladder: Mononuclear perivascular aggregates, 1, MF.<br>• Ovaries: Pigment-laden macrophage infiltrates, 3, MF.<br>• Pancreas: Mononuclear perivascular to interstitial aggregates, 1, MF.<br>• Adrenals: Accessory adrenocortical nodule, F, UL. Subcapsular spindle cell hyperplasia, 2, MF, BL.<br>• Harderian gland: Acinar degeneration and porphyrin deposits, 2, MF, BL. Mononuclear interstitial aggregates, 2, MF, UL.
M3/<br>BsAb +<br>^{177}Lu/^{225}Ac<br>(62.9 MBq/18.5 kBq)	• Bodyweight: 27.065 g<br>• No tumor remnants identified.<br>• Unremarkable.	• Lungs: Mononuclear perivascular, peribronchiolar, or subpleural infiltrates, 1, MF.<br>• Kidneys: Tubular degeneration, 1, MF, cortical. Mononuclear perivascular to interstitial infiltrates, 1, MF, cortical and pelvic.<br>• Liver: Mononuclear perivascular/peribiliary infiltrates, 1, MF. Neutrophilic infiltrates, 1, MF, random.<br>• Submandibular Lymph node: Medullary plasmacytosis, 3, MF.<br>• Urinary bladder: Mononuclear perivascular aggregate, 1, MF.<br>• Uterus: Atrophy, D.<br>• Ovaries: Atrophy, D, BL.<br>• Oviducts: Epithelial vacuolation, 3, MF.<br>• Adrenals: Accessory adrenocortical nodule, F, UL. Subcapsular spindle cell hyperplasia, 2, MF, BL.<br>• Harderian Gland: Porphyrin deposits, 1, MF, BL.<br>• Ears: Neutrophilic otitis media with hyperkeratosis, 3, F, UL.
M4/<br>BsAb +<br>^{177}Lu/^{225}Ac<br>(62.9 MBq/18.5 kBq)	• Bodyweight: 24.776 g<br>• The subcutaneous tissue on the right flank has a small, approximately 0.4 x 0.3 x 0.2 cm raised area (suspected tumor remnants).	• Tumor site: Small cluster of viable neoplastic cells (~0.5 mm in diameter) surrounded by mucin, fibrosis, and mononuclear infiltrates.<br>• Skin (flank): Acanthosis and hyperkeratosis, 2, MF. Mononuclear and neutrophilic dermatitis and mural folliculitis, 1, MF.<br>• Lungs: Mononuclear perivascular, peribronchiolar, or subpleural infiltrates, 1, MF.<br>• Kidneys: Mononuclear tubulointerstitial nephritis with giant cells, 4, MF, cortical.<br>• Liver: Mononuclear periportal aggregates with pigment-laden macrophages, fibrosis, parenchymal loss, and mineralization, 2, MF.<br>• Stomach: Neutrophilic gastritis, 3, MF.<br>• Submandibular Lymph node: Medullary plasmacytosis, 2, MF.<br>• Oviducts: Epithelial vacuolation, 2, MF.<br>• Adrenals: Accessory adrenocortical nodules, MF, UL.<br>• Trachea: Mononuclear interstitial infiltrates, 1, MF, lamina propria.<br>• Eyes: Iris mineralization, 2, MF, BL.

		• Harderian Gland: Acinar degeneration and porphyrin deposits, 1, MF, BL. Mononuclear interstitial aggregates, 2, MF, BL.<br>• Other: Mesentery: mononuclear perivascular aggregates, 2, MF (slide 5).
M5/<br>BsAb +<br>^{177}Lu/^{225}Ac<br>(62.9 MBq/18.5 kBq)	• Bodyweight: 22.894 g<br>• The right flank has a 2.0 x 1.6 x 1.2 cm subcutaneous mass.	• Tumor site: Viable adenocarcinoma with marked multifocal necrosis.<br>• Skin (flank): Acanthosis and hyperkeratosis, 2, MF. Mononuclear and neutrophilic mural folliculitis, 2, MF.<br>• Heart: Mononuclear infiltrates, 2, MF.<br>• Lungs: Mononuclear perivascular, peribronchiolar, or subpleural infiltrates, 1, MF.<br>• Thymus: Thymic cysts, MF.<br>• Liver: Mononuclear perivascular/peribiliary infiltrates, 1, MF.<br>• Mesenteric Lymph node: Lymphatic ectasia, 3, F.<br>• Oviducts: Epithelial vacuolation, 2, MF.<br>• Spleen: Plasmacytosis, 2, MF.<br>• Adrenals: Subcapsular spindle cell hyperplasia, 2, MF, BL.<br>• Trachea: Mononuclear interstitial infiltrates, 1, MF, lamina propria.<br>• Skeletal Muscle: Mononuclear interstitial infiltrates, 1, MF.<br>• Oral cavity: Mononuclear and neutrophilic glossitis/myositis with fibrosis, 3, MF.<br>• Nasal cavity: Neutrophilic luminal infiltrates with entrapped foreign material, 2, F, nasopharyngeal duct.<br>• Harderian Gland: Acinar degeneration with porphyrin deposits, 1, MF, BL.<br>• Ears: Neutrophilic and histiocytic otitis media, 4, D, BL. Hyperkeratosis with intracorneal mixed bacteria, 3, F, UL
M6/<br>BsAb +<br>^{177}Lu/^{225}Ac<br>(62.9 MBq/18.5 kBq)	• Bodyweight: 21.866 g<br>• No tumor remnants identified.<br>• Unremarkable.	• Tumor/injection site: No tumor remnants.<br>• Skin (flank): Acanthosis and hyperkeratosis, 2, MF. Mononuclear dermatitis, mural folliculitis, and panniculitis, 2, MF.<br>• Lungs: Mononuclear perivascular, peribronchiolar, or subpleural infiltrates, 1, MF.<br>• Thymus: Thymic cysts, MF.<br>• Liver: Mononuclear perivascular/peribiliary infiltrates, 1, MF.<br>• Urinary bladder: Mononuclear perivascular aggregates, 1, MF.<br>• Ovaries: Atrophy, D, UL (only one ovary available).<br>• Oviducts: Epithelial vacuolation, 2, MF.<br>• Adrenals: Accessory adrenocortical nodule, F, UL. Subcapsular spindle cell hyperplasia, 2, MF, UL.<br>• Trachea: Mononuclear interstitial infiltrates, 1, MF, lamina propria.<br>• Thyroid: Mononuclear thyroiditis with follicular degeneration, 3, MF.<br>• Eyes: Mononuclear and neutrophilic keratitis and blepharitis, 2, MF, UL.<br>• Harderian gland: Acinar degeneration and porphyrin deposits, 3, MF, UL. Mononuclear interstitial aggregates, 2, MF, BL. Peri-Harderian soft tissue: epidermal cyst, F.

		• Ears: Mononuclear otitis externa with hyperkeratosis and intracorneal bacterial colonies, 2, MF, BL.
M7/ BsAb + $^{177}Lu/^{225}Ac$ (62.9 MBq/18.5 kBq)	• Bodyweight: 24.921 g<br>• The right flank has a 2.0 x 1.5 x 0.5 cm subcutaneous mass.	• Tumor/Injection site: Viable adenocarcinoma with marked multifocal necrosis.<br>• Kidneys, Tubular degeneration, 2, MF, cortical. Mononuclear infiltrates, 1, MF, cortical.<br>• Skin (flank): Acanthosis and hyperkeratosis, 2, MF. Mononuclear infiltrates, 1, MF.
M8/ BsAb + $^{177}Lu/^{225}Ac$ (62.9 MBq/18.5 kBq)	• Bodyweight: 24.413 g<br>• The right flank has a 2.4 x 1.6 x 1.4 cm subcutaneous mass that is partially adhered to the hind limb musculature.	• Tumor/Injection site: Viable adenocarcinoma with marked multifocal necrosis.<br>• Skin (flank): Acanthosis and hyperkeratosis, 2, MF. Mononuclear infiltrates and dermal fibrosis, 1, MF. Pyogranulomatous panniculitis, 1, F.<br>• Lungs: Mononuclear perivascular, peribronchiolar, or subpleural aggregates, 1, MF.<br>• Thymus: Thymic cysts, MF.<br>• Kidneys: Tubular degeneration, 2, MF, cortical. Mononuclear infiltrates, 1, MF, cortical.<br>• Liver: Mononuclear perivascular/peribiliary infiltrates, 1, MF. Neutrophilic infiltrates, 1, MF, random.<br>• Spleen: Capsular fibrosis, 2, F.<br>• Adrenals: Subcapsular spindle cell hyperplasia, 2, MF, UL.<br>• Nasal Cavity: Neutrophilic rhinitis, 3, MF, UL.<br>• Hardarian Gland: Acinar degeneration with porphyrin deposits, 1, MF, BL.<br>• Ears: Neutrophilic and histiocytic otitis media, 2-3, MF, BL. Hyperkeratosis with intracorneal mixed bacteria, 2, F, UL.<br>• Mesentery and perigenital/limb soft tissues: pyogranulomatous peritonitis/steatitis, 2, MF.
M9/ BsAb + $^{177}Lu/^{225}Ac$ (62.9 MBq/18.5 kBq)	• Bodyweight: 25.753 g<br>• The right flank has a 2.5 x 1.5 x 1.0 cm subcutaneous mass.	• Tumor/injection site: Viable adenocarcinoma with marked central necrosis and multifocal mineralization.<br>• Lungs: Mononuclear perivascular, peribronchiolar, or subpleural aggregates, 1, MF.<br>• Thymus: Thymic cysts, MF.<br>• Liver: Mononuclear perivascular/peribiliary aggregates, 1, MF. Neutrophilic infiltrates, 1, MF, random.<br>• Gall Bladder: Mononuclear and neutrophilic infiltrates, 2, D.<br>• Salivary Gland: Mononuclear perivascular to interstitial aggregates, 1, MF.<br>• Submandibular Lymph node : Medullary plasmacytosis, 2, MF.<br>• Urinary Bladder: Mononuclear perivascular aggregate, 1, F.<br>• Uterus: Atrophy, D.<br>• Vagina: Mononuclear infiltrates, 2, MF, perigenital musculature/interstitium.<br>• Ovaries: Atrophy, UL.<br>• Oviducts: Epithelial vacuolation, 2, MF.<br>• Pancreas: Mononuclear perivascular aggregate, 1, F.<br>• Adrenals: Subcapsular spindle cell hyperplasia, 1, MF, UL.

		• Trachea: Mononuclear interstitial infiltrates, 1, MF, lamina propria.<br>• Nasal Cavity: Epithelial hyalinosis, 3, D, BL.<br>• Harderian gland: Porphyrin deposits, 1, MF, BL.<br>• Other: Meibomian gland: pyogranulomatous adenitis, 2, MF, BL.
M1/<br>BsAb + ^{177}Lu<br>(66.6 MBq)	• Bodyweight: 21.739 g<br>• The right flank has a 1.2 x 0.9 x 0.6 cm subcutaneous mass.	• Tumor/injection site: Viable adenocarcinoma with marked central necrosis.<br>• Kidneys: Tubular degeneration, 1, MF, cortical.<br>• Liver: Mononuclear perivascular/peribiliary infiltrates, 1, MF.<br>• Skeletal Muscle: Mononuclear and neutrophilic interstitial infiltrates, 1, MF.
M4/<br>BsAb + ^{177}Lu<br>(66.6 MBq)	• Bodyweight: 14.947 g<br>• The subcutaneous tissue on the right flank has an approximately 0.7 x 0.3 x 0.3 cm raised area (suspected tumor remnants).<br>• The skin throughout the body is multifocally thickened and flaky.	Mouse was euthanized on day 35 due to weight loss >20%<br><br>• Tumor/Injection site: Multifocal small clusters of viable neoplastic cells embedded in an eosinophilic matrix with multifocal osseous metaplasia, mononuclear infiltrates, and surrounded by fibrosis.<br>• Skin (flank): Acanthosis and hyperkeratosis, 3, MF. Mononuclear infiltrates, 1, MF.<br>• Lungs: Mononuclear perivascular, peribronchiolar, or subpleural infiltrates, 1, MF.<br>• Thymus: Thymic cysts, MF.<br>• Liver: Mononuclear perivascular/peribiliary infiltrates, 1, MF.<br>• Oviducts: Epithelial vacuolation, 2, MF.<br>• Adrenals: Accessory adrenocortical nodules, MF, BL.<br>• Bone Marrow: Hypocellularity, 2, MF.<br>• Skeletal Muscle: Mononuclear and neutrophilic interstitial infiltrates, 1, MF.<br>• Nerves: Mononuclear and neutrophilic infiltrates, 1, MF. Axonal degeneration, 2, MF.<br>• Spinal Cord: Mononuclear meningeal perivascular aggregate, 1, F.<br>• Oral Cavity: Mononuclear and neutrophilic infiltrates, 1, MF, tongue.<br>• Harderian Gland: Porphyrin deposits, 1, MF, BL.
M5/<br>BsAb + ^{177}Lu<br>(66.6 MBq)	• No tumor remnants identified	Mouse was found dead on day 130, limited histopathological analysis possible due to postmortem autolysis<br><br>• Tumor/Injection site: No tumor remnants.<br>• Lungs: Mononuclear perivascular, peribronchiolar, or subpleural aggregates, 1, MF.<br>• Kidneys: Presumptive glomerulonephropathy, 4, D (interpretation hindered by severe autolysis).<br>• Liver: Mononuclear perivascular/peribiliary infiltrates, 1, MF.<br>• Harderian Gland: Porphyrin deposits, 1, MF, UL.
M6/<br>BsAb + ^{177}Lu<br>(66.6 MBq)	• Bodyweight: 25.283 g	• Tumor/injection site: No tumor remnants.<br>• Skin (flank): Acanthosis and hyperkeratosis, 2, MF. Mononuclear and neutrophilic dermatitis, mural folliculitis, and panniculitis 1, MF.

	• No tumor remnants identified.<br>• Unremarkable.	• Kidneys: Tubular degeneration, 1, MF, cortical. Mononuclear interstitial infiltrates, 1, MF, pelvic.<br>• Liver: Mononuclear perivascular/peribiliary infiltrates, 1, MF.<br>• Skeletal Muscle: Mononuclear myositis, 2, MF.
M7/<br>BsAb + ^{177}Lu<br>(66.6 MBq)	• Bodyweight: 26.84 g<br>• No tumor remnants identified.<br>• Unremarkable.	• Tumor/injection site: No tumor remnants.<br>• Skin (flank): Acanthosis and hyperkeratosis, 2, MF. Mononuclear infiltrates with fibrosis, 1, MF.<br>• Liver: Mononuclear perivascular/peribiliary infiltrates, 1, MF.<br>• Gallbladder: Mononuclear interstitial aggregates, 1, MF.
M8/<br>BsAb + ^{177}Lu<br>(66.6 MBq)	• Bodyweight: 26.635 g<br>• No tumor remnants identified.<br>• Unremarkable.	• Tumor/injection site: No tumor remnants.<br>• Skin (flank): Acanthosis and hyperkeratosis, 2, MF. Mononuclear infiltrates, 1, MF.<br>• Kidneys: Tubular degeneration, 1, MF, cortical.<br>• Liver: Mononuclear perivascular/peribiliary infiltrates, 1, MF. Neutrophilic infiltrates, 1, MF, random.
M2/<br>BsAb + ^{225}Ac<br>(74 kBq)	• Bodyweight: 25.497 g<br>• The right flank has a 1.0 x 1.0 x 0.4 cm subcutaneous mass.	• Tumor/injection site: Viable adenocarcinoma with marked central necrosis and mineralization.<br>• Kidneys: Tubular degeneration, 1, MF, cortical. Mononuclear interstitial infiltrates, 1, MF, cortical and pelvic.<br>• Liver: Mononuclear perivascular/peribiliary infiltrates, 1, MF. Neutrophilic infiltrates, 1, MF, random.<br>• Skin (flank): Acanthosis and hyperkeratosis, 2, MF. Mononuclear dermatitis and mural folliculitis with fibrosis, 1, MF.
M3/<br>BsAb + ^{225}Ac<br>(74 kBq)	• Bodyweight: 26.286 g<br>• No tumor remnants identified. Unremarkable.	• Tumor/injection site: Minimal numbers of viable neoplastic cells scattered within an eosinophilic matrix, and surrounded by fibrosis, and mononuclear infiltrates.<br>• Kidneys: Tubular degeneration, 1, MF, cortical. Mononuclear interstitial infiltrates, 1, MF, pelvic.<br>• Liver: Mononuclear perivascular/peribiliary infiltrates, 1, MF.<br>• Skin (flank): Acanthosis and hyperkeratosis, 2, MF. Mononuclear and neutrophilic dermatitis, 2, MF.
M4/<br>BsAb + ^{225}Ac<br>(74 kBq)	• Bodyweight: 25.52 g<br>• No tumor remnants identified.<br>• Unremarkable.	• Tumor/injection site: No tumor remnants.<br>• Skin (flank): Acanthosis and hyperkeratosis, 2, MF.<br>Mononuclear and neutrophilic dermatitis, mural folliculitis, and panniculitis 2, MF.<br>• Kidneys: Tubular degeneration, 1, MF, cortical. Mononuclear and neutrophilic interstitial infiltrates, 2, MF, pelvic and cortical. Hyaline glomerulopathy, 2, MF, segmental.<br>• Liver: Mononuclear perivascular/peribiliary infiltrates, 1, MF. Neutrophilic infiltrates, 1, MF, random.<br>• Skeletal Muscle: Mononuclear and neutrophilic interstitial infiltrates, 1, F.
M5/<br>BsAb + ^{225}Ac	• Bodyweight: 22.18 g	• Tumor/injection site: No tumor remnants.<br>• Skin (flank): Acanthosis and hyperkeratosis, 2, MF. Mononuclear infiltrates, 1, MF.

(74 kBq)	• No tumor remnants identified.<br>• Unremarkable.	• Liver: Mononuclear perivascular/peribiliary aggregates, 2, MF. Extramedullary hematopoiesis, 1, MF, random. Individual hepatocellular necrosis, 1, MF, random.<br>• Skeletal muscle: Mononuclear and neutrophilic interstitial infiltrates, 1, MF.
M6/<br>BsAb + ^{225}Ac<br>(74 kBq)	• Bodyweight: 21.04 g<br>• No tumor remnants identified.<br>• Unremarkable.	• Tumor/injection site: No tumor remnants.<br>• Skin (flank): Acanthosis and hyperkeratosis, 2, MF. Mononuclear dermatitis and mural folliculitis, 2, MF.<br>• Kidney: Mononuclear perivascular to interstitial infiltrates, 1, MF, cortical and pelvic.<br>• Liver: Mononuclear perivascular/peribiliary infiltrates, 1, MF.<br>• Gall Bladder: Mononuclear interstitial aggregate, 1, F.
M7/<br>BsAb + ^{225}Ac<br>(74 kBq)	• Bodyweight: 21.851 g<br>• No tumor remnants identified.<br>• Unremarkable.	• Tumor/Injection site: Viable adenocarcinoma with marked central necrosis.<br>• Skin (flank): Acanthosis and hyperkeratosis, 2, MF. Mononuclear and neutrophilic dermatitis, 1, MF.<br>• Liver: Mononuclear and neutrophilic perivascular/peribiliary aggregates, 2, MF. Histiocytic aggregates, 2, MF, random. Extramedullary hematopoiesis, 2, MF, random.<br>• Spleen: Histiocytic aggregates, 3, MF. Increased extramedullary hematopoiesis, 3, MF.<br>• Bone Marrow: Myeloid hyperplasia, 2, MF.
M9/<br>BsAb + ^{225}Ac<br>(74 kBq)	• Bodyweight: 20.967 g<br>• No tumor remnants identified.<br>• Unremarkable.	• Tumor/injection site: No tumor cells; small remnants of an eosinophilic matrix.<br>• Skin (flank): Acanthosis and hyperkeratosis, 2, MF. Mononuclear dermatitis, 2, MF.<br>• Kidneys: Mononuclear perivascular to interstitial infiltrates, 1, MF, cortical and pelvic.<br>• Liver: Mononuclear perivascular/peribiliary infiltrates, 1, MF.<br>• Other: Hind limb adipose tissue: neutrophilic steatitis, 3, F.
M10/<br>BsAb + ^{225}Ac<br>(74 kBq)	• Bodyweight: 21.368 g<br>• No tumor remnants identified.<br>• Unremarkable.	• Tumor/injection site: No tumor cells; small remnants of an eosinophilic matrix.<br>• Kidneys: Tubular degeneration, 1, MF, cortical.<br>• Liver: Mononuclear perivascular/peribiliary infiltrates, 1, MF. Extramedullary hematopoiesis, 1, MF, random.<br>• Skin (flank): Acanthosis and hyperkeratosis, 2, MF. Mononuclear infiltrates, 1, MF.
M3/<br>Untreated control	• Bodyweight: 18.316 g<br>• The right flank has a 2.5 x 1.2 x 1.1 cm subcutaneous mass.	Euthanized on day 34<br>• Tumor/injection site: Viable adenocarcinoma with moderate multifocal necrosis.<br>• Skin (flank): Acanthosis and hyperkeratosis, 2, MF. Mononuclear and neutrophilic dermatitis, 1, MF.
M2/	• Bodyweight: 19.846 g	Euthanized on day 28<br>• Tumor/injection site: Viable adenocarcinoma with moderate multifocal necrosis.

BsAB only control	• The right flank has a 3.0 x 1.5 x 1.4 cm subcutaneous mass.<br>• The right kidney has a 0.1 cm in diameter cortical cyst.	• Skin (flank): Acanthosis and hyperkeratosis, 2, MF. Mononuclear and neutrophilic dermatitis and folliculitis with dermal fibrosis, 2, MF.<br>• Kidneys: Cyst, F, cortical. Tubular degeneration, 1, MF, cortical, pericystic.
M5/ ^{177}Lu/^{225}Ac only (62.9 MBq/18.5 kBq)	• Bodyweight: 22.191 g<br>• The right flank has a 2.5 x 2.2 x 1.3 subcutaneous mass.	• Tumor/injection site: Viable adenocarcinoma with dilated vascular channels and mild multifocal necrosis.<br>• Kidneys: Tubular degeneration, 1, MF, cortical.
N: Normal F:Focal. MF: Multifocal: D: Diffuse. UL: Unilateral: BL: Bilateral. 1: Minimal. 2: Mild. 3: Moderate. 4: Marked		

Table S8: Full pathology report of mice included in Study 1. Mice were euthanized at study endpoint unless otherwise specified. Mice that are not listed were euthanized due tumor burden and not submitted for histopathological evaluation. Evaluation included the following tissues: Tumor/injection site, Kidneys, Liver, Gallbladder, Spleen, Skin, Mammary glands, Bones, Bone marrow, Stifle Joint, Skeletal muscles, Nerves unless mice were submitted before study endpoint. In those cases, mice underwent complete necropsy to determine the cause of clinical decline.

**Animal ID**	**Anatomic pathology – Study 2**	
	**Gross Finding(s)**	**Microscopic Finding(s) (All tissues are normal unless otherwise described.)**
M1/ BsAb + ^{177}Lu/^{225}Ac (62.9 MBq/18.5 kBq)	• Bodyweight: 26.548 g	• Tumor/Injection site: No tumor remnants.<br>• Skin (flank): Acanthosis and hyperkeratosis, 1, MF.<br>• Kidneys: Tubular degeneration, 2, MF, cortical > medullary. Mononuclear infiltrates with pigment-laden macrophages, 1, MF, cortical. Kidneys: Segmental hyaline glomerulopathy, 1, MF.<br>• Liver: Mononuclear perivascular/peribiliary infiltrates, 1, MF.<br>• Spleen: Plasmacytosis, 1, MF.
M3/ BsAb + ^{177}Lu/^{225}Ac (62.9 MBq/18.5 kBq)	• Bodyweight: 22.965 g<br>• The subcutaneous tissue on the right flank has an approximately 0.1 cm in diameter raised area	• Tumor/Injection site: Minimal numbers of viable neoplastic cells at the periphery of a nodule of osseous metaplasia surrounded by fibrosis and scattered mononuclear infiltrates.<br>• Skin (flank): Acanthosis and hyperkeratosis, 2, MF. Mononuclear and neutrophilic dermatitis and mural folliculitis, 1, MF.<br>• Kidneys: Tubular degeneration, 1, MF, cortical. Mononuclear infiltrates, 1, MF, pelvic.<br>• Liver: Mononuclear perivascular/peribiliary infiltrates, 1, MF.

	(suspected tumor remnants).	
M4/<br>BsAb +<br>^{177}Lu/^{225}Ac<br>(62.9 MBq/18.5 kBq)	• Bodyweight: 21.937 g	• Tumor/Injection site: No tumor remnants.<br>• Kidneys: Tubular degeneration, 1, MF, cortical.<br>• Liver: Mononuclear perivascular/peribiliary infiltrates, 1, MF.<br>• Skin (flank): Acanthosis and hyperkeratosis, 3, MF.
M5/<br>BsAb +<br>^{177}Lu/^{225}Ac<br>(62.9 MBq/18.5 kBq)	• Bodyweight: 24.829 g	• Tumor/Injection site: No tumor remnants.<br>• Skin (flank): Acanthosis and hyperkeratosis, 1, MF.<br>• Kidneys: Tubular degeneration, 1, MF, cortical. Tubular mineralization, 1, MF, medullary.<br>• Liver: Mononuclear perivascular/peribiliary infiltrates, 1, MF.
M1/<br>BsAb +<br>^{177}Lu/^{225}Ac<br>(62.9 MBq/37 kBq)	• Bodyweight: 23.943 g	• Tumor/injection site: No tumor remnants.<br>• Kidneys: Tubular degeneration, 1, MF, cortical.<br>• Liver: Mononuclear perivascular/peribiliary infiltrates, 1, MF.<br>• Skin (flank): Acanthosis and hyperkeratosis, 1, MF. Dermal fibrosis, 1, MF.
M2/<br>BsAb +<br>^{177}Lu/^{225}Ac<br>(62.9 MBq/37 kBq)	• Bodyweight: 20.25 g	• Tumor/injection site: No tumor remnants.<br>• Skin (flank): Acanthosis and hyperkeratosis, 2, MF. Mononuclear dermatitis and dermal fibrosis, 1, MF.<br>• Kidneys: Tubular degeneration, 1, MF, cortical. Tubular mineralization, 1, MF, medullary.<br>• Liver: Mononuclear perivascular/peribiliary infiltrates, 1, MF.
M3/<br>BsAb +<br>^{177}Lu/^{225}Ac<br>(62.9 MBq/37 kBq)	• Bodyweight: 24.817 g	• Tumor/injection site: No tumor remnants.<br>• Skin (flank): Acanthosis and hyperkeratosis, 2, MF. Mononuclear and neutrophilic dermatitis, 2, MF.<br>• Kidneys: Tubular degeneration, 2, MF, cortical.<br>• Liver: Mononuclear perivascular/peribiliary infiltrates, 1, MF.
M4/<br>BsAb +<br>^{177}Lu/^{225}Ac<br>(62.9 MBq/37 kBq)	• Bodyweight: 21.181 g	• Tumor/injection site: No tumor remnants.<br>• Skin (flank): Acanthosis and hyperkeratosis, 2, MF. Mononuclear dermatitis, 1, MF.<br>• Kidneys: Tubular degeneration, 2, MF, cortical. Mononuclear infiltrates, 1, MF, cortical and pelvic.<br>• Liver: Mononuclear perivascular/peribiliary infiltrates, 1, MF.
M5/<br>BsAb +<br>^{177}Lu/^{225}Ac<br>(62.9 MBq/37 kBq	• Bodyweight: 22.829 g	• Tumor/injection site: No tumor remnants.<br>• Skin (flank): Acanthosis and hyperkeratosis, 2, MF. Mononuclear dermatitis, 1, MF<br>• Kidneys: Tubular degeneration, 2, MF, cortical. Mononuclear infiltrates, 1, MF, cortical and pelvic. Tubular mineralization, 1, MF, medullary.

		• Liver: Mononuclear perivascular/peribiliary aggregates, 1, MF. Neutrophilic infiltrates, 1, MF, random.<br>• Spleen: Plasmacytosis, 2, MF.
M6/<br>BsAb +<br>^{177}Lu/^{225}Ac<br>(62.9 MBq/37 kBq	• Bodyweight: 18.275 g	• Tumor/injection site: No tumor remnants.<br>• Skin (flank): Acanthosis and hyperkeratosis, 2, MF. Mononuclear dermatitis and mural folliculitis, 1, MF.<br>• Kidneys: Tubular degeneration, 1, MF, cortical.<br>• Liver: Mononuclear perivascular/peribiliary aggregates, 1, MF.
M7/<br>BsAb +<br>^{177}Lu/^{225}Ac<br>(62.9 MBq/37 kBq	• Bodyweight: 19.953 g	• Tumor/injection site: No tumor remnants.<br>• Skin (flank): Acanthosis and hyperkeratosis, 3, MF. Dermal fibrosis, 1, MF.<br>• Kidneys: Tubular degeneration, 1, MF, cortical and medullary. Tubular mineralization, 1, MF, medullary.<br>• Liver: Mononuclear perivascular/peribiliary aggregates, 1, MF. Neutrophilic infiltrates, 1, MF, random.
M8/<br>BsAb +<br>^{177}Lu/^{225}Ac<br>(62.9 MBq/37 kBq	• Bodyweight: 22.396 g	• Tumor/injection site: No tumor remnants.<br>• Skin (flank): Acanthosis and hyperkeratosis, 1, MF.<br>• Kidneys: Tubular degeneration, 2, MF, cortical. Mononuclear and neutrophilic infiltrates, 2, MF, cortical and pelvic.<br>• Liver: Mononuclear perivascular/peribiliary infiltrates, 2, MF. Neutrophilic hepatitis, 1, MF, random.<br>• Spleen: Plasmacytosis, 2, MF. Neutrophilic infiltrates, 2, MF.<br>• Stifle joint: Mononuclear and neutrophilic arthritis, 3, D.<br>• Skeletal muscles (hind limb, spine, head): Mononuclear and neutrophilic interstitial infiltrates, 2, MF. Myofiber degeneration, 1, MF.
M9/<br>BsAb +<br>^{177}Lu/^{225}Ac<br>(62.9 MBq/37 kBq	• Bodyweight: 26.523 g	• Tumor/injection site: No tumor remnants.<br>• Skin (flank): Acanthosis and hyperkeratosis, 2, MF. Mononuclear dermatitis and dermal fibrosis, 1, MF.<br>• Kidneys: Tubular degeneration, 1, MF, cortical.<br>• Liver: Mononuclear perivascular/peribiliary infiltrates, 1, MF. Decreased glycogen deposits, 1, MF, periportal.
M10/<br>BsAb +<br>^{177}Lu/^{225}Ac<br>(62.9 MBq/37 kBq	• Bodyweight: 16.763 g	• Tumor/injection site: No tumor remnants.<br>• Kidneys: Tubular degeneration, 2, MF, cortical.<br>• Liver: Mononuclear perivascular/peribiliary infiltrates, 2, MF. Biliary hyperplasia with epithelial hyalinosis and peribiliary fibrosis, 2, MF. Decreased glycogen deposits, 1, MF, periportal.<br>• Skeletal muscles (hind limb, spine, head): Mononuclear and neutrophilic interstitial infiltrates, 2, MF.<br>• Nerves (hind limb, spine, head): Mononuclear and neutrophilic infiltrates, 2, MF.
M1/<br>BsAb +<br>^{177}Lu/^{225}Ac<br>(62.9 MBq/74 kBq	• Bodyweight: 23.108 g<br>• The subcutaneous tissue on the right flank has an	• Tumor/injection site: Minimal numbers of viable neoplastic cells scattered within an eosinophilic matrix and surrounded by fibrosis.<br>• Skin (flank): Acanthosis and hyperkeratosis, 2, MF. Mononuclear dermatitis, mural folliculitis, and dermal fibrosis, 1, MF.<br>• Kidneys: Tubular degeneration, 3, MF, cortical. Mononuclear and neutrophilic infiltrates, 2, MF, cortical.

	approximately 0.25 cm in diameter raised area (suspected tumor remnants). The kidneys are moderately pale.	• Liver: Mononuclear perivascular/peribiliary infiltrates, 1, MF. Neutrophilic hepatitis, 1, MF, random.
M2/ BsAb + $^{177}Lu/^{225}Ac$ (62.9 MBq/74 kBq	• Bodyweight: 17.909 g<br>• The subcutaneous tissue on the right flank has an approximately 0.1 cm in diameter raised area (suspected tumor remnants).	• Tumor/injection site: Minimal numbers of viable neoplastic cells scattered within an eosinophilic matrix and surrounded by fibrosis.<br>• Skin (flank): Acanthosis and hyperkeratosis, 2, MF. Mononuclear dermatitis and dermal fibrosis, 2, MF.<br>• Kidneys: Tubular degeneration, 3, MF, cortical. Tubular mineralization, 1, MF, medullary.<br>• Liver: Mononuclear perivascular/peribiliary aggregates, 1, MF. Neutrophilic infiltrates, 1, MF, random.<br>• Gallbladder: Mononuclear infiltrates, 1, MF.
M3/ BsAb + $^{177}Lu/^{225}Ac$ (62.9 MBq/74 kBq	• Bodyweight: 24.062 g<br>• The kidneys are moderately pale. The liver is mildly pale with enhanced reticular pattern.	• Tumor/injection site: No tumor remnants.<br>• Skin (flank): Acanthosis and hyperkeratosis, 2, MF.<br>• Kidneys: Tubular degeneration, 3, MF, cortical. Mononuclear and neutrophilic infiltrates, 2, MF, cortical. Tubular mineralization, 1, MF, medullary.<br>• Liver: Mononuclear hepatitis with occasional fibrosis and pigment-laden macrophages, 2, MF, random.
M4/ BsAb + $^{177}Lu/^{225}Ac$ (62.9 MBq/74 kBq	• Bodyweight: 18.462 g	• Tumor/injection site: No tumor remnants.<br>• Skin (flank): Acanthosis and hyperkeratosis, 2, MF. Mononuclear and neutrophilic dermatitis, 2, MF.<br>• Kidneys: Tubular degeneration, 3, MF, cortical.<br>• Liver: Mononuclear perivascular/peribiliary aggregates, 1, MF. Neutrophilic infiltrates, 1, MF, random.
M5/ BsAb + $^{177}Lu/^{225}Ac$ (62.9 MBq/74 kBq	• Bodyweight: 16.223 g<br>• The subcutaneous tissue on the right flank has a 0.8 x 0.6 x 0.3 cm mass.	Mouse was euthanized on day 14 due to weight loss >20%<br><br>• Tumor/injection site: Viable adenocarcinoma.<br>• Kidneys: Tubular degeneration, 2, F.<br>• Liver: Mononuclear perivascular/peribiliary infiltrates, 1, MF.<br>• Submandibular lymph node: Medullary plasmacytosis, 3, MF.<br>• Spleen: Red pulp (hematopoietic) hypocellularity, 4, D.

		• Skin (flank): Acanthosis and hyperkeratosis, 3, MF.<br>• Bone marrow (femur, tibia, sternum, vertebrae): Hypocellularity (all lineages), 4, MF.<br>• Harderian gland: Porphyrin deposits, 1, MF, BL.
M6/<br>BsAb +<br>$^{177}Lu/^{225}Ac$<br>(62.9 MBq/74 kBq	• Bodyweight: 14.188 g	Mouse was euthanized on day 63 due to weight loss >20%<br><br>• Tumor/injection site: No tumor remnants.<br>• Skin (flank): Acanthosis and hyperkeratosis, 2, MF. Mononuclear and neutrophilic dermatitis, 1, MF.<br>• Lungs: Mononuclear perivascular, peribronchiolar, or subpleural infiltrates, 1, MF.<br>• Liver: Neutrophilic hepatitis, 1, MF, random.<br>• Uterus: Atrophy, D.<br>• Ovaries: Atrophy, D, BL.<br>• Spleen: Plasmacytosis, 2, MF.<br>• Skeletal muscles (hind limb, spine, head): Histiocytic interstitial infiltrates, 2, MF.<br>• Eyes: Mononuclear and neutrophilic keratitis, 2, MF, UL.<br>• Harderian gland: Histiocytic adenitis with acinar degeneration and atrophy, 3, MF, UL.<br>• Ears: Hyperkeratosis with intracorneal bacteria, 3, D, BL, external ear. Histiocytic otitis media with acicular clefts and eosinophilic material, 3, D, BL.
M7/<br>BsAb +<br>$^{177}Lu/^{225}Ac$<br>(62.9 MBq/74 kBq	• Bodyweight: 22.691 g<br>• The kidneys are moderately pale. The liver is moderately pale with enhanced reticular pattern. The spleen is mildly enlarged	• Tumor/injection site: No tumor cells; small remnants of an eosinophilic matrix with central mineralization surrounded by neutrophilic and histiocytic infiltrates.<br>• Skin (flank): Acanthosis and hyperkeratosis, 2, MF.<br>• Kidneys: Mononuclear and neutrophilic tubulointerstitial nephritis with giant cells and interstitial fibrosis, 4, MF, cortical > medullary.<br>• Liver: Mononuclear and neutrophilic hepatitis with giant cells and fibrosis, 3, MF, periportal to random. Biliary epithelial hyalinosis, 2, MF.<br>• Spleen: Neutrophilic infiltrates, 2, MF.<br>• Skeletal muscles (hind limb, spine, head): Mononuclear and neutrophilic interstitial infiltrates, 2, MF. Myofiber degeneration, 1, MF.
M8/<br>BsAb +<br>$^{177}Lu/^{225}Ac$<br>(62.9 MBq/74 kBq	• Bodyweight: 25.782 g	• Tumor/injection site: No tumor remnants.<br>• Skin (flank): Acanthosis and hyperkeratosis, 1, MF.<br>• Kidneys: Tubular degeneration, 2, MF, cortical. Tubular mineralization, 1, MF, medullary.<br>• Liver: Mononuclear perivascular/peribiliary infiltrates, 1, MF. Neutrophilic hepatitis, 1, MF, random.
M9/<br>BsAb +<br>$^{177}Lu/^{225}Ac$	• Bodyweight: 21.131 g	• Tumor/injection site: No tumor remnants.<br>• Skin (flank): Acanthosis and hyperkeratosis, 1, MF. Mononuclear mural folliculitis, 1, F.<br>• Kidneys: Tubular degeneration, 3, MF, cortical. Tubular mineralization, 1, MF, medullary.<br>• Liver: Mononuclear perivascular/peribiliary aggregates, 1, MF. Subcapsular mineralization, 1, MF.

(62.9 MBq/74 kBq		
M10/ BsAb + $^{177}Lu/^{225}Ac$ (62.9 MBq/74 kBq	• Bodyweight: 21.187 g<br>• The kidneys are mildly pale.	• Tumor/injection site: No tumor remnants.<br>• Skin (flank): Acanthosis and hyperkeratosis, 2, MF.<br>• Kidneys: Tubular degeneration, 3, MF, cortical. Tubular mineralization, 1, MF, medullary.<br>• Liver: Mononuclear perivascular/peribiliary aggregates, 1, MF.<br>• Skeletal muscles (hind limb, spine, head): Mononuclear and neutrophilic interstitial infiltrates, 2, MF.
N: Normal F:Focal. MF: Multifocal: D: Diffuse. UL: Unilateral: BL: Bilateral. 1: Minimal. 2: Mild. 3: Moderate. 4: Marked		

LS174T Therapy

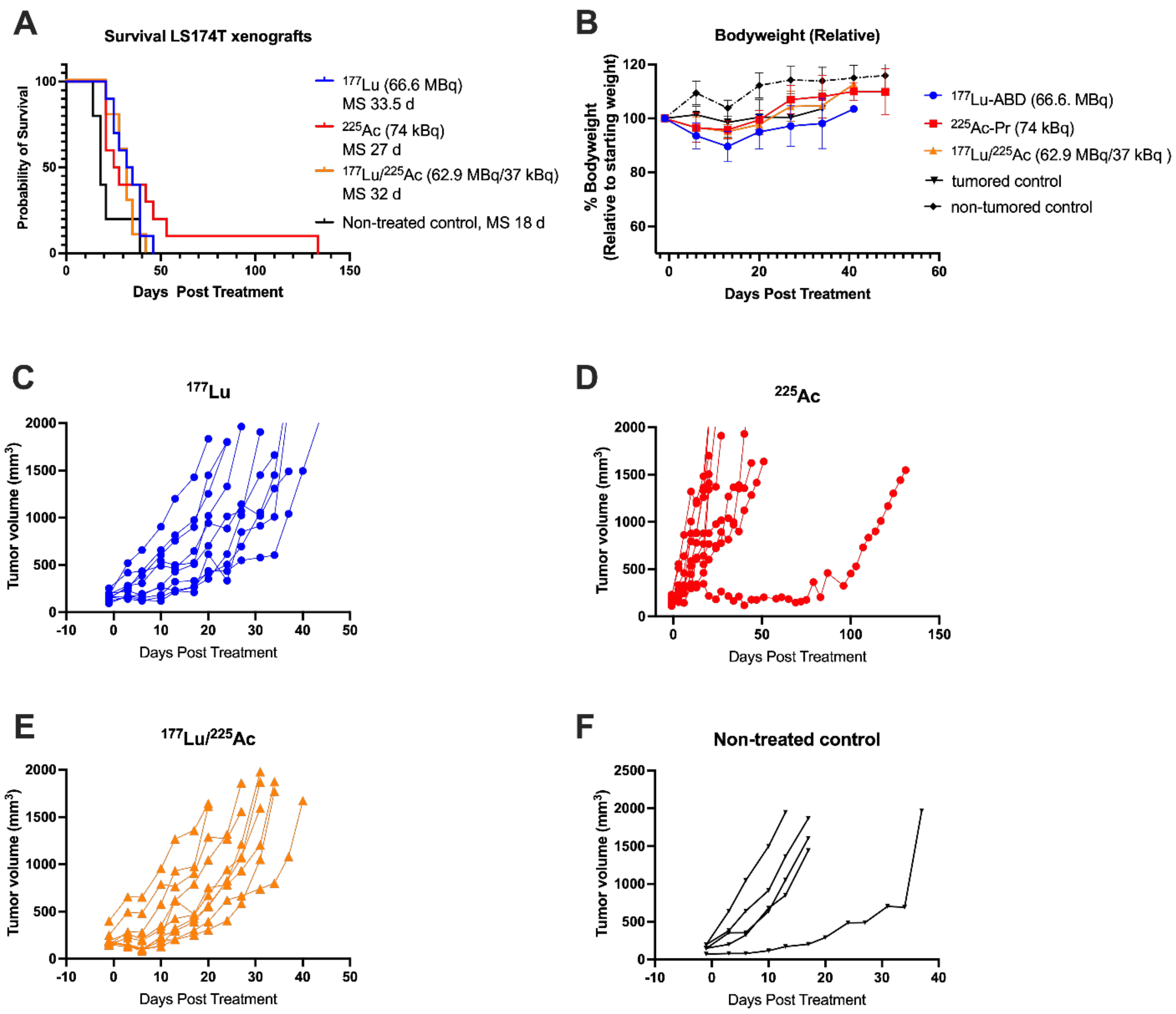

Figure S9: anti-GPA33 DOTA-PRIT therapy in mice with GPA33(+) LS174T xenografts. Mice were treated with 74 kBq ^{225}Ac-DOTA-PRIT, 66.6 MBq ^{177}Lu-DOTA-PRIT of 37 kBq/62.9 MBq of ^{225}Ac/^{177}Lu-DOTA-PRIT. **(A)** Survival. **(B)** Bodyweight relative to starting weight. **(C-F)** Individual tumor volumes.

Toxicity Study

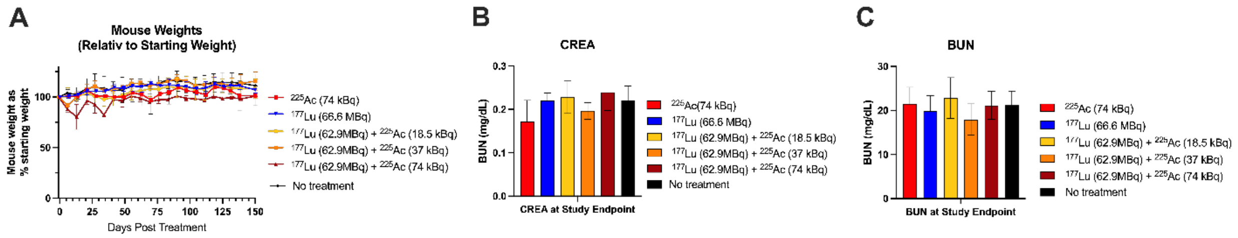

Figure S10: In vivo toxicity in healthy nude mice. Mice were treated with one cycle of 74 kBq ^{225}Ac-DOTA-PRIT, 66.6 MBq ^{177}Lu-DOTA-PRIT, or tandem DOTA-PRIT with 62.9 MBq ^{177}Lu and 18.5-74 kBq ^{225}Ac. **(A)** Mouse weight relative to starting weight. 2-5 animals/data point. Average ± 1SD **(B)** Creatinine (CREA) and **(C)** BUN at study endpoint (150 d). 2-5 animals/data point. Average ± 1SD.

Table S9: Kidney scoring of survivors (Study 1 and Study 2, 150 d post treatment with anti-GPA33 ^{177}Lu /^{225}Ac (62.9 MBq/18.5 kBq)) according to Jaggi et al. (Jaggi et al., 2005). WNL: within normal limits. *data taken from (Jaggi et al., 2005)

**Tubulointestinal Features**	**BsAb + ^{177}Lu /^{225}Ac (62.9 MBq/18.5 kBq)**									**Anti CD33 [^{225}Ac]Ac-mAb***
	**Study 1**					**Study 2**				
Nuclear changes (% of cells)	<1% Karyomegaly <1% Karyorrhexis	<1% Karyomegaly <1% Karyorrhexis	<1% Karyomegaly <1% Karyorrhexis	<40% Karyomegaly <30% Karyorrhexis	WNL	<20% Karyomegaly <10% Karyorrhexis	<1% Karyomegaly <1% Karyorrhexis	<1% Karyomegaly <1% Karyorrhexis	<1% Karyomegaly <1% Karyorrhexis	Moderate, focal karyorrhexis
Cytoplasmic vacuolation (% of cells)	<1%	<1%	<1%	<10%	WNL	<5%	<1%	<1%	<1%	>50
Tubulolysis with collapse (% of tubules)	<1%	<1%	<1%	<70%	WNL	<20%	<1%	<1%	<1%	>50
Loss of brush border (% of tubules)	<1%	<1%	<1%	<70%	WNL	<20%	<1%	<1%	<1%	70
Atrophy (% of tubules)	<1%	<1%	<1%	<70%	WNL	<20%	<1%	<1%	<1%	0
Shrinkage/simplification of tubules (% of tubules)	<1%	<1%	<1%	<70%	WNL	<10%	<1%	<1%	<1%	50
Tubular casts (% of tubules)	<1%	<1%	<1%	<10%	WNL	<10%	<1%	<1%	<1%	Rare
Interstitial inflammation	Minimal, multifocal lymphoplasmacytic infiltrates	Minimal, multifocal lymphoplasmacytic and neutrophilic infiltrates	Minimal, multifocal lymphoplasmacytic infiltrates	Marked, multifocal, lymphoplasmacytic, histiocytic, and giant cell infiltrates	WNL	Minimal, multifocal lymphoplasmacytic and histiocytic infiltrates	Minimal, multifocal lymphoplasmacytic infiltrates	WNL	WNL	0
Interstitial fibrosis	WNL	WNL	WNL	<30%	WNL	<5%	WNL	WNL	WNL	0
Medullary tubules	WNL	WNL	WNL	<10% Casts	WNL	<10% Casts <5% Degeneration	WNL	<1% Casts	<1% Mineralization	Normal

Table S10: Kidney scoring of survivors (Study 1) 150 d post treatment with anti-GPA33 ^{177}Lu or ^{225}Ac) according to Jaggi et al. (Jaggi et al., 2005). WNL: within normal limits. *data taken from (Jaggi et al., 2005)

**Tubulointestinal Features**	**BsAb + 66.6 MBq ^{177}Lu**				**BsAb + 74 kBq ^{225}Ac**								**Anti CD33 [^{225}Ac]Ac-mAb***
Nuclear changes (% of cells)	<1% Karyomegaly <1% Karyorrhexis	<1% Karyomegaly <1% Karyorrhexis	WNL	<1% Karyomegaly <1% Karyorrhexis	<1% Karyomegaly <1% Karyorrhexis	<1% Karyomegaly <1% Karyorrhexis	<1% Karyomegaly <1% Karyorrhexis	WNL	WNL	WNL	WNL	<1% Karyomegaly <1% Karyorrhexis	Moderate, focal karyorrhexis
Cytoplasmic vacuolation (% of cells)	<1%	<1%	WNL	<1%	<1%	<1%	<1%	WNL	WNL	WNL	WNL	<1%	>50
Tubulolysis with collapse (% of tubules)	<1%	<1%	WNL	<1%	<1%	<1%	<1%	WNL	WNL	WNL	WNL	<1%	>50
Loss of brush border (% of tubules)	<1%	<1%	WNL	<1%	<1%	<1%	<1%	WNL	WNL	WNL	WNL	<1%	70
Atrophy (% of tubules)	<1%	<1%	WNL	<1%	<1%	<1%	<1%	WNL	WNL	WNL	WNL	<1%	0
Shrinkage/simplification of tubules (% of tubules)	<1%	<1%	WNL	<1%	<1%	<1%	<1%	WNL	WNL	WNL	WNL	<1%	50
Tubular casts (% of tubules)	<1%	<1%	WNL	<1%	<1%	<1%	<1%	WNL	WNL	WNL	WNL	<1%	Rare
Interstitial inflammation	WNL	Minimal, multifocal lymphoplasmacytic infiltrates	WNL	WNL	Minimal, multifocal lymphoplasmacytic infiltrates	Minimal, multifocal lymphoplasmacytic infiltrates	Minimal, multifocal lymphoplasmacytic and neutrophilic infiltrates	WNL	Minimal, multifocal lymphoplasmacytic infiltrates	WNL	Minimal, multifocal lymphoplasmacytic infiltrates	WNL	0
Interstitial fibrosis	WNL	WNL	WNL	WNL	WNL	WNL	WNL	WNL	WNL	WNL	WNL	WNL	0
Medullary tubules	WNL	WNL	WNL	WNL	WNL	WNL	WNL	WNL	WNL	WNL	WNL	WNL	Normal

Table S11: Kidney scoring of survivors (Study 2) 150 d post treatment with anti-GPA33 ^{177}Lu/ ^{225}Ac-DOTA-PRIT (62.MBq + 37 kBq) according to Jaggi et al. (Jaggi et al., 2005). WNL: within normal limits. *data taken from (Jaggi et al., 2005)

**Tubulointestinal Features**	**BsAb + ^{177}Lu /^{225}Ac (62.9 MBq/37 kBq)**										**Anti CD33 [^{225}Ac]Ac-mAb***
Nuclear changes (% of cells)	<1% Karyomegaly <1% Karyorrhexis	<1% Karyomegaly <1% Karyorrhexis	<10% Karyomegaly <5% Karyorrhexis	<10% Karyomegaly <5% Karyorrhexis	<10% Karyomegaly <5% Karyorrhexis	<5% Karyomegaly <1% Karyorrhexis	<5% Karyomegaly <1% Karyorrhexis	<10% Karyomegaly <5% Karyorrhexis	<1% Karyomegaly <1% Karyorrhexis	<10% Karyomegaly <5% Karyorrhexis	Moderate, focal karyorrhexis
Cytoplasmic vacuolation (% of cells)	<1%	<1%	<1%	<1%	<1%	<1%	<1%	<1%	<1%	<1%	>50
Tubulolysis with collapse (% of tubules)	<1%	<1%	<10%	<10%	<10%	<5%	<5%	<10%	<1%	<10%	>50
Loss of brush border (% of tubules)	<1%	<1%	<10%	<10%	<10%	<5%	<5%	<10%	<1%	<10%	70
Atrophy (% of tubules)	<1%	<1%	<10%	<10%	<10%	<5%	<5%	<10%	<1%	<10%	0
Shrinkage/simplification of tubules (% of tubules)	<1%	<1%	<10%	<10%	<10%	<5%	<5%	<10%	<1%	<10%	50
Tubular casts (% of tubules)	<1%	<1%	<5%	<5%	<5%	<5%	<5%	<5%	<1%	<5%	Rare
Interstitial inflammation	WNL	WNL	WNL	Minimal, multifocal lymphoplasmacytic infiltrates	Minimal, multifocal lymphoplasmacytic infiltrates	WNL	WNL	Mild, multifocal lymphoplasmacytic and neutrophilic infiltrates	WNL	WNL	0
Interstitial fibrosis	WNL	WNL	WNL	<5%	<5%	WNL	WNL	<5%	WNL	<5%	0
Medullary tubules	WNL	<5% Mineralization	<5% Casts	<5% Casts	<1% Casts <1% Mineralization	WNL	<1% Mineralization	<5% Casts	WNL	<5% Casts	Normal

Table S12: Kidney scoring of survivors 150 d post treatment with anti-GPA33 ^{177}Lu/ ^{225}Ac-DOTA-PRIT (62.MBq + 74 kBq) according to Jaggi et al. (Jaggi et al., 2005). WNL: within normal limits. *data taken from (Jaggi et al., 2005)

**Tubulointestinal Features**	**BsAb + ^{177}Lu /^{225}Ac (62.9 MBq/74 kBq)**								**Anti CD33 [^{225}Ac]Ac-mAb***
Nuclear changes (% of cells)	<25% Karyomegaly <15% Karyorrhexis	<20% Karyomegaly <10% Karyorrhexis	<25% Karyomegaly <15% Karyorrhexis	<20% Karyomegaly <10% Karyorrhexis	<40% Karyomegaly <30% Karyorrhexis	<10% Karyomegaly <5% Karyorrhexis	<20% Karyomegaly <10% Karyorrhexis	<20% Karyomegaly <10% Karyorrhexis	Moderate, focal karyorrhexis
Cytoplasmic vacuolation (% of cells)	<5%	<5%	<5%	<5%	<20%	<5%	<5%	<5%	>50
Tubulolysis with collapse (% of tubules)	<40%	<20%	<40%	<30%	<80%	<10%	<20%	<30%	>50
Loss of brush border (% of tubules)	<40%	<30%	<40%	<30%	<80%	<10%	<30%	<30%	70
Atrophy (% of tubules)	<40%	<30%	<40%	<30%	<80%	<10%	<30%	<30%	0
Shrinkage/simplification of tubules (% of tubules)	<30%	<30%	<30%	<30%	<80%	<10%	<30%	<30%	50
Tubular casts (% of tubules)	<10%	<10%	<10%	<10%	<50%	<10%	<10%	<10%	Rare
Interstitial inflammation	Mild, multifocal lymphoplasmacytic and neutrophilic infiltrates	WNL	Mild, multifocal lymphoplasmacytic and neutrophilic infiltrates	WNL	Marked, multifocal, lymphoplasmacytic, histiocytic, neutrophilic, and giant cell infiltrates	WNL	WNL	WNL	0
Interstitial fibrosis	<5%	<1%	<5%	<5%	<40%	WNL	<1%	<5%	0
Medullary tubules	WNL	<1% Mineralization	<1% Mineralization	WNL	<10% Casts <30% Degeneration	<1% Casts <1% Mineralization	<1% Mineralization	<1% Mineralization	Normal

Table S13: Kidney scoring of non-tumored toxicity mice in the toxicity cohort 150 d post treatment with anti-GPA33 ^{177}Lu/ ^{225}Ac-DOTA-PRIT according to Jaggi et al. (Jaggi et al., 2005). WNL: within normal limits. * three mice in the highest dose group were euthanized before the study end date because of significant weight loss. These mice **data taken from (Jaggi et al., 2005)

Tubulointestinal Features	BsAb + ^{177}Lu /^{225}Ac (62.9 MBq/18.5 kBq)					BsAb + ^{177}Lu /^{225}Ac (62.9 MBq/37 kBq)					BsAb + ^{177}Lu /^{225}Ac (62.9 MBq/74 kBq)*		Anti CD33 [^{225}Ac]Ac-mAb*
Nuclear changes (% of cells)	<1% Karyomegaly <1% Karyorrhexis	<1% Karyomegaly <1% Karyorrhexis	<10% Karyomegaly <5% Karyorrhexis	WNL	<1% Karyomegaly <1% Karyorrhexis	<5% Karyomegaly <1% Karyorrhexis	<40% Karyomegaly <30% Karyorrhexis	<10% Karyomegaly <5% Karyorrhexis	<1% Karyomegaly <1% Karyorrhexis	<10% Karyomegaly <5% Karyorrhexis	<20% Karyomegaly <10% Karyorrhexis	<20% Karyomegaly <10% Karyorrhexis	Moderate, focal karyorrhexis
Cytoplasmic vacuolation (% of cells)	<1%	<1%	<1%	WNL	<1%	<1%	<10%	<1%	<1%	<1%	<5%	<5%	>50
Tubulolysis with collapse (% of tubules)	<1%	<1%	<10%	WNL	<1%	<1%	<70%	<10%	<1%	<10%	<20%	<30%	>50
Loss of brush border (% of tubules)	<1%	<1%	<10%	WNL	<1%	<5%	<70%	<10%	<1%	<10%	<30%	<30%	70
Atrophy (% of tubules)	<1%	<1%	<10%	WNL	<1%	<5%	<70%	<10%	<1%	<10%	<30%	<30%	0
Shrinkage/simplification of tubules (% of tubules)	<1%	<1%	<5%	WNL	<1%	<1%	<70%	<10%	<1%	<10%	<20%	<30%	50
Tubular casts (% of tubules)	<1%	<1%	<1%	WNL	<1%	<1%	<50%	<5%	<1%	<5%	<10%	<10%	Rare
Interstitial inflammation	Minimal, multifocal lymphoplasmacytic and neutrophilic infiltrates	Minimal, multifocal lymphoplasmacytic infiltrates	WNL	Minimal, multifocal lymphoplasmacytic infiltrates	WNL	WNL	Marked, multifocal lymphoplasmacytic histiocytic neutrophilic, and giant cell infiltrates	Minimal, multifocal lymphoplasmacytic infiltrates	WNL	WNL	WNL	WNL	0
Interstitial fibrosis	WNL	WNL	WNL	WNL	WNL	WNL	<30%	WNL	WNL	WNL	<5%	<5%	0
Medullary tubules	WNL	<5% Mineralization	<5% Casts	<5% Casts	<1% Casts <1% Mineralization	WNL	<10% Casts	<5% Casts	WNL	<5% Casts <1% Mineralization			Normal

Table S14: Kidney scoring of non-tumored toxicity mice in the toxicity cohort 150d post treatment with anti-GPA33 ^{177}Lu- or ^{225}Ac-DOTA-PRIT according to Jaggi et al. (Jaggi et al., 2005). WNL: within normal limits. * two mice in this group were euthanized early by orders of the veterinary staff. One mouse suffered from a necrotic tail and necrotizing vasculitis; the other mouse had necrotizing dermatitis. Both presentations were considered spontaneous and not treatment related by a veterinary pathologist. **data taken from (Jaggi et al., 2005)

**Tubulointestinal Features**	**BsAb + ^{177}Lu (66.6 MBq)**					**BsAb + ^{225}Ac (74 kBq)***			**Anti CD33 [^{225}Ac]Ac-mAb****
Nuclear changes (% of cells)	<1% Karyomegaly <1% Karyorrhexis	WNL	<10% Karyomegaly <5% Karyorrhexis	WNL	WNL	<1% Karyomegaly <1% Karyorrhexis	<25% Karyomegaly <20% Karyorrhexis	<10% Karyomegaly <5% Karyorrhexis	Moderate, focal karyorrhexis
Cytoplasmic vacuolation (% of cells)	<1%	WNL	<1%	WNL	WNL	<1%	<5%	<1%	>50
Tubulolysis with collapse (% of tubules)	<1%	WNL	<10%	WNL	WNL	<1%	<40%	<10%	>50
Loss of brush border (% of tubules)	<1%	WNL	<10%	WNL	WNL	<1%	<40%	<10%	70
Atrophy (% of tubules)	<1%	WNL	<10%	WNL	WNL	<1%	<40%	<10%	0
Shrinkage/simplification of tubules (% of tubules)	<1%	WNL	<5%	WNL	WNL	<1%	<20%	<5%	50
Tubular casts (% of tubules)	<1%	WNL	<1%	WNL	WNL	<1%	<10%	<1%	Rare
Interstitial inflammation	Minimal, multifocal lymphoplasmacytic infiltrates	Minimal, multifocal lymphoplasmacytic infiltrates	Minimal, multifocal lymphoplasmacytic infiltrates	Minimal, focal lymphoplasmacytic infiltrates	Minimal, focal lymphoplasmacytic infiltrates	Minimal, multifocal lymphoplasmacytic infiltrates	Mild, multifocal lymphoplasmacytic infiltrates	Mild, multifocal lymphoplasmacytic infiltrates	0
Interstitial fibrosis	WNL	WNL	WNL	WNL	WNL	WNL	<1%	WNL	0
Medullary tubules	WNL	WNL	WNL	WNL	WNL	WNL	WNL	WNL	Normal

Table S15: Full pathology report of mice included in the toxicity studies. Mice were euthanized at study endpoint unless otherwise specified.

Animal ID	Anatomic pathology – Toxicity Mice	
	**Gross Finding(s)**	**Microscopic Finding(s) (All tissues are normal unless otherwise described.)**
M1/<br>BsAb +<br>$^{177}Lu/^{225}Ac$<br>(62.9 MBq/18.5 kBq)	• Bodyweight: 19.305 g	• Lungs: Mononuclear and neutrophilic pneumonitis with perivascular, peribronchiolar, or subpleural aggregates, 3, MF.<br>• Thymus: Thymic cysts, MF.<br>• Kidneys: Tubular degeneration, 1, MF, cortical.<br>Mononuclear and neutrophilic interstitial infiltrates, 1, MF, pelvic.<br>• Liver: Mononuclear and neutrophilic perivascular/peribiliary aggregates, 2, MF.<br>• Salivary glands: Mononuclear and neutrophilic perivascular to interstitial aggregates, 2, MF.<br>• Urinary bladder: Mononuclear perivascular aggregates, 1, MF.<br>• Uterus: Pyogranulomatous myometritis and peritonitis, 4, MF.<br>• Vagina: Mononuclear and neutrophilic infiltrates, 2, MF, perigenital musculature/interstitium.<br>• Oviducts: Pyogranulomatous salpingitis and peritonitis, 4, MF.<br>• Pancreas: Pyogranulomatous pancreatitis, 4, MF.<br>• Trachea: Mononuclear and neutrophilic tracheitis with submucosal gland mineralization, 3, MF.<br>• Thyroid: Mononuclear interstitial infiltrates, 2, MF.<br>• Skin (flank): Acanthosis and hyperkeratosis, 2, MF.<br>Mononuclear and neutrophilic dermatitis, folliculitis, and sebaceous adenitis, 2, MF.<br>Pyogranulomatous panniculitis with giant cells, 3, MF.<br>• Bone marrow (femur, tibia, sternum, vertebrae): Myeloid hyperplasia, 3, MF.<br>• Stifle joint: Degenerative joint disease, 3, F.<br>• Skeletal muscles (hind limb, spine, head): Mononuclear myositis, 3, MF.<br>• Eyes: Neutrophilic keratitis with neovascularization and ulceration (UL), 2-4, MF, BL.<br>• Harderian gland: Acinar degeneration and porphyrin deposits, 2, MF, BL.<br>• Ears: Mononuclear and neutrophilic otitis media, 2, MF, BL.<br>Neutrophilic otitis externa with hyperkeratosis, 2, MF, UL.<br>• Other: Mesentery: pyogranulomatous peritonitis, 3-4, MF (slides 4, 5).
M2/<br>BsAb +<br>$^{177}Lu/^{225}Ac$<br>(62.9 MBq/18.5 kBq)	• Bodyweight: 22.098 g<br>• The right axillary lymph node is cystic.	• Lungs: Mononuclear perivascular, peribronchiolar, or subpleural aggregates, 4, MF.<br>• Thymus: Thymic cysts, MF.<br>• Kidneys: Tubular degeneration, 1, MF, cortical.<br>Mononuclear interstitial infiltrates, 1, MF, cortical.<br>• Liver: Mononuclear perivascular/peribiliary infiltrates, 1, MF.<br>• Submandibular lymph node: Medullary plasmacytosis, 3, D.

		Lymphoid follicular hyperplasia, 3, MF.<br>• Uterus: Atrophy, D.<br>• Spleen: Plasmacytosis, 3, MF.<br>• Skin (flank): Acanthosis and hyperkeratosis, 2, MF.<br>Mononuclear and neutrophilic dermatitis, 1, MF.<br>• Nerves (hind limb, spine, head): Mononuclear and neutrophilic ganglioneuritis (DRG) with axonal degeneration, 3, MF.<br>• Oral cavity: Neutrophilic periodontitis, 1, MF.<br>• Harderian gland: Mononuclear and neutrophilic adenitis, with acinar degeneration, atrophy, porphyrin deposits, and interstitial fibrosis, 4, MF, UL.
M3/<br>BsAb +<br>^{177}Lu/^{225}Ac<br>(62.9 MBq/18.5 kBq	• Bodyweight: 23.047 g<br>• The spleen, submandibular, mesenteric, and axillary lymph nodes are mildly enlarged. The skin on the dorsal aspect of the left hip has a small ulcer.	• Lungs: Mononuclear perivascular, peribronchiolar, or subpleural aggregates, 4, MF.<br>• Thymus: Thymic cysts, MF.<br>• Kidneys: Tubular degeneration, 2, MF, cortical.<br>• Liver: Mononuclear perivascular/peribiliary infiltrates, 1, MF.<br>• Mesenteric lymph node: Medullary plasmacytosis, 3, MF. Lymphoid follicular hyperplasia, 3, MF.<br>• Submandibular lymph node: Medullary plasmacytosis, 3, D. Lymphoid follicular hyperplasia, 2, MF.<br>• Urinary bladder: Pyogranulomatous mural cystitis, 3, F. Mononuclear perivascular aggregates, 1, MF.<br>• Uterus: Atrophy, D.<br>• Ovaries: Atrophy, D, UL (one ovary available).<br>• Oviducts: Epithelial vacuolation, 3, MF.<br>• Spleen: Plasmacytosis, 4, MF. Lymphoid follicular hyperplasia, 3, MF.<br>• Adrenals: Subcapsular spindle cell hyperplasia, 2, MF, UL.<br>• Trachea: Mononuclear interstitial infiltrates, 1, MF, lamina propria.<br>• Thyroid: Mononuclear thyroiditis with follicular degeneration, 4, MF.<br>• Skin (flank): Acanthosis and hyperkeratosis, 2, MF. Mononuclear infiltrates, 1, MF.<br>• Skeletal muscles (hind limb, spine, head): Mononuclear and neutrophilic myositis, 1, MF.<br>• Spinal cord: Epidermoid cyst, F.<br>• Nasal cavity: Neutrophilic rhinitis, 3, MF, BL.<br>• Epithelial hyalinosis, 4, D, BL.<br>• Eyes: Mononuclear and neutrophilic endophthalmitis with lens and retinal degeneration, 2, MF, BL.<br>• Harderian gland: Mononuclear and neutrophilic adenitis, with acinar atrophy and interstitial fibrosis, 4, D, UL. Mononuclear and neutrophilic adenitis, with acinar degeneration, 2, MF, UL.<br>• Other: Vasculature: pyogranulomatous vasculitis, 3, F. Mesentery: mononuclear and neutrophilic peritonitis, 2, MF
M4/	• Bodyweight 22.636 g	• Lungs: Mononuclear perivascular, peribronchiolar, or subpleural aggregates, 2, MF.<br>• Thymus: Thymic cysts, MF.

BsAb + $^{177}Lu/^{225}Ac$ (62.9 MBq/18.5 kBq	• The submandibular, mesenteric, and axillary lymph nodes are mildly enlarged.	• Kidneys: Mononuclear interstitial infiltrates, 1, MF.<br>• Liver: Mononuclear perivascular/peribiliary infiltrates, 1, MF.<br>• Mesenteric lymph node: Medullary plasmacytosis, 3, MF. Lymphoid follicular hyperplasia, 2, MF.<br>• Salivary glands: Mononuclear perivascular to interstitial aggregates, 1, MF.<br>• Submandibular lymph node: Medullary plasmacytosis, 3, MF. Lymphoid follicular hyperplasia, 3, MF.<br>• Urinary bladder: Mononuclear interstitial aggregate, 1, F.<br>• Uterus: Atrophy, D.<br>• Ovaries: Atrophy, D, BL.<br>• Oviducts: Epithelial vacuolation, 2, MF.<br>• Adrenals: Accessory adrenocortical nodules, MF, UL. Subcapsular spindle cell hyperplasia, 2, MF, BL.<br>• Trachea: Mononuclear interstitial infiltrates, 1, MF, lamina propria.<br>• Thyroid: Mononuclear thyroiditis with follicular degeneration, 4, MF.<br>• Spinal cord: Epidermoid cyst, F.<br>• Eyes: Mononuclear and neutrophilic endophthalmitis with lens and retinal degeneration, 2, MF, BL.<br>• Harderian gland: Porphyrin deposits, 1, MF, BL.
M5/ BsAb + $^{177}Lu/^{225}Ac$ (62.9 MBq/18.5 kBq	• Bodyweight: 20.325 g	• Lungs: Mononuclear perivascular, peribronchiolar, or subpleural infiltrates, 1, MF.<br>• Thymus: Thymic cysts, MF.<br>• Kidneys: Tubular degeneration, 1, MF, cortical.<br>• Liver: Mononuclear perivascular/peribiliary infiltrates, 1, MF.<br>• Mesenteric lymph node: Medullary plasmacytosis, 2, MF. Lymphoid follicular hyperplasia, 2, MF.<br>• Salivary glands: Mononuclear perivascular aggregate, 1, F.<br>• Submandibular lymph node: Medullary plasmacytosis, 2, MF. Lymphoid follicular hyperplasia, 2, MF.<br>• Urinary bladder: Mononuclear perivascular aggregate, 1, F.<br>• Uterus: Atrophy, D.<br>• Ovaries: Atrophy, D, UL (one ovary available).<br>• Oviducts: Epithelial vacuolation, 3, MF.<br>• Spleen: Plasmacytosis, 2, MF.<br>• Adrenals: Subcapsular spindle cell hyperplasia, 2, MF, UL.<br>• Thyroid: Follicular ectasia, 2, MF.<br>• Skin (flank): Acanthosis and hyperkeratosis, 2, MF. Mononuclear and neutrophilic dermatitis and mural folliculitis, 3, MF.<br>• Skeletal muscles (hind limb, spine, head): Mononuclear and neutrophilic myositis, 1, MF.<br>• Harderian gland: Mononuclear and neutrophilic adenitis with acinar degeneration, 3, MF, BL.<br>• Ears: Mononuclear and neutrophilic otitis externa with hyperkeratosis, 3, MF, BL.<br>• Other: Mesentery: pyogranulomatous peritonitis, 3, MF (slide 3).

M1/<br>BsAb +<br>$^{177}Lu/^{225}Ac$<br>(62.9 MBq/37<br>kBq	• Bodyweight: 21.398 g	• Lungs: Mononuclear and neutrophilic, perivascular, peribronchiolar, or subpleural infiltrates, 1, MF. Vascular mineralization, 1, F.<br>• Thymus: Thymic cysts, MF.<br>• Kidneys: Tubular degeneration, 1, MF, cortical.<br>• Liver: Mononuclear perivascular/peribiliary infiltrates, 1, MF. Neutrophilic infiltrates, 1, F, midzonal.<br>• Stomach: Mononuclear and neutrophilic gastritis with epithelial hyperplasia and hyperkeratosis, 3, D, nonglandular.<br>• Duodenum, jejunum, ileum: Mononuclear and neutrophilic enteritis, 2, MF.<br>• Cecum: Mononuclear and neutrophilic typhlitis, 3, MF.<br>• Colon: Mononuclear and neutrophilic colitis, 3, MF.<br>• Submandibular lymph node: Medullary plasmacytosis, 3, MF.<br>• Ovaries: Atrophy, D, BL.<br>• Oviducts: Epithelial vacuolation, 2, MF.<br>• Adrenals: Accessory adrenocortical nodule, F, UL. Subcapsular spindle cell hyperplasia, 2, MF, BL.<br>• Skin (flank, perigenital, head): Acanthosis and hyperkeratosis, 1, MF. Mononuclear dermatitis dermal fibrosis, 2, MF.<br>• Harderian gland: Acinar degeneration, atrophy, and interstitial fibrosis, with mononuclear interstitial infiltrates, 2, MF, BL.
M2/<br>BsAb +<br>$^{177}Lu/^{225}Ac$<br>(62.9 MBq/37<br>kBq	• Bodyweight: 22.271 g<br>• The kidneys are moderately pale. The left ovary is cystic and enlarged at 1.0 x 0.9 x 0.6 cm.	• Lungs: Mononuclear perivascular, peribronchiolar, or subpleural infiltrates, 2, MF.<br>• Thymus: Thymic cysts, MF.<br>• Kidneys: Mononuclear and neutrophilic tubulointerstitial nephritis with giant cells, 4, MF, cortical.<br>• Liver: Mononuclear perivascular/peribiliary infiltrates, 1, MF.<br>• Salivary glands: Mononuclear perivascular to interstitial aggregates, 2, MF.<br>• Submandibular lymph node: Medullary plasmacytosis, 3, MF. Lymphoid follicular hyperplasia, 2, MF.<br>• Urinary bladder: Mononuclear perivascular infiltrates, 1, F.<br>• Uterus: Atrophy, D.<br>• Ovaries: Atrophy, D, BL. Hemorrhagic cyst, UL.<br>• Adrenals: Subcapsular spindle cell hyperplasia, 2, MF, BL.<br>• Skin (flank, perigenital, head): Acanthosis and hyperkeratosis, 1, MF.<br>• Harderian gland: Pyogranulomatous adenitis, with acinar degeneration, atrophy, porphyrin deposits, and fibrosis, 3, MF, UL.
M3/<br>BsAb +<br>$^{177}Lu/^{225}Ac$<br>(62.9 MBq/37<br>kBq	• Bodyweight: 23.746 g	• Lungs: Mononuclear perivascular, peribronchiolar, or subpleural infiltrates, 1, MF.<br>• Thymus: Thymic cysts, MF.<br>• Kidneys: Tubular degeneration, 2, MF, cortical. Mononuclear interstitial infiltrates, 1, F, pelvic.<br>• Liver: Mononuclear perivascular/peribiliary infiltrates, 2, MF. Neutrophilic infiltrates, 1, MF, random.<br>• Urinary bladder: Mononuclear perivascular infiltrates, 1, MF.

		• Uterus: Atrophy, D.<br>• Ovaries: Atrophy, D, BL.<br>• Oviducts: Epithelial vacuolation, 2, MF.<br>• Adrenals: Accessory adrenocortical nodule, F, UL.<br>• Skin (flank, perigenital, head): Acanthosis and hyperkeratosis, 2, MF.<br>• Bone marrow (femur, tibia, sternum, vertebrae): Fibro-osseous lesion, 2, F, vertebral.<br>• Skeletal muscles (hind limb, spine, head): Mononuclear and neutrophilic interstitial infiltrates, 1, MF.<br>• Eyes: Pyogranulomatous blepharitis, 2, M, UL.<br>• Harderian gland: Mononuclear adenitis, with acinar degeneration, atrophy, and fibrosis, 3, MF, UL.
M4/ BsAb + ^{177}Lu/^{225}Ac (62.9 MBq/37 kBq	• Bodyweight 20.356 g	• Lungs: Mononuclear and neutrophilic, perivascular, peribronchiolar, or subpleural infiltrates, 1, MF.<br>• Thymus: Thymic cysts with neutrophilic and histiocytic debris, MF.<br>• Kidneys: Tubular degeneration, 1, MF, cortical.<br>• Liver: Pyogranulomatous hepatitis, 1, MF, random.<br>• Uterus: Atrophy, D.<br>• Ovaries: Atrophy, D, BL.<br>• Oviducts: Epithelial vacuolation, 2, MF.<br>• Adrenals: Accessory adrenocortical nodule, F, UL. Subcapsular spindle cell hyperplasia, 1, MF, BL.<br>• Skin (flank, perigenital, head): Acanthosis and hyperkeratosis, 2, MF. Pyogranulomatous dermatitis and panniculitis, 3, MF.<br>• Eyes: Pyogranulomatous blepharitis, 3, D, BL.<br>• Ears: Hyperkeratosis with intracorneal bacteria, 3, D, UL, external ear.
M5/ BsAb + ^{177}Lu/^{225}Ac (62.9 MBq/37 kBq	• Bodyweight 25.876 g	• Lungs: Mononuclear perivascular, peribronchiolar, or subpleural infiltrates, 2, MF.<br>• Thymus: Thymic cysts, MF.<br>• Kidneys: Tubular degeneration, 2, MF, cortical. Tubular mineralization, 1, MF, medullary.<br>• Liver: Mononuclear perivascular/peribiliary infiltrates, 1, MF.<br>• Submandibular lymph node: Medullary plasmacytosis, 2, MF. Neutrophilic infiltrates, 2, MF.<br>• Ovaries: Atrophy, D, BL.<br>• Adrenals: Subcapsular spindle cell hyperplasia, 2, MF, UL.<br>• Trachea: Mononuclear interstitial infiltrates, 1, MF, lamina propria.<br>• Skin (flank, perigenital, head): Acanthosis and hyperkeratosis, 1, MF. Mononuclear and neutrophilic panniculitis and myositis, 2, MF, perigenital.<br>• Skeletal muscles (hind limb, spine, head): Mononuclear and neutrophilic interstitial infiltrates, 1, MF<br>• Eyes: Pyogranulomatous blepharitis, 2, M, UL.<br>• Harderian gland: Porphyrin deposits, 2, MF, BL.
M1/	• Bodyweight: 23.292 g	• Lungs: Mononuclear perivascular, peribronchiolar, or subpleural infiltrates, 2, MF.<br>• Thymus: Thymic cysts, MF.

BsAb + $^{177}Lu/^{225}Ac$ (62.9 MBq/74 kBq	• The kidneys are mildly pale.	• Kidneys: Tubular degeneration, 3, MF, cortical > medullary. Tubular mineralization, 1, MF, medullary.<br>• Liver: Mononuclear perivascular/peribiliary infiltrates, 1, MF.<br>• Urinary bladder: Mononuclear perivascular infiltrates, 1, MF.<br>• Uterus: Atrophy, D.<br>• Ovaries: Atrophy, D, BL.<br>• Oviducts: Epithelial vacuolation, 2, MF.<br>• Adrenals: Subcapsular spindle cell hyperplasia, 1, MF, BL.<br>• Skin (flank, perigenital, head): Acanthosis and hyperkeratosis, 2, MF. Pyogranulomatous epidermitis and panniculitis, 2, MF.<br>• Eyes: Pyogranulomatous blepharitis, 3, MF, BL.<br>• Harderian gland: Histiocytic adenitis with acinar degeneration, atrophy, porphyrin deposits, and fibrosis, 3, MF, UL.
M2/ BsAb + $^{177}Lu/^{225}Ac$ (62.9 MBq/74 kBq	• Bodyweight: 15.9 g	Mouse was found dead in the cage on day 14. Body was moderately autolyzed.<br><br>• Heart: Cardiomyocyte degeneration and hemorrhage, 2, MF, right ventricle and septum. Bacterial overgrowth, 3, MF.<br>• Lungs: Bacterial overgrowth, 4, D.<br>• Kidneys: Tubular degeneration, 1, MF, cortical. Bacterial overgrowth, 3, MF.<br>• Liver: Mononuclear perivascular/peribiliary aggregates, 1, MF. Bacterial overgrowth, 3, MF.<br>• Uterus: Atrophy, D.<br>• Ovaries: Atrophy, D, BL.<br>• Spleen: White and red pulp hypocellularity, 4, D.<br>• Bone marrow (femur, tibia, sternum, vertebrae): Hypocellularity (all lineages), 4, D.<br>• Spinal cord: Hemorrhage, 2, MF.<br>• Harderian gland: Acinar degeneration, atrophy, porphyrin deposits, and edema, 4, MF, UL. Bacterial overgrowth, 4, MF.<br>• Brain: Hemorrhage, 3, MF. Bacterial overgrowth, 3, MF.<br>• Other: Vasculature: bacteremia (postmortem overgrowth), 4, MF.
M3/ BsAb + $^{177}Lu/^{225}Ac$ (62.9 MBq/74 kBq	• Bodyweight 13.138 g<br>• The skin throughout the body has multiple red pinpoints.	Mouse was euthanized on day 14 due to weight loss >20%<br><br>• Heart: Cardiomyocyte degeneration and hemorrhage, 2, MF, right ventricle and septum.<br>• Thymus: Thymic cysts, MF.<br>• Kidneys: Tubular mineralization, 1, MF, medullary.<br>• Liver: Mononuclear perivascular/peribiliary aggregates, 1, MF. Decreased glycogen deposits, 2, D, periportal.<br>• Uterus: Atrophy, D.

		• Ovaries: Atrophy, D, BL.<br>• Oviducts: Epithelial vacuolation, 3, MF.<br>• Spleen: White and red pulp hypocellularity, 4, D.<br>• Adrenals: Accessory adrenocortical nodule, F, UL.<br>• Skin (flank, perigenital, head): Acanthosis and hyperkeratosis with intracorneal bacterial colonies (C. bovis), 3, MF.<br>• Bone marrow (femur, tibia, sternum, vertebrae): Hypocellularity (all lineages), 4, D.<br>• Spinal cord: Hemorrhage, 3, F.<br>• Harderian gland: Histiocytic adenitis with acinar degeneration, atrophy, porphyrin deposits, edema, and interstitial fibrosis, 3, MF, BL.<br>• Brain: Hemorrhage, 3, MF.
M4/<br>BsAb +<br>^{177}Lu/^{225}Ac<br>(62.9 MBq/74 kBq	• Bodyweight: 15.1 g	Mouse was euthanized on day 35 due to weight loss >20%<br><br>• Lungs: Vascular fibrinoid necrosis with alveolar edema, hemorrhage, and fibrin deposits, 3, MF.<br>• Kidneys: Tubular degeneration, 1, MF, cortical.<br>• Liver: Decreased glycogen deposits, 2, D, periportal to midzonal. Microvesicular lipidosis, 2, MF, centrilobular to midzonal. Sinusoidal dilation, 2, MF, centrilobular to midzonal.<br>• Submandibular lymph node: Sinus histiocytosis with pigment-laden macrophages, 3, MF.<br>• Uterus: Atrophy, D.<br>• Ovaries: Atrophy, D, BL.<br>• Oviducts: Epithelial vacuolation, 2, MF.<br>• Spleen: White and red pulp hypocellularity, 4, D. Pigment-laden macrophage infiltrates, 3, MF.<br>• Skin (flank, perigenital, head): Acanthosis and hyperkeratosis, 3, MF.<br>• Bone marrow (femur, tibia, sternum, vertebrae): Hypocellularity (all lineages), 4, D. Necrosis, 4, MF.<br>• Spinal cord: Hemorrhage, 2, F.<br>• Harderian gland: Coagulative necrosis, 4, F, UL.<br>• Brain: Hemorrhage, 2, MF.
M5/<br>BsAb +<br>^{177}Lu/^{225}Ac<br>(62.9 MBq/74 kBq	• Bodyweight: 20.431 g	• Lungs: Mononuclear perivascular, peribronchiolar, or subpleural infiltrates, 1, MF.<br>• Kidneys: Tubular degeneration, 3, MF, cortical > medullary. Tubular mineralization, 2, MF, medullary.<br>• Liver: Mononuclear perivascular/peribiliary aggregates, 1, MF.<br>• Uterus: Atrophy, D.<br>• Ovaries: Atrophy, D, BL.<br>• Oviducts: Epithelial vacuolation, 2, MF.<br>• Skin (flank, perigenital, head): Acanthosis and hyperkeratosis, 2, MF. Mononuclear dermatitis, 2, MF, perigenital

		• Harderian gland: Pyogranulomatous adenitis, with acinar degeneration, atrophy, porphyrin deposits, and fibrosis, 4 MF, UL.
M1/<br>BsAb + ^{177}Lu<br>(66.6 MBq)	• Bodyweight: 22.979 g<br>• The right axillary lymph node is cystic.	• Lungs: Mononuclear perivascular, peribronchiolar, or subpleural infiltrates, 1, MF.<br>• Thymus: Thymic cysts, MF.<br>• Kidneys: Tubular degeneration, 1, MF, cortical. Mononuclear interstitial infiltrates, 1, MF, pelvic.<br>• Liver: Mononuclear perivascular/peribiliary infiltrates, 1, MF.<br>• Ovaries: Atrophy, D, UL (one ovary available).<br>• Oviducts: Epithelial vacuolation, 3, MF.<br>• Adrenals: Accessory adrenocortical nodule, F, UL. Subcapsular spindle cell hyperplasia, 2, MF, BL.<br>• Skin (flank): Acanthosis and hyperkeratosis, 2, MF. Mononuclear and neutrophilic dermatitis and mural folliculitis, 3, MF.<br>• Harderian gland: Histiocytic adenitis with acinar degeneration, atrophy, and interstitial fibrosis, 2, MF, UL.<br>• Ears: Neutrophilic and histiocytic otitis externa, 3, F, UL.
M2/<br>BsAb + ^{177}Lu<br>(66.6 MBq)	• Bodyweight: 22.976 g<br>• The right axillary lymph node is cystic.	• Lungs: Mononuclear perivascular, peribronchiolar, or subpleural infiltrates, 1, MF.<br>• Kidneys: Mononuclear interstitial infiltrates, 1, MF, cortical.<br>• Liver: Mononuclear perivascular/peribiliary infiltrates, 1, MF.<br>• Urinary bladder: Mononuclear and neutrophilic perivascular aggregate, 2, F.<br>• Uterus: Atrophy, D.<br>• Oviducts: Epithelial vacuolation, 3, MF.<br>• Adrenals: Accessory adrenocortical nodules, MF, UL. Subcapsular spindle cell hyperplasia, 2, MF, BL.<br>• Thyroid: Mononuclear thyroiditis with follicular degeneration and ectasia, 4, MF.<br>• Skin (flank): Acanthosis and hyperkeratosis, 2, MF. Dermal fibrosis, 3, MF.<br>• Nasal cavity: Neutrophilic rhinitis, 3, MF, BL. Epithelial hyalinosis, 4, D, BL.<br>• Eyes: Mononuclear and neutrophilic endophthalmitis with lens degeneration and retinal atrophy, 3, D, BL.<br>• Harderian gland: Mononuclear and neutrophilic adenitis, with acinar degeneration, atrophy, porphyrin deposits, and interstitial fibrosis, 4, MF, BL.
M3/<br>BsAb + ^{177}Lu<br>(66.6 MBq)	• Bodyweight 25.805 g<br>• The submandibular, mesenteric, and axillary lymph nodes are mildly enlarged.	• Lungs: Mononuclear perivascular, peribronchiolar, or subpleural infiltrates, 1, MF.<br>• Kidneys: Tubular degeneration, 2, MF, cortical. Mononuclear interstitial infiltrates, 1, MF, cortical and pelvic.<br>• Liver: Mononuclear perivascular/peribiliary infiltrates, 1, MF.<br>• Mesenteric lymph node: Medullary plasmacytosis, 2, MF. Lymphoid follicular hyperplasia, 2, MF.<br>• Submandibular lymph node: Medullary plasmacytosis, 2, MF. Lymphoid follicular hyperplasia, 2, MF.<br>• Urinary bladder: Mononuclear perivascular aggregate, 1, F.<br>• Vagina: Mononuclear infiltrates, 2, MF, perigenital musculature/interstitium.

		• Ovaries: Atrophy, D, UL (one ovary available).<br>• Spleen: Plasmacytosis, 2, MF.<br>• Adrenals: Accessory adrenocortical nodule, F, UL. Subcapsular spindle cell hyperplasia, 3, MF, UL.<br>• Skin (flank): Acanthosis and hyperkeratosis, 2, MF. Mononuclear and neutrophilic dermatitis, 2, MF.<br>• Eyes: Mononuclear and neutrophilic endophthalmitis with lens degeneration and retinal atrophy, 3, D, UL.<br>• Harderian gland: Histiocytic adenitis, with acinar degeneration, atrophy, and interstitial fibrosis, 4, D, UL. Mononuclear interstitial aggregates, with acinar degeneration, 2, MF, UL.
M4/<br>BsAb + ^{177}Lu<br>(66.6 MBq)	• Bodyweight: 22.316 g	• Lungs: Mononuclear perivascular, peribronchiolar, or subpleural infiltrates, 1, MF.<br>• Thymus: Thymic cysts, MF.<br>• Kidneys: Mononuclear interstitial infiltrates, 1, F, pelvic.<br>• Liver: Mononuclear perivascular/peribiliary infiltrates, 1, MF.<br>• Gallbladder: Mononuclear interstitial aggregate, 1, F.<br>• Salivary glands: Mononuclear perivascular to interstitial aggregates, 2, MF.<br>• Submandibular lymph node: Medullary plasmacytosis, 2, MF. Lymphoid follicular hyperplasia, 2, MF.<br>• Oviducts: Epithelial vacuolation, 2, MF.<br>• Adrenals: Subcapsular spindle cell hyperplasia, 2, MF, BL.<br>• Trachea: Mononuclear interstitial infiltrates, 1, MF, lamina propria.<br>• Thyroid: Mononuclear thyroiditis with follicular degeneration and ectasia, 4, MF.<br>• Skin (flank): Acanthosis and hyperkeratosis, 2, MF. Mononuclear dermatitis and mural folliculitis, 1, MF.<br>• Spinal cord: Epidermoid cyst, F.<br>• Harderian gland: Porphyrin deposits, 2, MF, BL.
M5/<br>BsAb + ^{177}Lu<br>(66.6 MBq)	• Bodyweight: 22.977 g	• Lungs: Mononuclear perivascular, peribronchiolar, or subpleural infiltrates, 1, MF.<br>• Thymus: Thymic cysts, MF.<br>• Kidneys: Mononuclear interstitial infiltrates, 1, F, pelvic.<br>• Liver: Mononuclear perivascular/peribiliary infiltrates, 1, MF.<br>• Salivary glands: Mononuclear perivascular to interstitial aggregates, 2, MF.<br>• Urinary bladder: Mononuclear perivascular aggregates, 1, MF.<br>• Uterus: Atrophy, D.<br>• Ovaries: Atrophy, D, UL.<br>• Oviducts: Epithelial vacuolation, 2, MF.<br>• Pancreas: Mononuclear perivascular to interstitial aggregates, 2, MF.<br>• Trachea: Mononuclear interstitial infiltrates, 1, F, lamina propria.<br>• Harderian gland: Porphyrin deposits, 2, MF, BL.<br>• Pituitary: Mononuclear infiltrates, 3, MF.

M1/<br>BsAb + ^{225}Ac<br>(74 kBq)	• Bodyweight: 23.308 g<br>• The spleen, submandibular, mesenteric, and axillary lymph nodes are mildly enlarged. The skin on the dorsal aspect of the left hip has a small ulcer.	• Heart: Mononuclear and neutrophilic myocarditis, 3, MF, left ventricle.<br>• Lungs: Mononuclear and neutrophilic pneumonitis and vasculitis with perivascular, peribronchiolar to interstitial, or subpleural aggregates, 3, MF.<br>• Kidneys: Tubular degeneration, 1, MF, cortical. Mononuclear perivascular to interstitial infiltrates, 1, MF, cortical and pelvic.<br>• Liver: Mononuclear perivascular/peribiliary infiltrates, 1, MF.<br>• Mesenteric lymph node: Medullary plasmacytosis, 3, D. Lymphoid follicular hyperplasia, 2, MF.<br>• Salivary glands: Mononuclear perivascular to interstitial aggregates, 2, MF.<br>• Submandibular lymph node: Medullary plasmacytosis, 3, D. Lymphoid follicular hyperplasia, 2, MF.<br>• Urinary bladder: Pyogranulomatous mural cystitis, 4, MF. Mononuclear perivascular aggregates, 1, MF.<br>• Uterus: Atrophy, D.<br>• Vagina: Mononuclear and neutrophilic infiltrates, 2, MF, perigenital musculature/interstitium.<br>• Ovaries: Pigment-laden macrophage infiltrates, 3, MF.<br>• Oviducts: Epithelial vacuolation, 3, MF.<br>• Spleen: Plasmacytosis, 3, MF. Lymphoid follicular hyperplasia, 3, MF.<br>• Pancreas: Mononuclear perivascular to interstitial aggregates, 2, MF.<br>• Trachea: Mononuclear interstitial infiltrates, 1, MF, lamina propria.<br>• Skin (flank): Acanthosis and hyperkeratosis, 2, MF. Pyogranulomatous panniculitis, 2, F.<br>• Eyes: Mononuclear and neutrophilic endophthalmitis with lens degeneration and retinal atrophy, 3, D, UL.<br>• Harderian gland: Acinar degeneration and porphyrin deposits, 2, MF, BL.<br>• Other: Mesentery: pyogranulomatous peritonitis, 4, MF (slide 5).
M2/<br>BsAb + ^{225}Ac<br>(74 kBq)	• Bodyweight: 15.648 g<br>• The tail is shortened, diffusely purple to black, and the bone is exposed at the distal end (approximately 2 mm circumferentially). There is skin bruising with subcutaneous hemorrhage at the	Mouse was euthanized at day 70. Clinical decline was due to widespread/multisystemic necrotizing vasculitis that was considered not treatment related.<br>• Heart: Cardiomyocyte hypereosinophilia, 2, F.<br>• Lungs: Mononuclear perivascular, peribronchiolar, or subpleural infiltrates, 1, MF. Mononuclear and neutrophilic vasculitis with thrombosis, 2, MF.<br>• Thymus: Thymic cysts, MF.<br>• Kidneys: Tubular degeneration, 1, MF, cortical.<br>• Liver: Mononuclear perivascular/peribiliary aggregates, 1, MF. Decreased glycogen deposits, 2, D, periportal.<br>• Salivary glands: Necrotizing vasculitis with thrombosis and parenchymal infarction, 4, MF.<br>• Submandibular lymph node: Medullary plasmacytosis, 3, MF. Lymphoid follicular hyperplasia, 2, MF. Necrotizing vasculitis with thrombosis and parenchymal infarction, 4, MF.<br>• Uterus: Atrophy, D.<br>• Vagina: Necrotizing vasculitis with thrombosis, 4, MF, perigenital tissues.

	right ventral cervical region, left mandible, left axilla, and behind the left pinna. The thoracic and abdominal cavities have approximately 0.7 ml and 0.1 ml of an opaque tan effusion.	• Ovaries: Atrophy, D, BL.<br>• Adrenals: Accessory adrenocortical nodules, MF, UL.<br>• Skin (flank): Acanthosis and hyperkeratosis, 3, MF. Neutrophilic epidermitis and folliculitis, 2, F. Necrotizing vasculitis with thrombosis and parenchymal infarction, 4, MF, tail (slide 10).<br>• Skeletal muscles (hind limb, spine, head): Necrotizing vasculitis with thrombosis, 4, MF, head.<br>• Oral cavity: Necrotizing vasculitis with thrombosis, 4, MF, tongue.<br>• Harderian gland: Histiocytic adenitis with acinar degeneration, atrophy, porphyrin deposits, and interstitial fibrosis, 3, MF, BL. Necrotizing vasculitis with thrombosis and parenchymal infarction, 4, MF.<br>• Other: Vasculature: necrotizing vasculitis with thrombosis, 4, MF. Mesentery/mediastinum: coagulative necrosis and mononuclear infiltrates, 2-3, MF
M3/<br>BsAb + ^{225}Ac<br>(74 kBq)	• Bodyweight: 20.838 g<br>• The spleen is mildly enlarged.	• Lungs: Mononuclear perivascular, peribronchiolar, or subpleural infiltrates, 1, MF.<br>• Thymus: Thymic cysts, MF.<br>• Kidneys: Tubular degeneration, 3, MF, cortical. Mononuclear perivascular to interstitial infiltrates, 2, MF, cortical and pelvic.<br>• Liver: Mononuclear perivascular/peribiliary infiltrates, 1, MF.<br>• Ovaries: Pigment-laden macrophage infiltrates, 3, MF.<br>• Oviducts: Epithelial vacuolation, 3, MF.<br>• Spleen: Lymphoid follicular hyperplasia, 2, MF.<br>• Adrenals: Subcapsular spindle cell hyperplasia, 2, MF, UL.<br>• Bones (femur, tibia, sternum, vertebrae): Joint degeneration, 2, MF, stifle, sternum, vertebrae.<br>• Bone marrow (femur, tibia, sternum, vertebrae): Myeloid hyperplasia, 3, MF. Necrosis, 3, F, tibia.<br>• Stifle joint: Pyogranulomatous tenosynovitis, 3, MF.<br>• Oral cavity: Neutrophilic periodontitis, 2, MF.<br>• Eyes: Mononuclear and neutrophilic endophthalmitis with lens degeneration and retinal atrophy, 3, D, BL.<br>• Harderian gland: Mononuclear and neutrophilic adenitis, with acinar degeneration, atrophy, porphyrin deposits, and interstitial fibrosis, 3, MF, UL.<br>• Ears: Neutrophilic otitis externa with hyperkeratosis and intracorneal bacteria, 3, MF, BL.<br>• Other: Popliteal lymph node and hindlimb adipose tissue: pyogranulomatous lymphadenitis and steatitis, 3, MF.
M4/<br>BsAb + ^{225}Ac<br>(74 kBq)	• Bodyweight: 21.608 g	Mouse was euthanized on day 87 due to necrotizing dermatitis that was considered non-treatment related<br>• Lungs: Mononuclear perivascular, peribronchiolar, or subpleural aggregates, 2, MF. Alveolar histiocytosis, 1, MF.<br>• Thymus: Thymic cysts, MF.

		• Kidneys: Tubular degeneration, 2, MF, cortical. Mononuclear perivascular to interstitial aggregates, 2, MF, cortical and pelvic.<br>• Liver: Mononuclear perivascular/peribiliary infiltrates, 1, MF.<br>• Mesenteric lymph node: Medullary plasmacytosis, 3, D. Lymphoid follicular hyperplasia, 2, MF.<br>• Salivary glands: Mononuclear perivascular aggregate, 2, F.<br>• Submandibular lymph node: Medullary plasmacytosis, 3, D. Lymphoid follicular hyperplasia, 2, MF.<br>• Urinary bladder: Mononuclear and neutrophilic cystitis, 3, MF. Mononuclear perivascular aggregates, 1, MF.<br>• Vagina: Mononuclear and neutrophilic infiltrates, 2, MF, perigenital musculature/interstitium.<br>• Ovaries: Pigment-laden macrophage infiltrates, 3, MF.<br>• Oviducts: Epithelial vacuolation, 3, MF.<br>• Spleen: Plasmacytosis, 2, MF.<br>• Pancreas: Mononuclear and neutrophilic perivascular to interstitial infiltrates, 1, MF.<br>• Adrenals: Accessory adrenocortical nodules, MF, BL. Subcapsular spindle cell hyperplasia, 2, MF, BL.<br>• Trachea: Mononuclear interstitial infiltrates, 1, MF, lamina propria.<br>• Harderian gland: Acinar degeneration and porphyrin deposits, 1, MF, BL.<br>• Ears: Neutrophilic otitis externa, 2, F, UL.<br>• Other: Mesentery: mononuclear and neutrophilic peritonitis, 2, MF. Meibomian gland: pyogranulomatous adenitis, 2, MF, UL. Zymbal's gland: pyogranulomatous adenitis, 2, MF, UL.
M5/<br>BsAb + ^{225}Ac<br>(74 kBq)	• Bodyweight: 20.039 g<br>• Ther are large irregularly shaped areas of skin ulceration on the following areas: right caudal flank around (3.5 cm in length rising up to mid thorax to the level of the right forelimb); left caudal flank (1.5 cm in length); and ventral abdomen (2.0 cm in length). Multiple smaller	• Lungs: Mononuclear perivascular, peribronchiolar, or subpleural infiltrates, 1, MF.<br>• Thymus: Thymic cysts, MF.<br>• Liver: Mononuclear perivascular/peribiliary infiltrates, 1, MF. Increased mitotic activity, 3, MF, random.<br>• Uterus: Atrophy, D.<br>• Ovaries: Atrophy, D, BL.<br>• Spleen: Lymphoid follicular hyperplasia, 2, MF.<br>• Adrenals: Subcapsular spindle cell hyperplasia, 2, MF, UL.<br>• Trachea: Mononuclear interstitial infiltrates, 1, MF, lamina propria.<br>• Skin (flank): Acanthosis and hyperkeratosis, 3, MF. Neutrophilic and mononuclear dermatitis with full-thickness epidermal necrosis, dermal fibrosis, and occasional spongiosis of remnant intact epidermis, 4, MF.<br>• Bone marrow (femur, tibia, sternum, vertebrae): Myeloid hyperplasia, 3, D.<br>• Spinal cord: Epidermoid cyst, F.<br>• Harderian gland: Porphyrin deposits, 1, MF, BL.

	irregularly shaped skin ulcerations are noted near the right and left ankles and left cranial flank. The skin throughout the body, but predominantly on the dorsum, is multifocally thickened and flaky. The spleen is mildly enlarged.	
M1/untreated control	• Bodyweight: 21.681 g	• Heart: Mononuclear interstitial infiltrates, 1, MF, left atrium.<br>• Lungs: Mononuclear perivascular, peribronchiolar, or subpleural aggregates, 2, MF.<br>• Thymus: Thymic cysts, MF.<br>• Kidneys: Mononuclear interstitial infiltrates, 1, F, cortical.<br>• Liver: Mononuclear perivascular/peribiliary infiltrates, 1, MF.<br>• Submandibular lymph node: Medullary plasmacytosis, 1, D.<br>• Vagina: Mononuclear and neutrophilic infiltrates, 2, MF, perigenital musculature/interstitium.<br>• Oviducts: Epithelial vacuolation, 3, MF.<br>• Pancreas: Ductal dilation with luminal inspissated material, 3, MF.<br>• Adrenals: Accessory adrenocortical nodule, F, UL. Subcapsular spindle cell hyperplasia, 1, MF, UL.<br>• Trachea: Mononuclear interstitial infiltrates, 1, F, lamina propria.<br>• Thyroid: Follicular ectasia, 2, MF.<br>• Bones (femur, tibia, sternum, vertebrae): Fibro-osseous lesion, 2, F, vertebral.<br>• Harderian gland: Acinar degeneration and porphyrin deposits, 2, MF, BL.<br>• Ears: Neutrophilic and histiocytic otitis media, 2-3, D, BL.
M2/untreated control	• Bodyweight: 28.933 g	• Lungs: Mononuclear perivascular/peribronchiolar aggregates, 1, MF.<br>• Thymus: Thymic cysts, MF.<br>• Kidneys: Mononuclear perivascular to interstitial aggregates, 2, MF, cortical.<br>• Liver: Mononuclear perivascular/peribiliary infiltrates, 1, MF.<br>• Vagina: Mononuclear and neutrophilic infiltrates with giant cells, 4, MF, submucosal/muscularis.<br>• Adrenals: Subcapsular spindle cell hyperplasia, 1, MF, UL.<br>• Harderian gland: Acinar degeneration and porphyrin deposits, 2, MF, BL. Histiocytic interstitial aggregates, 2, MF, UL.

M3/untreated control	• Bodyweight: 24.312 g<br>• The spleen is mildly enlarged.	• Lungs: Mononuclear perivascular, peribronchiolar, or subpleural infiltrates, 1, MF.<br>• Kidneys: Mononuclear perivascular to interstitial aggregates, 2, MF, cortical and pelvic.<br>• Liver: Mononuclear perivascular/peribiliary infiltrates, 1, MF.<br>• Mesenteric lymph node: Pyogranulomatous lymphadenitis, 2, F.<br>• Salivary glands: Mononuclear perivascular to interstitial aggregates, 1, MF.<br>• Vagina: Mononuclear and neutrophilic infiltrates, 3, MF, perigenital musculature/interstitium.<br>• Spleen: Lymphoid follicular hyperplasia, 2, MF.<br>• Adrenals: Subcapsular spindle cell hyperplasia, 2, MF, UL.<br>• Esophagus: Mononuclear and neutrophilic interstitial infiltrates, 1, MF, muscularis.<br>• Thyroid: Mononuclear interstitial infiltrates, 3, MF.<br>• Stifle joint: Pyogranulomatous tenosynovitis, 3, MF.<br>• Oral cavity: Neutrophilic periodontitis, 2, MF.<br>• Eyes: Mononuclear and neutrophilic adenitis with lens degeneration, 3, D, BL.<br>• Harderian gland: Mononuclear and neutrophilic adenitis, with acinar degeneration, atrophy, porphyrin deposits, and interstitial fibrosis, 4, MF, BL.<br>• Ears: Neutrophilic otitis externa with hyperkeratosis and intracorneal bacteria, 3, MF, BL.<br>• Other: Mesentery: mononuclear and neutrophilic peritonitis, 2, MF
M4/untreated control	• Bodyweight: 20.559 g	• Lungs: Mononuclear perivascular, peribronchiolar, or subpleural infiltrates, 1, MF.<br>• Thymus: Thymic cysts, MF.<br>• Kidneys: Tubular degeneration, 1, MF, cortical. Mononuclear perivascular to interstitial aggregates, 1, MF, cortical.<br>• Liver: Mononuclear perivascular/peribiliary infiltrates, 1, MF.<br>• Salivary glands: Mononuclear perivascular aggregate, 2, F.<br>• Urinary bladder: Mononuclear and neutrophilic cystitis, 3, MF. Mononuclear perivascular aggregates, 1, MF.<br>• Vagina: Mononuclear and neutrophilic infiltrates, 2, MF, submucosal/muscularis.<br>• Pancreas: Mononuclear and neutrophilic perivascular to interstitial infiltrates, 1, MF.<br>• Adrenals: Accessory adrenocortical nodules, MF, BL. Subcapsular spindle cell hyperplasia, 2, MF, BL.<br>• Trachea: Mononuclear interstitial infiltrates, 1, MF, lamina propria.<br>• Skin (flank): Acanthosis and hyperkeratosis, 2, MF. Mononuclear dermatitis and mural folliculitis, 1, MF.<br>• Harderian gland: Acinar degeneration and porphyrin deposits, 2, MF, BL. Mononuclear interstitial aggregates, 2, MF, BL.
M5/untreated control	• Bodyweight: 23.77 g	• Lungs: Mononuclear perivascular, peribronchiolar, or subpleural infiltrates, 1, MF.<br>• Kidneys: Tubular degeneration, 2, MF, cortical. Mononuclear perivascular to interstitial aggregates, 2, MF, cortical.<br>• Liver: Mononuclear perivascular/peribiliary infiltrates, 1, MF.

		• Submandibular lymph node: Lymphatic ectasia, 3, F.<br>• Urinary bladder: Mononuclear perivascular aggregate, 1, F.<br>• Adrenals: Subcapsular spindle cell hyperplasia, 2, MF, UL.<br>• Trachea: Mononuclear interstitial infiltrates, 1, MF, lamina propria.<br>• Skin (flank): Acanthosis and hyperkeratosis, 3, MF. Neutrophilic and mononuclear dermatitis with full-thickness epidermal necrosis, dermal fibrosis, and occasional spongiosis of remnant intact epidermis, 4, MF.<br>• Harderian gland: Acinar degeneration and porphyrin deposits, 1, MF, BL. Mononuclear interstitial aggregates, 2, MF, BL.
M1/ $^{177}Lu/^{225}Ac$ only (62.9 MBq/18.5 kBq)	• Bodyweight: 23.784 g	• Lungs: Mononuclear perivascular, peribronchiolar, or subpleural aggregates, 2, MF.<br>• Thymus: Thymic cysts, MF.<br>• Kidneys: Tubular degeneration, 1, MF, cortical.<br>• Liver: Mononuclear perivascular/peribiliary infiltrates, 1, MF.<br>• Vagina: Mononuclear and neutrophilic infiltrates, 2, MF, submucosal/muscularis.<br>• Spleen: Plasmacytosis, 1, MF.<br>• Adrenals: Accessory adrenocortical nodule, F, UL. Subcapsular spindle cell hyperplasia, 2, MF, BL.<br>• Harderian gland: Acinar degeneration and porphyrin deposits, 2, MF, BL.
M2/ $^{177}Lu/^{225}Ac$ only (62.9 MBq/18.5 kBq)	• Bodyweight: 26.137 g<br>• The left axillary lymph node is cystic. The spleen is mildly enlarged.	• Lungs: Mononuclear perivascular, peribronchiolar, or subpleural aggregates, 3, MF. Alveolar histiocytosis, 2, MF.<br>• Thymus: Thymic cysts, MF.<br>• Kidneys: Mononuclear interstitial infiltrates, 1, MF, pelvic.<br>• Liver: Mononuclear perivascular/peribiliary infiltrates, 1, MF.<br>• Submandibular lymph node: Medullary plasmacytosis, 3, D. Lymphoid follicular hyperplasia, 2, MF.<br>• Urinary bladder: Mononuclear perivascular aggregates, 1, MF.<br>• Spleen: Plasmacytosis, 3, MF.<br>• Adrenals: Subcapsular spindle cell hyperplasia, 2, MF, UL.<br>• Harderian gland: Acinar degeneration and porphyrin deposits, 2, MF, BL. Histiocytic interstitial aggregates, 2, MF, UL.<br>• Other: Cystic left axillary lymph node: lymphatic ectasia 4, MF
M3/ $^{177}Lu/^{225}Ac$ only (62.9 MBq/18.5 kBq)	• Bodyweight: 25.371 g<br>• The spleen, submandibular, mesenteric, and axillary lymph nodes are moderately	• Lungs: Mononuclear perivascular, peribronchiolar, or subpleural aggregates, 2, MF.<br>• Thymus: Thymic cysts, MF.<br>• Kidneys: Mononuclear perivascular to interstitial aggregates, 2, MF, cortical and pelvic.<br>• Liver: Mononuclear and neutrophilic perivascular/peribiliary aggregates, 2, MF.<br>• Gallbladder: Mononuclear interstitial aggregates, 2, MF.<br>• Duodenum, jejunum, ileum : Plasmacytic and neutrophilic enteritis and serositis/peritonitis, 3, MF.

	enlarged. The left axillary lymph node is cystic.	• Mesenteric lymph node : Pyogranulomatous lymphadenitis with plasmacytic aggregates and fibrosis, 4, MF.<br>• Salivary glands: Mononuclear perivascular to interstitial aggregates, 1, MF.<br>• Submandibular lymph node: Medullary plasmacytosis, 2, D. Lymphoid follicular hyperplasia, 2, MF.<br>• Urinary bladder: Mononuclear perivascular aggregates, 2, MF.<br>• Vagina: Mononuclear and neutrophilic infiltrates, 2, MF, submucosal/muscularis.<br>• Ovaries: Pigment-laden macrophage infiltrates, 3, MF.<br>• Oviducts: Epithelial vacuolation, 3, MF.<br>• Spleen: Plasmacytosis, 3, MF.<br>• Pancreas: Mononuclear and neutrophilic perivascular to interstitial aggregates, 2, MF.<br>• Esophagus: Mononuclear and neutrophilic interstitial infiltrates, 1, MF, muscularis.<br>• Skin (flank): Acanthosis and hyperkeratosis, 2, MF. Mononuclear and neutrophilic dermatitis and mural folliculitis, 2, MF.<br>• Harderian gland: Mononuclear and neutrophilic adenitis, with acinar degeneration, atrophy, porphyrin deposits, and interstitial fibrosis, 4, MF, UL.<br>• Other: Mesentery: pyogranulomatous peritonitis, 4, MF. Popliteal lymph node: pyogranulomatous lymphadenitis, 4, MF. Cystic left axillary lymph node: lymphatic ectasia 4, MF
M4/ ^{177}Lu/^{225}Ac only (62.9 MBq/18.5 kBq)	• Bodyweight: 25.371 g<br>• The left axillary lymph node is cystic.	• Lungs: Mononuclear perivascular, peribronchiolar, or subpleural aggregates, 2, MF.<br>• Thymus: Thymic cysts, MF (slide 7).<br>Kidneys: Tubular degeneration, 1, MF, cortical. Mononuclear perivascular to interstitial aggregates, 2, MF, cortical and pelvic.<br>• Liver: Mononuclear perivascular/peribiliary infiltrates, 1, MF.<br>• Salivary glands: Mononuclear perivascular to interstitial aggregates, 1, MF.<br>• Urinary bladder: Mononuclear and neutrophilic cystitis, 3, MF. Mononuclear perivascular aggregates, 1, MF<br>• Vagina: Mononuclear and neutrophilic infiltrates, 2, MF, submucosal/muscularis.<br>• Spleen: Plasmacytosis, 3, MF.<br>• Pancreas: Mononuclear and neutrophilic perivascular to interstitial aggregates, 1, MF.<br>• Adrenals: Subcapsular spindle cell hyperplasia, 2, MF, BL.<br>• Skin (flank): Acanthosis and hyperkeratosis, 2, MF. Mononuclear dermatitis and mural folliculitis, 1, MF.<br>• Harderian gland: Mononuclear and neutrophilic adenitis, with acinar degeneration, atrophy, and porphyrin deposits, 3, MF, UL.<br>• Other: Cystic left axillary lymph node: lymphatic ectasia 4, MF
M5/ ^{177}Lu/^{225}Ac only (62.9 MBq/18.5 kBq)	• Bodyweight: 23.323 g	• Lungs: Mononuclear perivascular, peribronchiolar, or subpleural infiltrates, 1, MF.<br>• Thymus: Thymic cysts, MF.<br>• Liver: Mononuclear perivascular/peribiliary infiltrates, 1, MF.

		• Mesenteric lymph node: Medullary plasmacytosis, 1, D.<br>• Submandibular lymph node: Medullary plasmacytosis, 2, D. Lymphoid follicular hyperplasia, 2, MF.<br>• Oviducts: Epithelial vacuolation, 3, MF.<br>• Spleen: Plasmacytosis, 2, MF.<br>• Adrenals: Subcapsular spindle cell hyperplasia, 2, MF, UL.<br>• Harderian gland: Porphyrin deposits, 1, MF, BL.
F:Focal. MF: Multifocal: D: Diffuse. UL: Unilateral: BL: Bilateral. 1: Minimal. 2: Mild. 3: Moderate. 4: Marked		

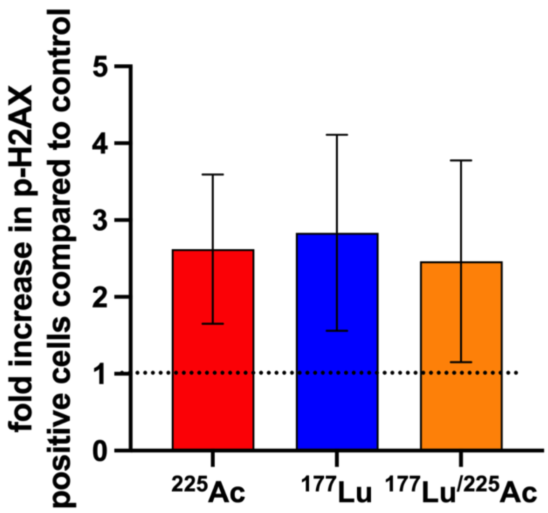

Figure S11: Quantification of p-H2AX in SW1222 xenografts 24 hours administration of ^{225}Ac-, ^{177}Lu-, or ^{177}Lu/^{225}Ac-DOTA-PRIT (74 kBq, 66.6 MBq and 62.9 MBq ^{177}Lu + 18.5 kBq ^{225}Ac, respectively). Results are displayed as fold increase compared non-treated control samples. In total, 2 tumor samples were analyzed per group. For each tumor sample, 5 areas were chosen at random and analyzed for number of p-H2AX positive cells.

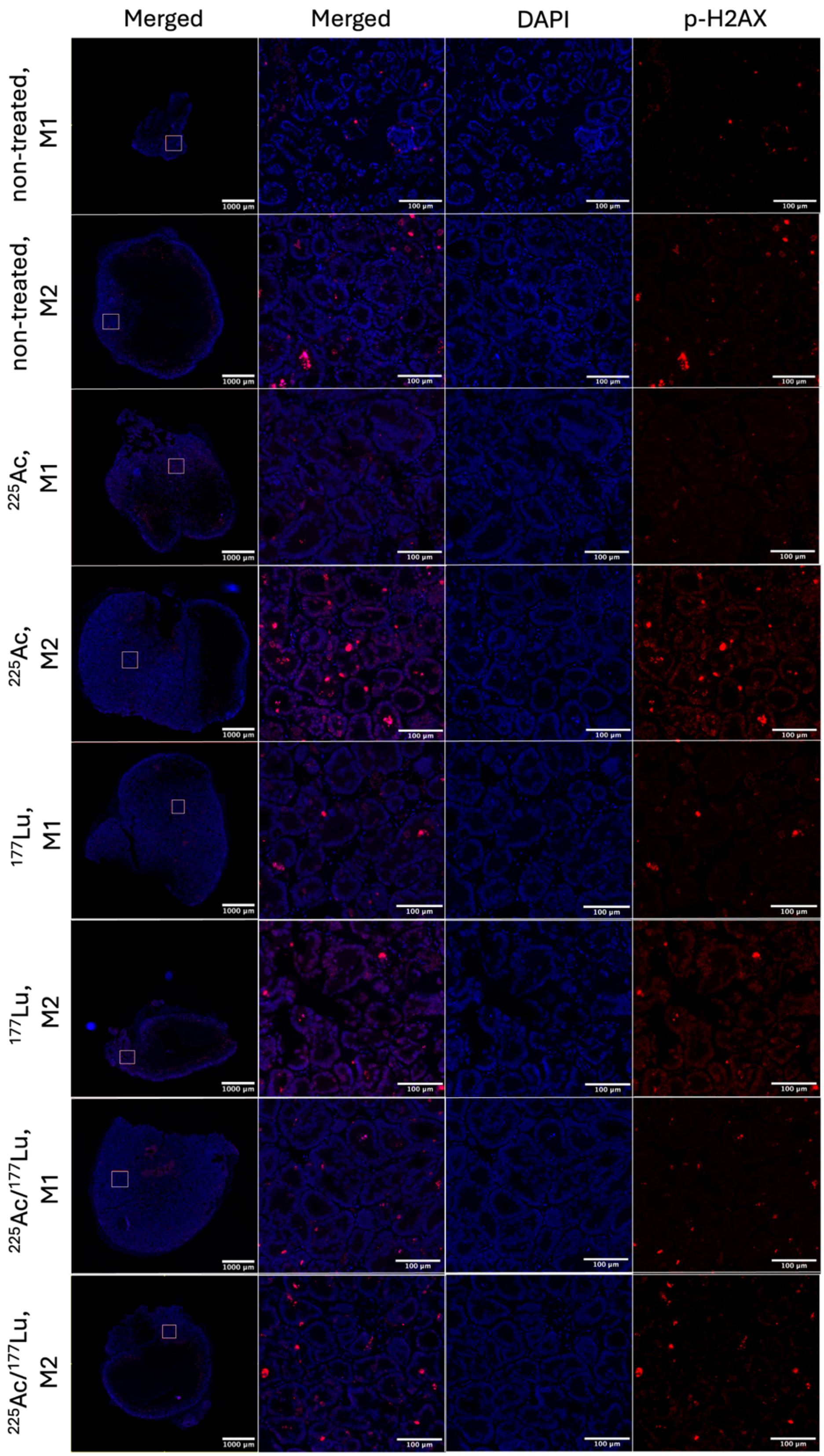

Figure S12: p-H2AX staining of SW1222 xenograft sections safter treatment with ^{225}Ac-, ^{177}Lu-, or ^{177}Lu/^{225}Ac-DOTA-PRIT. Xenografts were collected 24 hours after treatment with 66.6 MBq ^{177}Lu, 74 kBq ^{225}Ac or 62.9 MBq ^{177}Lu + 18.5 kBq ^{225}Ac.

qPCR of DNA damage and apoptosis related genes

Figure S13: Results from qPCR analysis of SW1222 tumor samples for the expression of DNA damage and apoptosis related genes after treatment with ^{177}Lu-DOTA-PRIT, ^{225}Ac-DOTA-PRIT or ^{177}Lu/^{225}Ac-DOTA PRIT using RT2 profiler PCR Array. Tumor samples were collected 24 h after injection of the radioligands (2 tumor samples/group). Mice were treated with therapeutic doses (66.6 MBq ^{177}Lu, 74 kBq ^{225}Ac or 62.9 MBq ^{177}Lu + 18.5 kBq ^{225}Ac) resulting in an estimated absorbed dose of 37-38 Gy to the tumor.

Jaggi, J. S., Seshan, S. V., McDevitt, M. R., LaPerle, K., Sgouros, G., & Scheinberg, D. A. (2005). Renal tubulointerstitial changes after internal irradiation with alpha-particle-emitting actinium daughters. *J Am Soc Nephrol*, *16*(9), 2677-2689. https://doi.org/10.1681/asn.2004110945
